# Offline dominance and zeugmatic similarity normings of variably ambiguous words assessed against a neural language model (BERT)

**DOI:** 10.3758/s13428-022-01869-6

**Published:** 2022-06-10

**Authors:** Katherine A. DeLong, Sean Trott, Marta Kutas

**Affiliations:** 1 Department of Cognitive Science, University of California, San Diego (UCSD), 9500 Gilman Drive, La Jolla, CA 92093-0515 USA; 2 UCSD Center for Research in Language, La Jolla, CA USA; 3 UCSD Department of Neurosciences, La Jolla, CA USA; 4 UCSD Kavli Institute for Brain and Mind, La Jolla, CA USA

**Keywords:** Semantic ambiguity, Homonyms, Polysemes, Dominance norming, Similarity rating, Zeugma

## Abstract

For any research program examining how ambiguous words are processed in broader linguistic contexts, a first step is to establish factors relating to the frequency balance or dominance of those words’ multiple meanings, as well as the similarity of those meanings to one other. Homonyms—words with divergent meanings—are one ambiguous word type commonly utilized in psycholinguistic research. In contrast, although polysemes—words with multiple related senses—are far more common in English, they have been less frequently used as tools for understanding one-to-many word-to-meaning mappings. The current paper details two norming studies of a relatively large number of ambiguous English words. In the first, offline dominance norming is detailed for 547 homonyms and polysemes via a free association task suitable for words across the ambiguity continuum, with a goal of identifying words with more equibiased meanings. The second norming assesses offline meaning similarity for a partial subset of 318 ambiguous words (including homonyms, unambiguous words, and polysemes divided into regular and irregular types) using a novel, continuous rating method reliant on the linguistic phenomenon of zeugma. In addition, we conduct computational analyses on the human similarity norming data using the BERT pretrained neural language model (Devlin et al., 2018, *BERT: Pre-training of deep bidirectional transformers for language understanding.* ArXiv Preprint. arXiv:1810.04805) to evaluate factors that may explain variance beyond that accounted for by dictionary-criteria ambiguity categories. Finally, we make available the summarized item dominance values and similarity ratings in resultant appendices (see supplementary material), as well as individual item and participant norming data, which can be accessed online (https://osf.io/g7fmv/).

**Supplementary Information:**

The online version contains supplementary material available at 10.3758/s13428-022-01869-6.

One-to-many word-to-meaning mappings are ubiquitous, with the majority of words in English having multiple meanings or senses (Eddington & Tokowicz, 2015; Rodd, 2018). In theory, subsequent exponential ambiguity of phrases, sentences, discourses, or any larger chunk of language would seem to make comprehension challenging if not impossible; in reality, humans negotiate such ambiguities with relative ease. This is due to the roles of intra- and extralinguistic contexts in the activation of different word meanings or senses. However, the nature of on-the-fly interactions of context with word representations in semantic memory, the kinds of and how many ambiguous word meanings or senses are activated in the brain, with what specificity, to what degree, and when or for how long, are questions with complex answers, some yet unsolved.

Decades of behavioral and eye tracking studies investigating word recognition and meaning access have employed ambiguous word paradigms, which have identified the primary influences on isolated ambiguous word interpretation as (1) the relative frequency or *dominance*^1^ of a word’s meanings or senses, and (2) the *similarity* of a word’s multiple meanings or senses to each other. Much of this work is grounded in a “mental lexicon” framework—the idea that the brain stores word information in neural dictionary-like entries for which meanings, pronunciations, and syntactic properties (among other things) can be looked up. Early results from ambiguous word studies were used to support theories of modularity (e.g., Swinney, 1979) but also interactivity (Kellas & Vu, 1999; McClelland & Rumelhart, 1981; Morton, 1969; Vu & Kellas, 1999). However, early eye tracking data (e.g., subordinate bias effects; Duffy et al., 1988; Pacht & Rayner, 1993; Rayner & Duffy, 1986) did not accord with a strong version of either view and led to hybrid models like the popular *reordered access account*. This model posits that except in highly constraining contexts, word meanings are initially exhaustively—not selectively—accessed, but with frequent and/or contextually congruent meanings selected very quickly postlexically (Binder & Rayner, 1998; Rayner et al., 1994; Swinney, 1981). This model had considerable support across a variety of paradigms (e.g., Kambe et al., 2001; Reichle et al., 2007; Sereno, 1995; Sereno et al., 2003; Sheridan et al., 2009; Swaab et al., 2003). Outside of larger linguistic contexts, however, dominant meanings were found to prevail (e.g., Duffy et al., 1988).

Until more recently, homonyms (e.g., *batter*, *mint*, *port*, *colon*) were the primary type of ambiguous word employed in psycholinguistic studies of word representations, online meaning activation, and language processing. Homonyms are typically operationalized by their multiple semantically distinctive meanings of different origins, listed under separate dictionary entries. It is only by chance that homonyms’ different meanings share the same orthographic and phonological forms. Along the dimension of meaning dominance, homonyms can have relatively equibiased meaning frequencies (e.g., *calf* [*cow/leg*], *colon* [*punctuation/organ*], *pitcher* [*container/baseball*]), or they can be biased, with one obviously **dominant** meaning (e.g., *ball* [***throw****/dance*]*, nag* [***pester****/horse*], or *pen* [***writing instrument****/enclosure*])*.*

Homonyms, however, represent just one type of ambiguous word, and the aforementioned work only rarely employed another type—polysemes—even though they outnumber homonyms in English by far (84% to 7%, according to Rodd et al., 2002). Polysemes (e.g., *cotton*, *church*, *slate*, *speaker*) are word forms with multiple related senses deriving from a common origin that are often operationalized as having senses listed under a single dictionary entry. Polysemous senses vary in the nature of their semantic relations (e.g., *bottle* [*container/contents*], *chicken* [*animal/meat*], *fortune* [*money/luck*], or *straw* [*drinking/plant fiber*]). Despite being related, they may have semantic features that are quite different (Klein & Murphy, 2001); for instance, when comparing *freshly planted cotton* and *blue striped cotton*, although it is clear that both senses of *cotton* have something in common, they vary along dimensions of living/non-living, natural/manmade, color, texture, function. These characteristics make polysemes particularly interesting test cases for language processing/ambiguity experiments, although they have traditionally been less utilized experimentally than homonyms—possibly due to known difficulties in distinguishing polysemy from related phenomena like vagueness (Geeraerts, 1993; Tuggy, 1993).

Experimental tests of polysemes have begun to attract more attention in recent years, and with their greater semantic similarity and feature overlap, it is, for instance, unclear how well models like reordered access apply to polyseme processing. In fact, several studies have failed to observe dominance/frequency effects for polysemes (Frazier & Rayner, 1990; Frisson & Pickering, 1999). Current theoretical debates on polyseme meaning retrieval center on whether polysemes are stored and retrieved with separate representations for each of their multiple (related) senses, similar to how homonyms have traditionally been proposed to be represented; or, whether they are stored and processed qualitatively differently than homonyms, with overlapping multiple sense representations (for overviews, see Brocher et al., 2018; Eddington & Tokowicz, 2015).

In order to study how ambiguous words—both homonyms and polysemes—are processed in wider contexts, whether online or offline, it is essential to establish the nature of their multiple meanings and the meanings’ relations to each other. Identifying these factors also serves a second-order goal: a better understanding of how human comprehenders represent and process the meanings of ambiguous words would aid natural language processing (NLP) practitioners in developing more humanlike models of word meaning, particularly for tasks that explicitly involve adjudicating between distinct meanings of an ambiguous word, such as word sense disambiguation (Haber & Poesio, 2020; Loureiro et al., 2020; Trott & Bergen, 2021).

With the bulk of work on ambiguous word comprehension having centered on homonyms, there are far fewer published resources for assessing polyseme sense frequencies and/or similarities (but see Foraker & Murphy, 2012; Klepousniotou & Baum, 2007). In contrast to homonymous words that have multiple unrelated meanings, discerning different but related senses of polysemes (and in turn their individual frequencies) is less straightforward (Tuggy, 1993). This is perhaps complicated further by the possibility that there are different types of polysemy, as described in theoretical linguistics and experimental work (e.g., Apresjan, 1974; Brocher et al., 2016; Moldovan, 2021; Rabagliati & Snedeker, 2013). For instance, Apresjan (1974) classifies polysemous words as metonymic (regular) or metaphoric (irregular). Frisson and Pickering (1999) describe metonymic constructions as ones in which “one salient aspect of an entity is used to refer to the entity as a whole or to some other part of the entity.” The relationships between the meanings of metonymic polysemes are often highly systematic across the lexicon and even across languages (Apresjan, 1974; Lakoff, 1987; Srinivasan & Rabagliati, 2015), and typically reflect regular *functional* patterns; for instance, container versus its contents (*bottle*, *bowl*); animal versus its meat (*chicken*, *fish*); organization versus publication-produced-by-organization (*newspaper*, *magazine*); metal/gem versus color-typical-of-a-metal/gem (*gold*, *copper*, *topaz*, *emerald*). Metaphorical polysemes, on the other hand, are ones with analogical relations, where one sense is literal and another figurative, and the relations are unpredictable and not necessarily obvious or easy to identify; for instance, *leg* (belonging to an animal or table), *pig* (an animal or sloppy person), or *star* (a celestial body or famed individual). However, as Klepousniotou and Baum (2007) describe, there is neither a sharp line distinguishing homonyms from polysemes, nor is there one that clearly delineates metaphoric from metonymic polysemes.

Take, for example, a word like *panel*—a polyseme, according to the single dictionary entry criterion (*Wordsmyth* online dictionary; https://www.wordsmyth.net). Multiple senses of this word include: (1) *a section, as of a door, wall, or the like, usually flat and often set apart from the surrounding area by being raised, recessed, or decorated*, and (2) *a group of persons assembled for a particular purpose, as to investigate or evaluate something*. These senses seem distinct but have a mutual historical derivation; in other words, it is not coincidental that they share a single phonological word form (which would lead to their being categorized as homonyms). In addition, both senses seem literal, at least in current, everyday usage, which might endorse the classification as a more metonymic relation; however, there is also no regular, productive relation here, which undermines their classification as metonymic. Without access to *panel*’s etymology, it is not clear that contemporary English language users would intuit which sense was derived from which. In fact, metaphorical shifts are not always apparent to language users (Apresjan, 1974; Yurchenko et al., 2020). Although these two senses of *panel* index very different sets of information, the dictionary specifies—and perhaps the average language user consciously or unconsciously grasps—that these senses are not completely distinctive (i.e., are not homonyms), but rather have some semantic overlap or shared origin. What is unknown, and the outstanding question that lies at the heart of our larger experimental research program, is how the brain stores, organizes, and accesses information about such ambiguous words during real time comprehension. Because dictionaries do not reflect the psychological realities of language users in natural environments, and neural organization is unlikely to be characterized by dictionary-like entries cataloged along etymological lines, alternative methods are required to assess the range of meaning frequencies and similarities.

If we consider that these different ambiguous word types may lie along a continuum of meaning or sense similarity (e.g., Eddington & Tokowicz, 2015; Klepousniotou, 2002; Tuggy, 1993), homonyms would represent the most ambiguous end with their multiple unrelated meanings, and unambiguous words would anchor the opposite end, with words operationalized by their single-meaning dictionary entries (e.g., *monk*, *midnight*, *icicle*, *easel*, *wig*). In terms of their more related senses, the different classes of polysemes would lie somewhere between these two extremes. Importantly, differences in the type or degree of word ambiguity might correspond to differences in how words are processed online and stored/organized in the brain (e.g., Klepousniotou et al., 2008; Klepousniotou et al., 2012). Thus, accounting for these offline lexical factors becomes a crucial first step.

With these considerations in mind, we set out to conduct two norming studies to generate, norm, and select a set of words with variable degrees of ambiguity that could eventually be used to test the proposal that words function as ‘cues to meaning’ (Elman, 2004). Instead of static entries in a dictionary that must be looked up, Elman’s framework conceptualizes words as inputs to a dynamic, context-sensitive network, such that *meaning* is equivalent to the trajectory through this network’s state-space elicited by a particular wordform, in a particular context. We sought words that spanned the ambiguity continuum but that also had relatively balanced meaning/sense frequencies. Although the prominent role of meaning dominance and its effects on ambiguous word interpretation (particularly for homonyms) have been well documented over the years, less is understood about the nature of dominance for polysemous senses of words. Given our interest in exploring questions about words that span a continuum of ambiguity, combined with our laboratory’s research focus on the role of (sentence) context in stimulus interpretation and meaning construction, we aimed to generate a list of ambiguous words for which differences in dominance values—as a potential source of variability—were minimized, while the meanings or senses of individual words spanned a wide range semantic similarity.

Study 1 is a meaning/sense dominance norming study designed to assess isolated (i.e., outside explicitly disambiguating or informative sentential context) ambiguous words’ meaning/sense dominance using a method tried and proven with homonyms. This involved classifying associates provided by participants for individual ambiguous words based on the word meanings or senses to which they are related. Study 2 is a similarity norming designed to quantify the relatedness of words’ meanings/senses using a novel rating paradigm that relies on the linguistic phenomenon of *zeugma*. These methods allow flexibility for assessing meaning distance/similarity over a range of ambiguous word types, but in particular they offer sensitivity to the nuanced sense distinctions of polysemes.

Finally, as noted earlier, these similarity ratings serve a useful second-order function in helping to evaluate computational models of word meaning. Although there are many lexical resources containing similarity judgments of different words in isolation (e.g., *dolphin* vs. *shark*; Bruni et al., 2014; Gerz et al., 2016; Halawi et al., 2012; Hill et al., 2015; Taieb et al., 2020), there are comparatively fewer that contain graded relatedness judgments for different meanings of the same ambiguous word, with inflection and part-of-speech controlled (Haber & Poesio, 2020; Trott & Bergen, 2021). Thus, in Experiment 2, we compare the human similarity ratings to two measures obtained from BERT (Devlin et al., 2018), a large pretrained neural language model.

## Study 1: Ambiguous word meaning/sense dominance norming

Our goal in this first of two norming studies was to quantify meaning or sense frequency for a large number of ambiguous words spanning homonyms and polysemes, from which a subset of meaning/sense-balanced items could be selected for similarity norming and, for our purposes, eventual sentence embedding in word-by-word reading experiments. One method that has been used to establish meaning dominance of ambiguous words is to ask participants to rate the relative familiarity or frequency of the meanings or senses of ambiguous words on a continuous scale (e.g., Klepousniotou et al., 2012) or by estimating relative percentages of homonym meanings (e.g., Armstrong et al., 2012). However, we thought that estimating frequency of meaning/sense usage might be disproportionately difficult for the polysemes, with their more subtle semantic sense distinctions. Instead, we considered another commonly used method, which begins by collecting and analyzing the free associates generated to ambiguous words from new or existing norms (e.g., Mirman et al., 2010; Nelson et al., 1980; Twilley et al., 1994). A separate group of raters then classifies the responses based on their associated word meanings. While this method has been used frequently to assess homonym dominance, we determined that the same general approach would be well suited for a broad range of ambiguous word types, including polysemes. This method, moreover, has been shown to generate fairly consistent measures of dominance across a variety of studies (see Twilley et al., 1994).

### Method

#### Participants

Ninety-seven native English-speaking UCSD undergraduates (80 female, 17 male)^2^ between the ages of 18 and 24 years took part in an online dominance norming study for course credit. Participants were recruited from the UCSD Psychology Department subject pool’s online scheduling system. Informed consent was obtained from all participants after the norming procedure had been outlined for them.

#### Materials

For our initial selection of ambiguous words to norm, we relied on published resources (in particular for homonyms) to seed our list (e.g., Armstrong et al., 2012; Gorfein et al., 1982; Nelson et al., 1980; Twilley et al., 1994). Since acquiring relatively meaning/sense-balanced words was a goal, we focused on studies that had either published dominance measures or had included words labeled as having equibiased or balanced meanings. Armstrong et al. (2012) report that most homonyms have biased meaning frequencies, with the relative dominance of the most frequent meaning often exceeding 75% (a common cut-off value for classifying relatively balanced versus unbalanced homonyms). Accordingly, we used the 75% criterion to guide our selection of more balanced ambiguous words, in particular for homonyms, for which more resources were readily available.

For polysemes, we identified candidate words by consulting the more limited experimental literature in which polysemous words were specifically examined or discussed (e.g., Durkin & Manning, 1989; Foraker & Murphy, 2012; Klein & Murphy, 2001; Klepousniotou & Baum, 2005, 2007). Given the scarcity of reported polyseme dominance ratings, we supplemented our candidate word list with items taken from theoretical linguistics work on polysemes (e.g., Apresjan, 1974; Nunberg, 1979), as well as by expanding from known metonymic (regular) polysemic relations (e.g., substance/color, container/contents, building/organization). We also mined resources describing or testing “ambiguous words”, which did not specifically differentiate between homonyms and polysemes (e.g., Duffy et al., 1988; Gilhooly & Logie, 1980b).

A total of 558 ambiguous words (Appendix 1, Column A in supplementary material) were selected for dominance norming. Utilizing the online *Wordsmyth* dictionary (https://www.wordsmyth.net), a resource often employed for semantic ambiguity research (e.g., Armstrong & Plaut, 2011; Armstrong et al., 2012; Rodd et al., 2002), we began by labeling these ambiguous words as either homonyms or polysemes based on whether there were separate dictionary entries for multiple meanings (homonyms) or if all senses were listed under a single entry (see Appendix 1, Column B in supplementary material). At this point in the experiment, these category labels merely served as reference points, as one of the goals for the current study was to characterize ambiguous words’ most frequent meanings based on human data rather than dictionary criteria that do not necessarily reflect the familiarities of words’ multiple meanings/senses. In addition, because homonyms can and often do have multiple senses listed under at least one of their distinct meanings, (e.g., *bridge* can have homonymous meanings of ‘*a structure that extends*’ and ‘*a card game*’, but under the first meaning can also have polysemous senses relating to, for instance, ‘*artificial teeth*’ or ‘*part of a human nose*’), we also indicated this information in Column B of Appendix 1 in supplementary material. In fact, of our dominance-normed items, all but 8 (BOWLER, CAPE, FORD, MUSTANG, NOODLE, NOVEL, POKER, PUPIL) of the dictionary-criteria homonyms also had multiple senses listed under at least one of their *Wordsmyth*-listed meanings. It is interesting to note that to our knowledge there has been little discussion in the experimental literature about this “polysemy of homonyms”. Typically, when homonyms and polysemes have been contrasted experimentally, the homonymous meanings are taken to be the superseding ones. As we describe in the following section, our procedure for determining ambiguous words’ most frequent meanings was dictated by participants’ responses, and when relevant, polysemous senses of dictionary-criteria homonyms were considered.

#### Norming procedure

We conducted a web-based dominance norming to assess ambiguous words’ meaning frequencies by presenting putative homonyms and polysemes without any surrounding (preceding or following) context. Individuals were instructed to “type the first word/phrase that comes to mind, and then type a second word/phrase that comes to mind.” Words were presented in uppercase font to avoid biasing against meanings or senses that when capitalized could constitute a proper noun or acronym. Participants were informed to go with their initial instincts and to answer as quickly as possible, without repeating words in their first and second responses.

For example, an ambiguous word stimulus with possible participant-generated word associates might be:

- BEAR: ___*grizzly*___
- BEAR: ___*teddy bear*___

Other potential responses might be: *polar, stuffed, endure, put up with, brunt, burden, witness, weight, brown, furry, fierce, Pooh, honey, tree, claw,* etc. Participants were not informed that the words they would be encountering were ambiguous, nor were they instructed to provide norming responses that did or did not draw from separate meanings or senses of the words. Thus, an individual might provide two responses that fell under a single meaning or word sense (e.g., *grizzly* or *polar*) or multiple meanings (e.g., *furry*, *witness*). Participants were instructed to type the word “blank” if they were unable to provide an answer.

Each norming participant was presented with one of two lists that included 319 ambiguous words comprised of a mix of homonyms and polysemes, with a subset of items included in both stimulus lists. Stimuli were presented in unique, randomized orderings for each participant. Participants generally complied in providing two norming responses for the majority of words presented to them. Aiming for a sample size roughly consistent with or greater than those from previous dominance normings (e.g., Gilhooly & Logie, 1980a; Gorfein et al., 1982; Nelson et al., 1980), each ambiguous word was normed by between 47 to 95 participants, with an average of 106 responses collected per word (range: 93–189, *SD* = 33.4).^3^ The individual participant and item dominance norming responses from Study 1 may be accessed at https://osf.io/g7fmv/.

#### Calculating meaning/sense dominance

##### Determining the most frequent norming responses

Following data collection, obvious spelling errors and typos in the participant responses were corrected. As a first step toward determining the relative frequencies of the ambiguous words’ different meanings/senses, we began by generating a list of candidate definitions based on the most frequent norming associates provided. Since each normed word had a large number of open-ended, highly variable, sometimes multiword responses, a simple alphabetical sorting would (a) not have grouped responses like *game* and *card game* together, and (b) would have required time consuming manual review to determine the most common responses. To facilitate sorting, we adopted the novel (to our knowledge) approach of utilizing an online word cloud visualization tool. Generally, word clouds are used to visualize input from text documents, with their outputs displaying the most frequent of those words, with font sizes proportional to the individual words’ relevance or frequency, sometimes clustered semantically. The free online semantics-preserving word cloud visualization application we used was “Semantic Cloud” (http://wordcloud.cs.arizona.edu/). Using this tool entailed pasting the complete sample of norming responses for each word into the application to generate the visual output. From the output, we were able to rapidly identify the most frequent word associates provided as norming responses for each ambiguous word based on font size. The word cloud tool also offers an option for visually sorting the input words based on their frequency rank, which expedites the process of tallying and determining the most frequent responses. For instance, for the 94 norming responses provided by participants for the ambiguous word APPEAL, which were then pasted into the word cloud tool, we were quickly able to see that *court, interest, attractive, nice,* and *overturn* were the most commonly provided responses. We note that in the mapping of norming responses to word cloud output, multi-word entries are not preserved. For our purposes—using the tool to easily identify the most frequent associates as a step toward determining a set of candidate meanings—this level of imprecision was unproblematic. For instance, if several norming respondents provided *Golden Gate* as a response to the prompt *BRIDGE*, the first author was able to ascertain from the identical frequencies of *golden* and *gate* in the word cloud output that these words were likely part of a compound phrase, at which point the norming responses could be referred to for hand checking.

##### Determining the most frequent meanings or senses

Once the most frequent norming responses for each ambiguous word were determined (the number ranged from 2 to 8, mean = 5.5, *SD* = 0.9), the next step was to use them to ascertain the most frequent associated meanings or senses. Utilizing multiple resources, including the online *Collins COBUILD* dictionary (https://www.collinsdictionary.com/dictionary/english), Google’s English dictionary provided by *Oxford Languages*, and the *Wordsmyth* online dictionary, we selected 2–5 easy-to-understand, succinctly worded definitions or senses under which the most frequent norming associates fell (mean number of definitions = 2.30, *SD* = 0.55).^4^ Typically, the definitions chosen were the top dictionary entries for those words, over multiple entries for homonyms or within a single entry for polysemes. Although this process was sufficient for identifying the candidate definitions or senses for most of the norming items, sometimes the frequent norming responses alluded to alternative meanings not listed in the dictionary (e.g., *AMAZON* as in the company, *BOLT* as in the name of a dog from the Disney movie of the same name, or *HOOD* as in a shortened, slang version of the word neighborhood.)

##### Definition/sense rating and dominance score calculation

The next step was to categorize every participant norming response for each word by its meaning or sense. Two raters (the first author, Rater 1, and a fellow lab researcher, Rater 2) worked individually, deciding whether each norming response corresponded either with (a) one of the 2–5 selected definitions, or (b) with multiple, undifferentiable, or none of the 2–5 definitions. After the rating process was completed, the total number of ratings per item aligning with any single definition were tallied, along with the total number of rated responses corresponding with the 2–5 selected senses/definitions, and the percentages were calculated. If the two raters assigned different meanings to a norming response, each rating was factored into the count for the respective meanings. The two definitions/senses with the highest percentages were identified and a dominance score (per Armstrong et al., 2012) was calculated by dividing the percentage difference by the highest definition/sense percentage.

The resultant dominance scores for individual items could range from 0.00 to 1.00, with a score of 0.00 representing an ambiguous word with its most frequent senses perfectly balanced, and a score of 1.00 indicating that one sense was completely dominant over the other. See Appendix 1, Column I in supplementary material.

#### Interrater reliability

Interrater reliability was calculated for the assignment of the norming responses to different meanings or senses of ambiguous words. Of the total number of norming responses, 75.7% were assigned meanings by both raters, with the remaining 24.3% assigned meanings by a single rater. Of the items assigned to meanings by both raters, there was 94.9% interrater consistency. These results indicate that the meaning/sense classification was overall highly reliable.

#### Sample Item

To demonstrate the entire process for arriving at an ambiguous word’s dominance score, we here outline the steps taken for an individual item: Example: APPEAL.

1. APPEAL was initially classified as a polyseme according to the criteria of there being multiple senses that fell under a single dictionary entry.
2. Word associates were obtained through norming from 47 participants, who provided two responses each.
3. Ninety-four norming responses were pasted into the word cloud software and *court*, *interest*, *attractive*, and *nice* were revealed to be the most frequently provided associates.
4. Based on the top associates provided in the norming, two dictionary senses were identified as encompassing the most frequent responses: *APPEAL* SENSE (1) *the power to attract, please, stimulate, or interest*, and *APPEAL* SENSE (2) *judicial review by a superior court of the decision of a lower tribunal*.
5. The two raters then determined under which dictionary sense each norming response fell, with 63% of the responses aligning with SENSE 1 and 37% with SENSE 2. The two raters’ categorizations were highly consistent.
6. A dominance score was then calculated by dividing the percentage difference of the two most frequent senses by the highest sense percentage ([0.631 − 0.369]/0.631 = 0.415).

### Analysis and results

#### Data rejections

Of the 558 dominance-normed ambiguous words, there were 11 items that we did not rate and for which dominance scores were not calculated (see asterisked items in Column A of Appendix 1 in supplementary material), leaving 547 remaining ambiguous words. The reasons for rejecting these 11 items varied. For some words, a majority of the norming responses could be associated with multiple meanings or senses, and thus it was impossible to assign them to a single definition. In other cases, all associates aligned with a single definition. In two cases there were problems with the words supplied in the norming: CHANEL was read as CHANNEL by some participants, who therefore provided associates for the wrong word; ROSES (not an ambiguous word) was inadvertently included by experimenters instead of ROSE (a homonym).

#### Dominance values

Although there were numerous items for which the range of norming responses aligned with more than two meanings or senses (our initial experimenter tallies allowed for up to 5), over the set of 547 ambiguous words, the mean percentage of responses aligning with the two most frequently related meanings/senses for each item was 97% (range: 0.56–1.00, *SD* = .07). Thus, in most cases, the top two meanings/senses accounted for nearly all of the norming associates provided.^5^

**Fig. 1:**
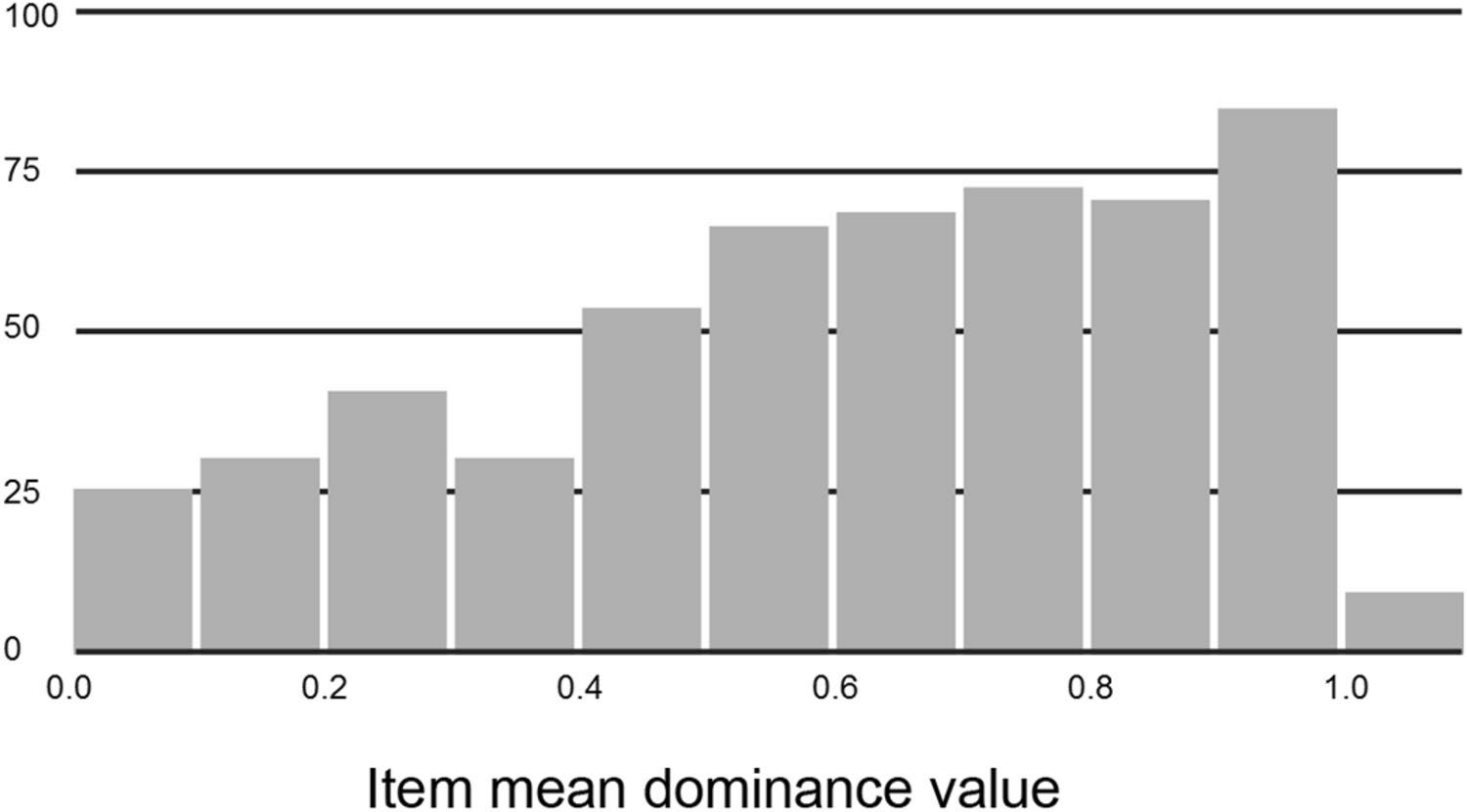
Frequency distribution of dominance values for 547 ambiguous words

The range of dominance values for the 547 ambiguous words was 0.00 to 1.00 (mean = 0.61, *SD* = 0.27). Of these, 349 words had dominance values ≤0.75, a typical cutoff for classifying ambiguous words as being more or less meaning/sense-balanced. See Fig. 1 for a frequency distribution of item mean dominance values. There were a number of items for which the dominance norming responses did not align with the homonym/polyseme classification based on dictionary criteria (indicated in Column J of Appendix 1 in supplementary material). In many instances, this was because one of the dictionary homonym meanings was obscure or infrequent, with norming respondents not supplying any associates related to that meaning (e.g., *BOXER*, *CARD*, *CHIP*, *DEAL*). In other cases, word meanings or senses were interpreted by some as proper names or acronyms (e.g., *BOND*, *MUSTANG*, *SUBWAY*, *NIRVANA, ACT*), which were not listed in the dictionary. For our purposes, we identified and labeled these ambiguous word types based on the top two meanings/senses associated with the norming responses; we provide our reasons for these shifts in Column K of Appendix 1 in supplementary material.

One consideration in soliciting two responses per item in the current study is that it diverged from more traditional single response paradigms (e.g., Rodd et al., 2002; Twilley et al., 1994). We note, however, that others have solicited multiple associates in dominance norming tasks; for instance, Brocher et al. (2016) and Brocher et al. (2018) supplied blanks to solicit 5 responses per participant. Our approach of soliciting two associates, then, was a compromise between the single and many-response methods. In addition to providing an increased amount of data, we thought that the multiple-blank approach was appropriate given our interest in studying relatively meaning/sense-balanced words. However, a potential concern is this may have led participants to adopt certain response strategies; for instance, the two blanks could have acted to cue words’ ambiguity, leading participants to assume that semantically different responses were required or could have led respondents to “double down” particularly on dominant meanings. Some support for this point comes from Twilley et al. (1994), who suggest that requiring multiple associations may make responses especially prone to strategic factors. In particular, the authors cite a norming study by Gorfein et al. (1982) in which participants were asked to produce four associations to homographs. In that study, 82% of participants produced associations to the same meaning on their first and second responses. To examine the degree of “doubling down” in our data, we conducted a post hoc examination to ascertain, if—like Gorfein et al. (1982)—there was a tendency for individual participants to produce associates linked to the same meanings on their first and second responses. Although we cannot know whether conscious or unconscious strategies were employed by our experimental participants, a tally of the dominance norming data revealed that 46% of individuals’ first and second responses to items were associated with the same meanings while 54% were associated with different meanings. These relatively balanced percentages suggest that there was not a consistent strategy employed by participants in providing free responses in our study.

#### Conclusions

In sum, the present norming study applied a tested method of assessing meaning dominance for homonymous words to a wider range (than is typically described in the literature) of ambiguous words that included polysemes. The results yielded lists of associative responses that were categorized by raters according to their meanings or senses. The relative frequencies of the most common senses or meanings for each word were then calculated, yielding a resultant list of dominance values for 547 ambiguous words. Although our main purpose for assessing dominance values was for use in planned written sentence comprehension studies, the data collected via this norming are likely to be useful to other researchers. Through this report, our results will be available to anyone interested in evaluating meaning/sense frequencies not only of ambiguous words with highly divergent multiple meanings but also of those whose senses are relationally or historically linked. This could be especially useful because (a) there are very few existing polyseme dominance norms, and (b) as our norming findings show, traditional dictionary entry criteria for assessing multiple meanings or senses do not always accurately reflect contemporary language users’ knowledge of or levels of familiarity with different semantic senses.

## Study 2: Ambiguous word meaning/sense similarity norming

In addition to determining the most frequent meanings/senses of isolated ambiguous words through dominance norming, we also set out to assess meaning and sense similarity. It was clear from the dominance norming that dictionary criteria for categorizing ambiguous words as either homonymous or polysemous did not always correspond with the meanings most familiar to our norming respondents (refer to Column J of Appendix 1 in supplementary material). For instance, sometimes words listed in the dictionary as homonyms elicited norming responses that aligned with only one word meaning or multiple senses of that meaning, with other listed meanings being arcane, obscure, or infrequent (e.g., *CARD*, as in *wool*; *CHIP*, as in *chirp*; *DEAL*, as in *planks*). Yet other words turned out to have norming responses associated with a sense or meaning not listed in the dictionary, sometimes because that sense represented the name of a contemporary product or brand (e.g., *MUSTANG*, *NIRVANA*, *SUBWAY*). There were still other dictionary-criteria polysemes whose shared origins may be unknown or opaque to the average undergraduate, with contemporary semantic senses that seem quite distinct (e.g., *PANEL*, as in *section* or *committee*; *CHARM* as in *charisma* or *ornament*; *STOCK*, as in *investment* or *inventory*). These polysemes tended to be ones considered irregular or having senses that represented more metaphorical relations (Apresjan, 1974). Apresjan (1974) and Klepousniotou and Baum (2007) suggest that irregular/metaphorical polysemy may be closer to homonymy than metonymic/regular polysemy, with Klepousniotou et al. (2012) describing metaphorical polysemes as perhaps lacking a fixed status in a continuum of lexical ambiguity; rather, these are words that may be in transition from generated senses to separate meaning representations. Metonymic/regular polysemes tend to exhibit greater sense contiguity and relatedness, with a variety of relation types that extend productively across broad categories of lexical items (e.g., container/contents [*VASE*, *BAG*]; animal/food [*CHICKEN*, *LOBSTER*]; producers/products [*MOZART*, *NEWSPAPER*]).

These different examples highlighted by the dominance norming led us to consider a further division of the ambiguous words for our similarity norming. Instead of sorting the ambiguous words into only two categories (homonyms and polysemes) as we did in the dominance norming, we further subdivided the polysemes into regular (metonymic) and irregular (metaphorical) polysemes, following the condition breakdowns in the experimental work of Klepousniotou and colleagues (e.g., Klepousniotou, 2002; Klepousniotou & Baum, 2007; Klepousniotou et al., 2012) among others. In addition, we decided to anchor our ambiguous word list with items arguably considered unambiguous (with single dictionary meanings/senses). With four levels of lexical ambiguity then (homonyms, irregular polysemes, regular polysemes, and unambiguous), the current norming offers the opportunity to observe whether the ratings for such linguistic categories align with patterns of increasing similarity ratings similar to those demonstrated in relatively limited experimental work (e.g., of Klepousniotou and colleagues), as well as the kind of ambiguity continuum proposed by theoretical linguists (e.g., Apresjan, 1974). In both cases, the order from least to most meaning similarity is homonyms, irregular (metaphoric) polysemes, regular (metonymic) polysemes, and unambiguous words. Ultimately, this second norming would also allow us to go beyond dictionary criteria for assessing meaning/sense similarity, providing contemporary U.S. English user-generated data about the relatedness of words’ multiple meanings/senses, across a range of ambiguous word types.

### Rating the similarity of ambiguous word meanings/senses

The goal was to assess the meaning similarity of the two most dominant meanings/senses of each ambiguous word as determined through the dominance norming. Some traditional approaches to evaluating the semantic similarity of ambiguous word meanings are for norming participants to judge the relatedness of two tokens of the same word used in different sentence or word pair contexts or through comparison and relatedness ratings of the multiple definitions (e.g., Brocher et al., 2018; Klein & Murphy, 2002; Rodd et al., 2002). For instance, individuals might be asked to compare and rate the similarity of the meanings of *cotton* in phrases such as ‘*the farm owners discussed the cotton . . .*’ versus ‘*the fashion designers discussed the cotton . . .*’ (Foraker & Murphy, 2012), or in word pairs such as ‘*marinated lamb*’ versus ‘*friendly lamb*’ (Klepousniotou et al., 2008).

For the current similarity norming, we considered modifying these more traditional approaches slightly, by still testing different word meanings/senses in strongly biasing contexts, but in a way that could maximize sensitivity across the range of homonyms and both classes of polysemes, and test in a manner by which explicit decision making is supported by implicit participant reactions to meaning similarity or differences. As a method for assessing a continuum of ambiguity, we thus considered the linguistic phenomenon of *zeugma*. *Zeugma* is defined as ‘*the use of a word to modify or govern two or more words usually in such a manner that it applies to each in a different sense or makes sense with only one*’ (*Merriam-Webster Online Dictionary*; https://www.merriam-webster.com/dictionary). For instance, for the musical lyric phrase ‘*held your breath and the door*’ (Morissette & Ballard, 1995), the word *door* is surprising because when it is encountered it necessitates a different interpretation of *held* than the one activated when the first direct object ‘*your breath*’ is encountered. *Zeugma* refers to this kind of infelicity, humor, or strangeness that results from such conflicts. So-called zeugma tests, in which putative ambiguous words’ multiple potential meanings are referred to within the same sentence, have been argued to serve as diagnostics for detecting lexical ambiguity (see Cruse, 1986; Lewandowska-Tomaszczyk, 2007; Sennet, 2021; Tuggy, 1993). They are one among several alternative methods (e.g., contradiction, cross-linguistic and definitional tests) that have been proposed for this purpose, traditionally by philosophers, logicians, and theoretical linguists (see Viebahn, 2018), each with certain strengths and weaknesses. Some drawbacks to zeugmatic tests, for instance, are that they have been suggested to be better for detecting homonymy than for some kinds of polysemy (for discussion, see Moldovan, 2021; Viebahn, 2018) and have been argued to be context-inconsistent. For example, Sennet (2021) points out that while the sentence ‘*Judy’s dissertation is thought provoking and yellowed with age*’ is zeugmatic, the alternation ‘*Judy’s dissertation is still thought provoking although yellowed with age*’ is not. Although these judgments might be debated, it is important to point out that such proposed weaknesses of zeugma tests primarily come into play when categorical judgments are solicited. Under our proposed use of zeugma for similarity testing, we would not ask participants to make binary acceptability judgments, but rather provide ratings on a continuous scale of context-biased tokens’ meanings/similarities, which is more in line with the idea of a continuum of ambiguity. Indeed, Lewandowska-Tomaszczyk (2007) describes how examples like the *dissertation* sentences highlight the “fuzzy boundaries” between different types of lexical ambiguity and argue against rigid categorical lexical ambiguity distinctions.

As an alternative to more traditional methods of similarity norming, we also suggest that an added benefit to employing the zeugmatic manipulation for similarity norming is that the sense or degree of meaning mismatch may offer readers a more implicit cue about the degree to which the two meanings of the word are similar or different. In contrast to between-sentence or word pair designs, within-sentence infelicity arises in zeugmatic constructions because a second meaning of a word is obligatorily and unconsciously mapped to an alternative and already activated meaning of the same word. Participants still have to reach a judgment about meaning similarity, but their reaction to the zeugma may support this decision and possibly tap into different stages of word processing—an idea that will ultimately need to be put to the test experimentally.

### Method

#### Materials

We set out to assess meaning/sense similarity for 320 items, with equal numbers of items from each of the following four categories: homonyms, irregular polysemes, regular polysemes, and unambiguous words. See Appendix 2, Column A in supplementary material for the list of words. The ambiguous words, when possible, were drawn from the dominance-normed items in Study 1. While our pre-dominance norming categorization relied on dictionary criteria, our revised post-dominance norming sorting considered the top two meanings/senses associated with the responses provided in the dominance norming. For example, although the word *BAR* was originally categorized as a homonym based on separate dictionary entries (*rigid solid shape* vs. a *unit of pressure*), the dominance norming indicated that the two most frequent responses were related to polysemous senses of the first definition only (*where drinks are served* vs. *an amount of food served in a rectangular shape, e.g., a chocolate bar*). For this reason, BAR was considered as a polyseme for the next stage of norming. See Column D of Appendix 2 in supplementary material for ambiguous word categories.

In choosing ambiguous words from our dominance norming for the similarity norming, we prioritized selection of more meaning/sense-balanced items. As an initial criterion, we aimed to select items with dominance scores of ≤0.75, a cited value in the literature for differentiating ambiguous words with biased versus unbiased meanings (Armstrong et al., 2012). Again, under Armstrong’s dominance calculation, a dominance score of 0.0 represents a perfectly balanced item and a score of 1.0 represents a completely biased item. In addition, a second preference for inclusion in the similarity norming was for the ambiguous words’ most commonly provided meanings or senses to share the same part of speech. This was in part due to our experimental goals for subsequent studies, which would require construction of sentence stimuli around multiple meanings/senses of these words. This would be difficult if a word functioned as, for instance, a verb in one case but as a noun in another (e.g., *CLASH, DESIGN, FILM, LIMP*). We detail word selection for the individual ambiguity categories in the following paragraphs.

##### Unambiguous words

Including an unambiguous word category in our similarity norming was intended to provide a baseline for assessing meaning/sense similarity for the more ambiguous items, and to encourage participants to use the full range of the similarity rating scale. The criterion used for unambiguous word selection was a single dictionary entry with a single meaning listed (e.g., *COFFIN, EASEL, HEXAGON, NOON*).

##### Polysemes

From the list of words that were dominance normed, we categorized the polysemes (Column D of Appendix 2 in supplementary material) as either regular (aligning with the metonymic categorization) or irregular (aligning with metaphoric polysemes), according to whether or not they followed one of many generative polysemous relationships outlined in the literature (e.g., Apresjan, 1974; Barque & Chaumartin, 2009; Pustejovsky, 1998; see Table 1 for some examples of these relations). From the dominance-normed items, we were able to select 80 irregular polysemes that met our criteria (e.g., *STRAW, SPEAKER*). For the regular polysemes (e.g., *LIBRARY, ORANGE*), 70 items met our criteria with dominance scores ≤0.75, and an additional two had dominance scores of 0.76. Since our planned eventual experiments required 80 regular polysemes, we supplemented this list with eight additional items not included in our dominance norming (*CREAM, FLASK, LILAC, RECIPE, SILVER, TAN, TUNNEL, VASE*), but whose senses shared the same polysemic relationships (substance/color; container/contents; figure/ground; producer/product) as the items selected from the dominance norming. We presumed that their dominance values would lie within a similar range to those normed. Since there are few existing dominance norms for polysemes, we were unable to rely on norming values from other studies.

**Table 1:** Representative Regular Polysemic Sense Relations

Relation	Example
X represents Y	playing card represents person or entity (*QUEEN, KING*)
	container represents contents (*BOWL, GLASS, TEASPOON*)
	artifact represents activity (*SHOWER, BATH, BASEBALL*)
	building represents institution (*CHURCH, COURT, COLLEGE*)
	company represents product (STARBUCKS, KLEENEX)
X is caused by Y	fee is caused by action (*ADMISSION, ANCHORAGE*)
X is produced by Y	art from artist (*MOZART, PICASSO, HEMINGWAY*)
X produces Y	business firm produces publication (*NEWSPAPER, MAGAZINE*)
	device/broadcast (TELEVISION, RADIO)
X is from Y	flesh/meat/food from animal (*CHICKEN, EGG, LOBSTER*)
	product from plant (*OAK, COTTON*)
X is about Y	division responsible for work type (*EDUCATION, ENERGY, TRANSPORT*)
X accompanies Y	music that accompanies dance (*HIP HOP, POLKA, WALTZ*)
X covers Y	cloth covering that covers body part (*ELBOW, KNEE*)
X is included in Y	river passes by region (*MISSISSIPPI, AMAZON, MISSOURI*)
X is typical of Y	color typical of a substance (*EMERALD, LILAC, IVORY*)
	language spoken/food eaten by a person (*KOREAN, ITALIAN, FRENCH*)
X is part of Y	Object figure/ground or enclosure/opening (*CHIMNEY, WINDOW, GATE*)

##### Homonyms

We aimed to select 80 homonyms from the list of dominance-normed items whose top meanings were both relatively balanced and the same part of speech. Sixty-five homonyms met our part of speech criterion and had dominance ratings ranging from 0.0 to 0.79 (extending our cutoff slightly). To supplement this list, we included nine homonyms from Armstrong et al. (2012) whose dominance values were <0.75; to round out this list, we chose six additional homonyms from our dominance norming whose dominance values exceeded the 0.75 cutoff, but for which the dominance scores from Armstrong et al.’s (2012) study were ≤0.75. Refer to Appendix 2, Columns B and C in supplementary material, where these exceptions are noted.

##### Zeugma sentence construction

To test meaning/sense similarity for ambiguous words ranging from homonyms to irregular and regular polysemes, as well as unambiguous words, we began by writing two sentence contexts for each critical word. For the homonyms and polysemes, each of those two contexts constrained for one of the top two meanings or senses as determined through dominance norming. For example, for a homonym like *BARK*, the first context was ‘*The trees had a rough* *bark.*’, and the second was ‘*The poodle had a loud* *bark.*’, For unambiguous words, both contexts constrained for the same (the only) meaning; for example, for *pigeons*, the first context was ‘*Messages were carried by the* *pigeons.*’, and the second was ‘*A homeless man fed the* *pigeons*.’ Note that the two contexts were not intended to differ systematically in the degree to which they disambiguated the target word; in other words, each disambiguating clause was intended to be equally informative about the meaning it selected.^6^ The two contexts were then combined into a single sentence by use of a conjunction (*and* for 315 items, and *but* or *or* for the remaining five). For the majority of resultant sentences, the second instance of the critical word was replaced by an anaphoric referring expression: for 8 items, the critical word was repeated because an anaphoric referring expression was not appropriate due to issues relating to animacy or part of speech (e.g., JAPANESE food vs. people). Although commas would have been grammatically correct, they were omitted at the conjunction to enhance potential zeugmatic effects. See Table 2 and Column E of Appendix 2 in supplementary material for zeugmatic stimulus sentence examples from the similarity norming.

**Table 2:** Similarity Norming Examples of Zeugmatic Sentence Stimuli

Similarity norming sentence	Ambiguity category	Item mean similarity rating
*Mildew spray removed the* ***MOLD*** *and concrete was poured into* ***one.***	Homonym	1.69 (*SD* = 1.28, *SEM* = 0.14)
*The stereo system was missing a* ***SPEAKER*** *and the conference organizers scheduled* ***one****.*	Irregular polyseme	2.38 (*SD* = 1.35, *SEM* = 0.14)
*The paint she picked was* ***TAN*** *and in the summer she always gets* ***one****.*	Regular polyseme	3.63 (*SD* = 1.56, *SEM* = 0.17)
*The story was about a* ***MERMAID*** *and the girl swam like* ***one****.*	Unambiguous	6.41 (*SD* = 0.92, *SEM* = 0.10)

#### Participants

Given our novel approach to similarity norming, we elected for a larger sample size than previous ambiguous word similarity/relatedness normings described in the literature, where the number of similarity ratings collected per item has often not exceeded *N* = 30 (e.g., Brocher et al., 2018; Durkin & Manning, 1989; Klepousniotou et al., 2008; Rodd et al., 2002); thus, 98 native English-speaking UCSD undergraduates (74 females, 22 males, two nonbinary) between the ages of 18 and 25 years took part in the online similarity norming study for course credit. Participants were recruited from the UCSD Psychology Department subject pool’s online scheduling system. Informed consent was obtained from all participants after the norming procedure had been outlined for them.

#### Norming procedure

Similarity norming was administered through an online survey, designed using Qualtrix software, with each participant norming the same 320 sentences. Stimuli were presented with unique, randomized orderings for each participant. The ambiguous (or unambiguous) critical word of each sentence was presented in an uppercase black font and the anaphoric referring expression in a red font color. In terms of assessing meaning similarity, criteria to guide decision-making were taken from McRae et al. (2005), including whether the tokens share features, have similar properties, or whether they occur in similar contexts. Participants were instructed as follows:

*You will read a number of sentences. Each has a word highlighted in black and another in red. The black words are mostly nouns and the red words (mostly pronouns) refer to the black word. For each sentence, please judge the similarity in meaning between the black word and what’s being referred to in red. Meaning similarity should be ranked on a scale ranging from 1 (“meanings not similar at all”) to 7 (“the very same meaning”).*

*As a guide to judging similarity of meaning, consider the following questions:*

- *Can the two meanings appear in similar contexts?*
- *Do the two meanings share physical or functional properties?*
- *Do the two meanings taste, smell, sound or feel similarly?*
- *Do the two meanings behave similarly?*

These instructions were followed by several example sentences with critical words that spanned the ambiguity continuum. Possible explanations for why the critical words and their anaphoric referring expressions might be rated as more or less similar were also provided to participants.

Using the zeugma test for similarity norming offered a way of biasing words’ different meanings or senses within a single sentence so that individuals could respond fairly intuitively to the mismatch or correspondence of the different meanings or senses, without having to draw comparisons across sentences. This design was also appropriate across the different types of ambiguous as well as unambiguous words, allowing for graded responses to meaning similarity that went beyond categorical classifications that stemmed from historical associations, which may or may not have been obvious to comprehenders.

#### Data preprocessing/rejections

Data from 10 participants were dropped (leaving 88 remaining participants) based on one or more of the following reasons: (1) incomplete or uncooperative responses, (2) variance of ≤0.5 between condition means for the Homonym and Unambiguous conditions (i.e., those with the anticipated most and least similar meanings, therefore intended to identify participants who gave similar ratings regardless of item type), or (3) apparent lack of participant understanding of instructions. In addition, two of the total 320 sentences were excluded from analysis: (1) one item (*CORN*), although reclassified by the experimenters as a polyseme following the dominance norming, was inadvertently tested in a sentence based on the homonym meanings, and (2) there was an experimenter error in designing the zeugma stimulus sentence for another homonym (*POACHED*). This left 78 items in the homonym condition and 80 items in each of the other three conditions. For each of these 318 items, there were 87–88 responses. The individual participant and item similarity ratings from Study 2 may be accessed at https://osf.io/g7fmv/.

### Analysis and results

The similarity rating means, standard deviations, and standard errors for the individual items are shown in Appendix 2, Columns F, G, and H in supplementary material.

#### Inter-annotator agreement

To assess the reliability of the ratings from our similarity norming, we calculated inter-annotator agreement using a *leave-one-out* method. For each participant, we calculated the Spearman’s rank correlation between that participant’s set of similarity ratings (i.e., for all items that a given participant observed), and the mean rating assigned to those items by the 87 remaining participants. This yields a score indicating how much each participant’s judgments were aligned with the judgments made by other participants. Similar approaches have been used in past work (e.g., Trott & Bergen, 2021); crucially, this score can be compared to the average correlation obtained using language models (see description of computational analysis of human similarity norms below), providing an *upper bound* of how much agreement can be expected.

**Fig. 2:**
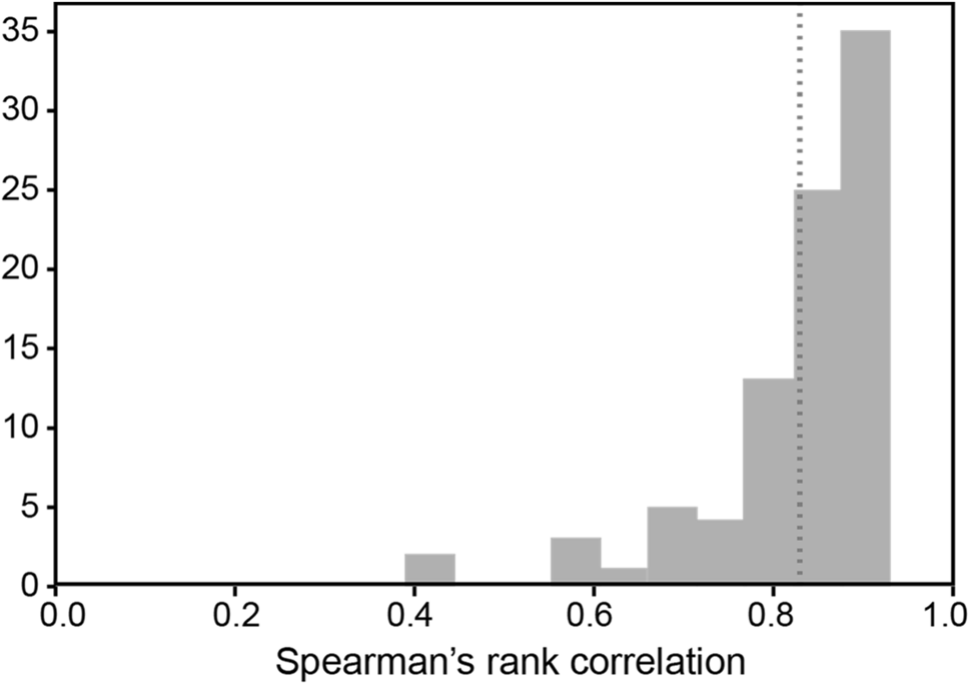
Inter-annotator agreement for similarity norming, plotting the distribution of Spearman’s rank correlation scores, calculated using a leave-one-annotator-out scheme. The dotted line represents the mean inter-annotator agreement (0.83)

Across the 88 participants, the mean correlation was 0.83 (*SD* = 0.1, median = 0.86). This is comparable to—if slightly higher than––other work investigating inter-annotator agreement for similarity norms (Hill et al., 2015; Trott & Bergen, 2021). The scores ranged from a minimum of 0.39 to a maximum of 0.93. However, as depicted in Figure 2, only two scores fell below 0.5, and the majority (75%) of scores were above 0.8.

#### Descriptive statistics of similarity ratings

**Fig. 3:**
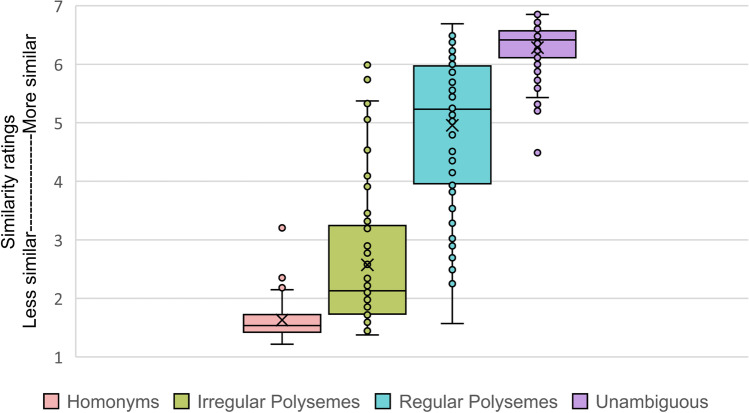
Item mean similarity ratings (318 items, 88 participants), box-and-whisker plots within ambiguity conditions, with X indicating mean condition values

We begin with some descriptive statistics for the similarity norming data set. Similarity rating condition means with standard deviations are as follows: Homonyms (*M* = 1.63, *SD* = 0.33), Irregular Polysemes (*M* = 2.57, *SD* = 1.14), Regular Polysemes (*M* = 4.96, *SD* = 1.27), and Unambiguous (*M* = 6.28, *SD* = 0.44). Mean similarity ratings of items within the four ambiguity conditions are also displayed in a box-and-whisker plot in Fig. 3. These plots suggest that, on average, there was an expected range of similarity values between the ambiguity extremes, with homonyms exhibiting the most dissimilar meanings and unambiguous words the most similar. Values for the polysemes fell in between, with the irregular polysemes notably showing, on average, more distinct meanings (more closely resembling homonyms) and the regular polysemes showing more similar meanings (closer to unambiguous words).

Post hoc pairwise statistical comparisons (*t* tests with Bonferroni correction for multiple comparisons) indicated that the similarity ratings for each condition differed significantly (*p* < .01) from those of each of the other conditions. These data are consistent with proposals of a continuum of ambiguity across the four conditions, within both theoretical linguistics (e.g., Apresjan, 1974) and experimental work (e.g., Klepousniotou et al., 2012). Figure 3 and the standard deviations of the conditions illustrate that both polyseme categories exhibited within-category variability greater than either the homonyms or unambiguous words. However, across all four categories, there also appeared to be a good deal of overlap in similarity values; for example, some words classified as regular polysemes were rated as having meanings equally similar to those of many unambiguous words. Both patterns suggest potential other factors (i.e., beyond dictionary-based ambiguous word categories) that may be contributing to the variance in similarity ratings, both within and across conditions. One intriguing candidate, for instance, is the rated concreteness of ambiguous words, with Gilhooly and Logie (1980a) noting that abstract words tend to be more ambiguous than concrete ones. Aligning with this idea, a lookup of concreteness ratings (consulting norms from Brysbaert et al., 2014, using a scale of 1 to 5, with 5 being most concrete and 1 most abstract) for our tested regular and irregular polysemes indicates categorical mean concreteness differences, with regular polysemes (having more similar meanings) being on average more concrete than irregular polysemes (mean concreteness ratings being 4.7 and 3.7, respectively). This raises interesting questions relating to the perhaps more inherent difficulty of rating the similarity of more abstract versus more concrete word meanings, but also about what it means to rate the concreteness of a word with multiple meanings. Although intriguing, further exploration is a matter for future research and is beyond the scope of the current paper.

Qualitatively, these descriptive findings are broadly consistent with the predictions of the “cues to meaning” framework (mentioned in the Introduction), in which word meanings are characterized as occupying a continuous, context-sensitive landscape (Elman, 2004; Li & Joanisse, 2021). It should be noted, however, that this study was not conducted with the explicit goal of confirming or disconfirming the cues to meaning framework (nor was a statistical test run to do so); rather, the norms collected here could serve as a crucial first step to testing that framework in future work.

#### Computational analysis of human similarity norms

Given the results of the Study 2 similarity norming, we determined that conducting an additional computational analysis would allow for the evaluation of factors that may help explain variance beyond that accounted for by our dictionary-criteria ambiguity categories (Homonyms, Irregular Polysemes, Regular Polysemes and Unambiguous). Doing so involved comparing the human meaning similarity judgments to BERT (Devlin et al., 2018), a neural language model (NLM) trained on a large corpus of (mostly written) English text.

NLMs such as BERT are well suited to operationalizing the cues to meaning framework described earlier. Indeed, a recurrent neural network––a precursor to models like BERT––was an inspiration for this framework (Elman, 2004), and several recent studies have used NLMs as computational models of human sense similarity (Haber & Poesio, 2020; Li & Joanisse, 2021; Nair et al., 2020; Trott & Bergen, 2021). Rather than representing word meanings as discrete entries in a mental dictionary, BERT’s “representation” of a word corresponds to the distribution of hidden unit activations in each layer of the network. These representations reflect information about a word’s distributional profile and its relationship to the immediate sentential context––necessarily, then, they encode both semantic and syntactic information^7^, though there is some evidence that earlier layers of BERT encode syntactic information, while later layers encode semantic information (Tenney et al., 2019). Importantly, this activation profile is both continuous (i.e., it situates a word at some location in vector-space), and context-sensitive (i.e., the exact state elicited by a given wordform is dependent on the immediate context). This allows for extraction of continuous measures of meaning similarity (as measured by BERT), which can be compared to human ratings.

Of course, BERT represents a particular operationalization of the cues to meaning framework: It is trained on linguistic input alone, and it has a particular network architecture. This means that BERT has no access to grounded information about word meaning (Bender & Koller, 2020). This potential limitation, however, makes BERT suitable for asking how much information about a word’s meaning can be gleaned solely from statistical regularities in that word’s pattern of use. The distributional hypothesis states that words with more similar meanings should appear in more similar contexts (Firth, 1957; Harris, 1954; Sahlgren, 2008). If so, then word meaning should be derivable, at least in part, from the contexts in which the word occurs. On some accounts, distributional statistics play a significant role in informing human lexical knowledge (Andrews et al., 2009; Lupyan & Lewis, 2019; Lupyan & Winter, 2018), and there is psycholinguistic evidence that humans can infer a novel word’s meaning from its distributional similarity to other words (Ouyang et al., 2017). Further, computational models that exploit distributional statistics––sometimes called distributional semantic models––such as Latent Semantic Analysis (Landauer & Dumais, 1997) and *word2vec* (Mikolov et al., 2013), have proven relatively successful in predicting human judgments of similarity and relatedness (McDonald & Ramscar, 2001; Mikolov et al., 2013), particularly for decontextualized (which we have referred to in this paper as ‘isolated’) words (e.g., *dolphin* vs. *shark*). More recent models like BERT (Devlin et al., 2018) produce “contextualized embeddings”, which reflect not only a word’s overall pattern of use but also its immediate sentential context; in principle, this allows differentiation between distinct senses of an ambiguous word. The extent to which these models can predict human similarity judgments can be seen as a proxy for how much human semantic knowledge can be derived from distributional statistics alone, and how much might require other, perhaps extra-linguistic information (Andrews et al., 2009). This also serves an applied goal: if NLMs like BERT fail to capture crucial semantic distinctions, then there may be room for improvement, such as augmenting the sources of information they are exposed to during training (Bender & Koller, 2020).

There are a number of distinct pretrained NLMs to choose from, all of which are trained on linguistic input alone and provide continuous meaning representations. We selected BERT because of its state-of-the-art performance on word sense disambiguation tasks (Loureiro et al., 2020), as well as the extensive body of literature probing where and what kind of information is encoded in BERT (Rogers et al., 2020; Tenney et al., 2019). Future work could extend these analyses to other models, such as ELMo (Peters et al., 2018) or XLNet (Yang et al., 2019).

Like other statistical language models, BERT is a neural network trained on a large corpus (>4B word tokens) of mostly written text, using either masked language modeling (MLM) or next sentence prediction. In MLM, a given word token is “masked” from a sentence (e.g., “the cup [MASK]”), and BERT must predict the identity of that masked word; over many iterations, BERT tunes its representations of each word and surrounding tokens to improve its predictions, eventually allowing it to be used to categorize part-of-speech, semantic roles, word senses, and more (Tenney et al., 2019). In addition to being beneficial for training, MLM can be used “online” to obtain the probability BERT assigns to a given word token that it would appear in a given context.

We obtained two different (but related) measurements from BERT. First, for each zeugmatic sentence (e.g., *The player swung a bat and the vampire was bit by one*.), we calculated the cosine distance between BERT’s contextualized embeddings for the target word (e.g., *bat*) and the anaphoric referring expression (e.g., *one*). Cosine distance is often used to model human similarity or relatedness judgments, both for words appearing in isolation (Hill et al., 2015) and in context (Haber & Poesio, 2020; Trott & Bergen, 2021). Each contextualized embedding corresponds to BERT’s representation of a given word token and is thought to reflect syntactic *and* semantic factors (Tenney et al., 2019). Larger cosine distances correspond to more dissimilar vectors, and thus should correlate with *lower* similarity ratings (i.e., less similar meanings); conversely, smaller cosine distances should correspond with higher similarity ratings––with a cosine distance of zero reflecting that a vector is being compared with itself. Because different layers of BERT are thought to encode different information (Tenney et al., 2019), we calculated cosine distance between the contextualized representations obtained for all 12 layers of BERT (base).

Second, using masked language modeling, we calculated the surprisal (i.e., the negative log probability), for each anaphoric referring expression (e.g., “one”). Past work has used surprisal to predict a number of “online” measures of language processing, such as reading time (Goodkind & Bicknell, 2018) and the N400 effect (Frank et al., 2015; Michaelov & Bergen, 2020). Like cosine distance, surprisal likely reflects syntactic as well as semantic features of a word. Unlike cosine distance, surprisal captures the unexpectedness of a *particular* word token appearing in a particular context. For zeugmatic sentences, this can be viewed as a proxy for how “surprised” BERT would be that two meanings of an ambiguous word are coreferenced (and copredicated). Thus, we predicted that surprisal should correlate both with condition (e.g., Homonymous vs. Unambiguous) and human similarity ratings; that is, more similar meanings should be more likely to be coreferenced, while less similar meanings should be less likely to be coreferenced. In our use, both cosine distance and surprisal were intended to measure how similarly BERT views two meanings of an ambiguous word––where “similar” likely incorporates both semantic and syntactic features of a word’s distributional profile and immediate sentential context.

##### Materials

There were 318 sentences for which we obtained human similarity ratings. Of these, we excluded four sentences in which the anaphoric expression contained multiple words (e.g., “a lot”). Thus, we considered 314 sentences (and their similarity ratings) in the final analysis.

##### Procedure

We calculated two measures: (1) the cosine distance between the target word and the anaphoric expression, and (2) the surprisal of the anaphoric expression.

To calculate cosine distance, we ran each sentence through the pretrained BERT-base (uncased) model using the HuggingFace transformers library^8^ in Python (Wolf et al., 2020). BERT-base has 12 layers, each with 768 hidden units, and 12 attention heads. We obtained the vector representations for the target word (e.g., “bat”) and the anaphor (e.g., “one”) in each layer, then calculated the Cosine Distance between those vectors. Note that BERT uses a WordPiece tokenizer (Wu et al., 2016), which splits words into either the full form (i.e., one word corresponds to one token) or into multiple components (e.g., the word “surfing” might be decomposed into “surf” and “##ing”). Each individual *token* receives its own embedding. In cases where a given word was decomposed into multiple WordPiece tokens, we computed the average of the corresponding WordPiece embeddings (Wu et al., 2016). There were 38 sentences for which this was necessary (representing about 12% of the 314 sentences in the final dataset).

To calculate Surprisal, we used the pretrained BertForMaskedLM model object (BERT-base, uncased), from the HuggingFace library. For each sentence, we masked the anaphoric expression (e.g., “one”) with the “[MASK]” token, and then used BERT to calculate the probability of the given anaphor occurring in that slot. We calculated surprisal by taking the negative logarithm of this probability.

##### Analysis and results

We had two primary questions. First, do the measures obtained (cosine distance and surprisal) correspond to the categorical condition variable (e.g., Homonym vs. Unambiguous)? Second, do the measures obtained predict human judgments of similarity––above and beyond condition? We approached both questions using nested model comparisons in R (R Core Team, 2020). Specifically, in each case, we compared a full model including the predictor of interest (e.g., surprisal) to a model omitting only that predictor, and conducted a log-likelihood ratio test to determine whether the full model explained significantly more variance. Models were built using the *lme4* package (Bates et al., 2015). For each question, the reported *p* values were adjusted for multiple comparisons using Bonferroni corrections to account for the fact that two separate BERT-derived metrics (cosine distance and surprisal) were used to test equivalent hypotheses.

**Fig. 4:**
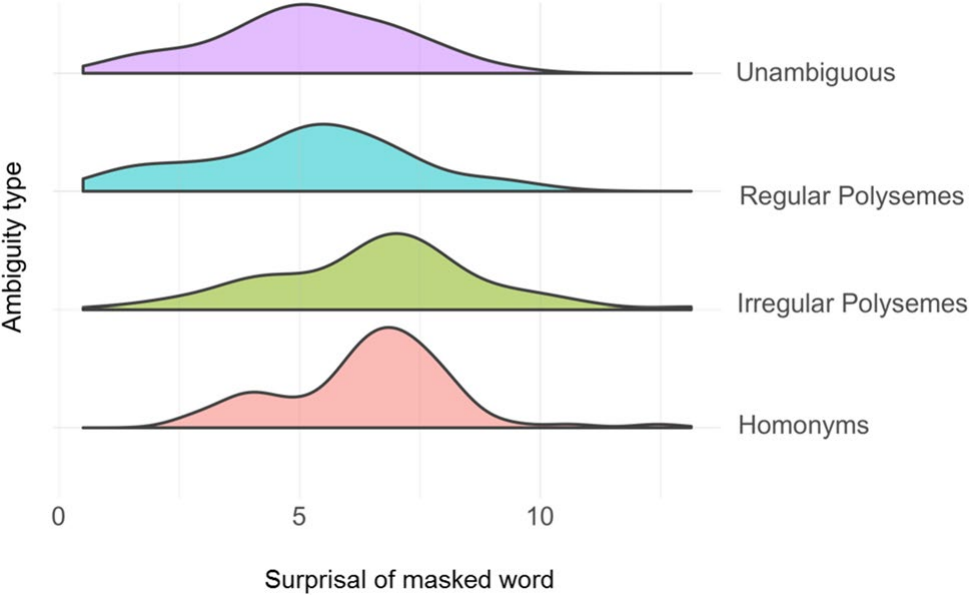
Distribution of surprisal values by condition. The surprisal of the anaphoric expression tended to be higher for homonymous and irregular polysemy items than unambiguous or regular polysemy items

First, we built a full model with Surprisal as the dependent variable, a fixed effect of condition, and random intercepts for Anaphor (i.e., the specific anaphoric expression that was used for that sentence) and compared this model to a model omitting only condition. We also performed an analogous analysis using cosine distance as the dependent variable. In both cases, the condition variable contained four levels: Homonymy, Irregular Polysemy, Regular Polysemy, and Unambiguous. Thus, these analyses asked about whether there was variance *overall* in the dependent variable (cosine distance or surprisal) that was related to the different levels of the condition variable. We relied on R’s default coding scheme, which automatically selected the mean of the alphabetically-first level as the Intercept, which in this case was Homonymy. After correcting for multiple comparisons, the improvement in model fit when predicting Surprisal was no longer statistically significant, χ^2^(3) = 8.94, *p* = .06, nor was the improvement in model fit when predicting cosine distance, χ^2^(3) = 7.47, *p* = .11. The distribution of surprisal values by condition is displayed in Fig. 4. The results of both statistical analyses suggest that any potential relationship between these BERT-derived metrics and ambiguity type was weak at best.

Second, we built a full model with mean similarity as the dependent variable, fixed effects of both condition and surprisal, and random intercepts for anaphor. This full model explained significantly more variance than a model omitting only the effect of condition, χ^2^(1) = 12.82*, p =* .0006, indicating that variation in the Surprisal of the anaphoric expression––as measured by BERT––captured variance in participants’ similarity judgments, above and beyond the categorical variable of condition. One explanation for this result is that both polysemy conditions spanned a large spectrum of similarity; for example, similarity ratings for items in the Regular Polyseme condition ranged from 1.57 to 6.69 (see Fig. 3). Thus, although similarity ratings tended to covary with condition, there remained a substantial amount of variance to be explained in participants’ similarity judgments for polysemous items––and crucially, surprisal explained some of this variance. We also conducted an identical analysis with Cosine Distance (from the final layer) instead of Surprisal. In this case, the improvement in model fit was no longer significant after correcting for multiple comparisons, χ^2^(1) = 2.56, *p* = .21. Finally, we asked explicitly whether Surprisal explained variance in Similarity above and beyond cosine distance. A model with fixed effects of both surprisal and cosine distance exhibited better fit than a model containing only cosine distance, χ^2^(1) = 13.74, *p* = .0004; in contrast, the improvement of the full model compared to a model containing only Surprisal was statistically non-significant after correcting for multiple comparisons (*p* = .19).

**Fig. 5:**
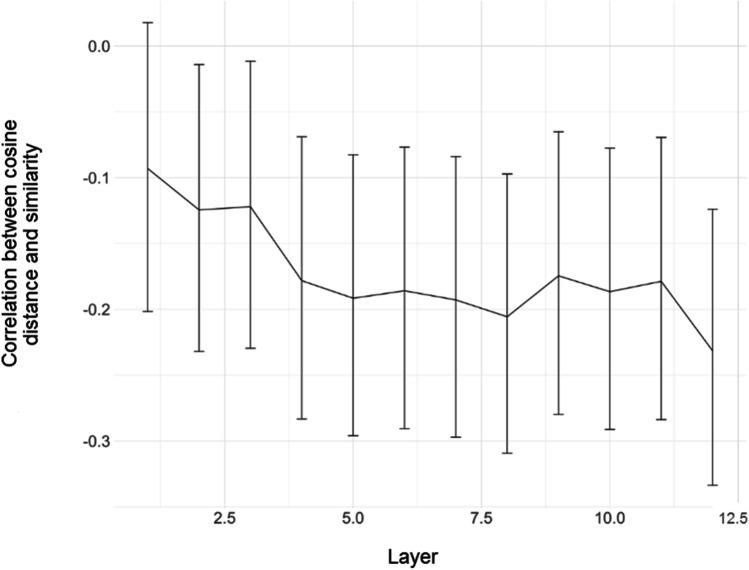
Correlation between cosine distance (i.e., between the vectors for the target word and the anaphoric expression) and human similarity ratings for each successive layer of BERT-base. Vertical bars represent the 95% confidence interval for values of *r* (calculated using the *cor.test* function in R)

Because earlier work (Tenney et al., 2019) suggests that different layers of BERT encode different kinds of information, we also conducted an exploratory analysis asking which layers of BERT exhibited the strongest correlation between cosine distance and similarity. The strongest (most negative) correlation was obtained in the final layer (layer 12) of BERT-base (*r* = −.23). As depicted in Fig. 5, however, the correlation did not change considerably beyond the fourth layer. Importantly, even the strongest correlation was much lower (by a factor of 4×) than the average inter-annotator agreement (*r* = .83). Additionally, the correlation between mean similarity and surprisal (*r* = −.35) was less than half the average inter-annotator agreement. Both findings indicate that there is considerable room for improvement to match the human benchmark.

#### Conclusions

With decades of psycholinguistic ambiguity research having relied heavily upon experimental manipulations involving homonyms, another class of ambiguous words—polysemes, which are more frequent in natural language but much less tested—also offers a window into how lexical meanings are stored and processed. In experimental comparisons to date, the majority of studies have concluded that polysemes (with their significant sense overlap) exhibit underspecified meaning interpretation, and unlike homonyms, their interpretation has rarely been shown to be influenced by sense frequency or context (e.g., Frazier & Rayner, 1990; Klepousniotou, 2002; for reviews, see Eddington & Tokowicz, 2015; Frisson, 2009). Such findings have implied that the two ambiguous word types are differentially processed, with only a small number of studies arguing for rapid polyseme sense selection and meaning representations more akin to those proposed for homonyms (e.g., Foraker & Murphy, 2012; Klein & Murphy, 2001). With some of this debate hinging on the very nature of the specific experimental materials utilized, and in particular on the composition of the polysemic stimuli, it is crucial to establish the sense/meaning relations of a wide range of ambiguous word types.

A clear finding from the current similarity norming is that polysemes do not constitute a unitary category—not only in linguistic theory but in how contemporary language users interpret their multiple semantic senses. Recalling the definition of polysemes outlined earlier in this paper, a defining characteristic is that the different senses have a shared semantic origin. The current results indicate that—on average—even with a common etymology, contemporary language users recognize varying degrees of sense similarity and semantic overlap across regular and irregular polysemes. This difference between polyseme types was on par with the categorical differences observed between the meanings of homonyms and irregular polysemes, as well as between regular polysemes and unambiguous words. The polyseme difference may be particularly relevant because relatively few published experimental reports examining online ambiguity processing have distinguished between the two types. An advisable approach for future studies would be, at a minimum, to consider the different types of polysemes, the nature of the polysemic relations, and the degree of semantic sense similarity. Moreover, although our findings indicated that on average there were significant differences in similarity ratings between the different ambiguous word types, the fuzzy borders and overlap between all of the categories we have examined suggest that an even better approach might be to consider that words lie along a continuum of ambiguity (meaning/sense similarity). This working assumption may ultimately prove more useful for guiding research about how words’ multiple meanings or senses are neurally activated and functionally organized in the brain. In turn, future work could use the norms reported here to investigate the cues to meaning framework (Elman, 2004).

In addition, our computational analyses of the similarity ratings assessed using zeugmatic sentences indicate that although dictionary-based ambiguous word categories tend to be predictive of human meaning similarity judgments, there is substantial variance unaccounted for by these classifications—some of which seems to be explicable by the degree of Surprisal of the anaphoric expressions. This finding, along with several other recent studies (Haber & Poesio, 2020; Li & Joanisse, 2021; Nair et al., 2020; Trott & Bergen, 2021), suggests that neural language models like BERT might be useful tools for modeling human sense knowledge––particularly for theoretical frameworks that view meaning as occupying a continuous, context-sensitive landscape (Elman, 2004). Second, because BERT is trained on linguistic input alone, its ability to explain variance in human similarity ratings indicates that some degree of lexical semantic knowledge can in principle be derived from distributional regularities. This serves as partial validation of the distributional hypothesis (Firth, 1957; Harris, 1954).

On the other hand, the correlation between Surprisal and human similarity ratings fell far short of human inter-annotator agreement (by a factor of over 2x) and was also lower than the correlation reported in similar studies (Nair et al., 2020; Trott & Bergen, 2021). The contrast with previous studies could be explained by the fact that the linguistic stimuli in this task involved anaphora (e.g., “one”), thereby requiring a form of coreference resolution; this may be more difficult for BERT than tasks that involved a repetition of the same word form in different sentential contexts (Nair et al., 2020; Trott & Bergen, 2021). Importantly, the gap between human and model performance reveals room for improvement: clearly, human participants were drawing on semantic knowledge that was either unavailable to or unrepresented by BERT. It also demonstrates the utility of this lexical resource: a gap in performance offers space for theoretical explanations as well as engineering improvements. Future psycholinguistic work could seek to disentangle the factors responsible for explaining human similarity judgments––beyond dictionary-based categories of ambiguity, and beyond the “distributional baseline” provided by BERT. In turn, NLP practitioners could ask about the extent to which introducing different/various sources of information (e.g., multimodal grounding) into BERT’s training regime would improve its ability to approximate human judgments, and if so, figure out how to attain and integrate this information. Additionally, practitioners could investigate how well other BERT-derived metrics predict human similarity ratings. For example, previous work (Clark et al., 2019) found that the behavior of BERT’s attention heads encoded information about syntactic relations. In the current case, syntactic information from the attention heads could be used to assist in coreference resolution (e.g., identifying multiple mentions of the same entity).

## Supplementary Information

ESM 1 (DOCX 114 kb)

ESM 2 (DOCX 263 kb)

ESM 3 (DOCX 82.4 kb)

## Data Availability

Materials and summarized information for individual items from these dominance and similarity norming studies are included as Appendices in the Supplementary Information. The individual subject/item norming data collected and analyzed for the current studies are available in the OSF repository (https://osf.io/g7fmv/). These studies were not preregistered.

## Footnotes

1 The literature uses various terms, including *balanced*/*unbalanced*, *biased*/*unbiased*, *meaning frequency*, *polarization*, *dominance*, and *relative frequency* to refer to roughly similar ideas: the relative usage proportion of a word’s multiple meanings or senses to each other.

2 Because of the gender imbalance of participants in both norming studies, we conducted an analysis outlined in Supplementary Analysis 1 that demonstrates negligible differences between the responses of female and male participants.

3 During the time that our paper was submitted to the current journal and under review, Gilbert and Rodd (2022) published a discussion of how the number of observations needed to estimate the “true dominance” for an ambiguous word meaning is greater for words with more equibiased meanings (like the current materials) compared to words with more polarized (dominant and subordinate) meanings. Our sample size is in the recommended range. When N = 100 (a number approximating the average number of responses per item in our Study 1), true dominance of 0.5 is associated with a margin of error of ~10%.

4 Of the 558 dominance normed words, 410 items had responses that aligned with only two definitions/senses, 129 items aligned with three meanings, 16 items with four meanings, and three items with five meanings.

5 Of the 547 ambiguous words, 476 items had 90–100% of norming responses accounted for by the two most frequent meanings/senses; 41 items had 80–90%; 20 items had 70–80%; and 10 items had 56–70%.

6 See Supplementary Analysis 2 for empirical evidence that the informativity of the disambiguating clause did not systematically differ across the first or second context, or across conditions.

7 It is unlikely that the representations encode phonological information, except to the extent that a word’s orthographic or phonological profile is correlated with its textual distribution.

8 https://huggingface.co/transformers/index.html

## Code availability

Code to reproduce the primary analyses is available on GitHub: https://github.com/seantrott/zeugma_norms.
