# Supplementary material for: Offline dominance and zeugmatic similarity normings of variably ambiguous words assessed against a neural language model (BERT)
Source: Behav Res Methods. 2022 Jun 10;55(4):1537–57. doi: 10.3758/s13428-022-01869-6 (PMC10040203; doi:10.3758/s13428-022-01869-6)
Supplement: Supplementary file 1 — (DOCX 114 kb) [file 13428_2022_1869_MOESM1_ESM.docx]

**SUPPLEMENTARY ANALYSIS 1**

Both norming studies described in the primary manuscript were fairly unbalanced in terms of gender: at least 75% of the participants in both Study 1 (80 female, 17 male) and Study 2 (74 female, 22 male, 2 non-binary) identified as female. One concern is whether this would bias the item-level scores we report––i.e., the dominance scores for each word (Study 1), or the similarity ratings for the meanings in each zeugmatic sentence (Study 2).

To determine whether this concern was valid, we asked whether there were systematic differences in either the dominance scores or zeugmatic similarity ratings as a function of participant gender. We did this by repeatedly sampling a subset of female participants, recalculating the item-level scores using only that subset (i.e., either the dominance scores or similarity ratings), and then computing the correlation between those scores and the item-level scores calculated using only either the participants identifying as male or non-binary. In each case, the number of sub-sampled female participants was matched to the number of either male or non-binary participants in the comparison group (i.e., 22 male and 2 non-binary in Study 2, respectively).

The results are summarized in more detail below. Briefly, the analysis of Study 1 data suggests that while there is non-trivial variability across participants in dominance, this variability is not systematically correlated with participant gender. The analysis of Study 2 data indicates very high agreement across male and female sub-samples (mean *r* = 0.95). In both cases, the results suggest that the predominance of self-identified female participants is not necessarily a concern for the validity of the results.

**Dominance norming (Study 1) gender analysis results**

Study 1 had 80 participants self-identifying as female and 17 participants self-identifying as male. We repeatedly (1000x) sub-sampled from the set of 80 female participants, with each sample matched to the number of male participants. For each sample, we calculated the dominance scores for each word using the procedure described in the primary manuscript––i.e., we compared the probability of producing associations for the most frequent vs. the second-most-frequent sense and divided by the probability of the most frequent sense. We then compared these dominance scores to the set of scores obtained using only the 17 male participants.

The mean correlation coefficient across all samples was 0.52 (*SD* = 0.04, median = 0.52), and ranged from 0.41 to 0.63. This is neither very high nor very low; thus, to obtain a measure of how much correlation one would expect *within* each group––i.e., simply as a function of inter-participant variability and sampling error––we followed the same procedure for female participants only. In other words, we compared dominance scores using two distinct subsets of the female participants, again sub-sampling to match the size of the male sample (i.e., 17 participants). In this case, the mean correlation coefficient was 0.49 (*SD* = 0.04, median = 0.49), and ranged from 0.36 to 0.6. This is quite similar (and actually slightly lower) than the mean correlation coefficient when comparing male vs. female participants. This suggests that while there is non-trivial inter-participant variability in dominance ratings, this variability is not systematically correlated with self-reported gender.

**Similarity norming (Study 2) gender analysis results**

We repeatedly sub-sampled from the set of 74 self-identifying female participants, matched in size for either the number of self-identifying male participants (22) or self-identifying non-binary participants (2). In each case, we took 1000 samples. For each sample, we calculated the mean Similarity Rating for each item using responses from that sample. Finally, we calculated Pearson’s correlation coefficient between those mean Similarity Ratings and the Similarity Ratings obtained using only the male or non-binary participants.

The distribution of correlation coefficients between sub-sampled female participants and the 17 male participants is depicted in Supplementary Figure 1. As this figure illustrates, the correlation in each sample was very high (*M* = 0.95), with very little variance (*SD* = 0.004). This correlation is close to perfect (*r =* 1), indicating that any differences between male and female scores are likely due to sampling error––i.e., that there is no systematic difference in response bias between the groups. This suggests that the predominance of female participants in the sample need not be a concern, in terms of the item-level averages we report.


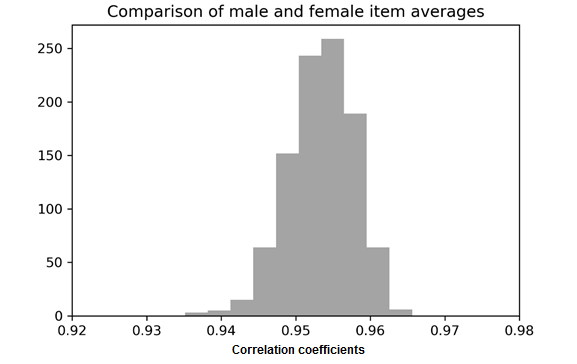


**Supplementary Figure 1.** Distribution of correlation coefficients between Study 2 sub-sampled female participants and 17 male participants.

Similar results were obtained when comparing sub-sampled female item-level averages to the scores from the two non-binary participants. The mean correlation was slightly lower (*M* = 0.87, *SD* = 0.04), but still quite high. Note that higher variance might be expected in this analysis, simply because the sample sizes are much smaller (two participants in each group). For comparison, recall that the mean inter-annotator agreement was 0.83 (*SD* = 0.1).

**SUPPLEMENTARY ANALYSIS 2**

The experimental stimuli for Study 2 consisted of a series of zeugmatic sentences. Each sentence (except for the Unambiguous sentences) made reference to two distinct meanings of an ambiguous word form. For example, in the sentence “Mildew spray removed the **mold** and concrete was poured into **one**”, the first clause evokes the fungal sense of “mold”, while the second clause evokes the container sense. Because the sentences were not counterbalanced, one potential concern is whether the two meanings within a sentence are not equally contextually discriminable. Although some minor variability in contextual discriminability is expected, it would be problematic if this variability were systematic in some way, e.g., if the first clause is always more disambiguating than the second, or if the difference in contextual discriminability varies across conditions.

To address this concern, we asked whether the Surprisal of the target word varied across contexts. Surprisal is a measure of the unexpectedness of observing a particular token in a particular context. In Study 2, we found that the Surprisal of the anaphoric expression was a better predictor of human Similarity judgments than the Cosine Distance across contexts. Here, we used Surprisal for a slightly different purpose––we wanted to know whether the disambiguating contexts in our stimuli exhibited systematic variation in how much they constrained the target word. If the Surprisal of “mold” is lower in “Mildew spray removed the **mold**” than in “Concrete was poured into the **mold**”, it suggests that the first context is more constraining than the second.

**Materials.** The original stimuli were single sentences, with the second meaning of the ambiguous word form always instantiated by an anaphoric expression (e.g., “one”). These sentences were not suitable for our current analysis, because the target word is not controlled across contexts (e.g., the Surprisal of “one” might always be lower than the Surprisal of the first word, simply because “one” is highly frequent). Thus, we split each zeugmatic sentence into two complete sentences, corresponding to the two meanings of the target word form. Then, we ran each sentence through BERT (Devlin et al, 2019) with the target word masked (e.g., “Mildew spray removed the [MASK]”), and calculated the Surprisal (i.e., the negative log probability) of the target word form (e.g., “mold”) appearing in the masked slot.

**Analysis.** We carried out two analyses. First, we asked whether the Surprisal of the target word form varied systematically across contexts. To test this, we built a linear mixed effects model with Surprisal as the dependent variable, a fixed effect of Order (First vs. Second), and a random intercept for word (each word had two observations). We compared this model to a model omitting only the effect of Order. The full model did not explain significantly more variance (*p* > 0.3*)*, suggesting that Surprisal did not co-vary systematically with the order of the contextually disambiguating clause.

Second, we asked whether the difference in contextual discriminability varied across conditions. To test this, we asked whether an interaction between Order (First vs. Second) and Ambiguity Type significantly improved the fit of a model predicting Surprisal, above and beyond a model with only the main effects of Order and Ambiguity Type (and a random intercept for words). Again, the full model did not explain significantly more variance (*p* > 0.8).

These results are also depicted in Supplementary Figure 2. As illustrated, Surprisal was not systematically higher or lower in the First vs. Second context; similarly, this difference (and the direction of this difference) was not systematically different across conditions.


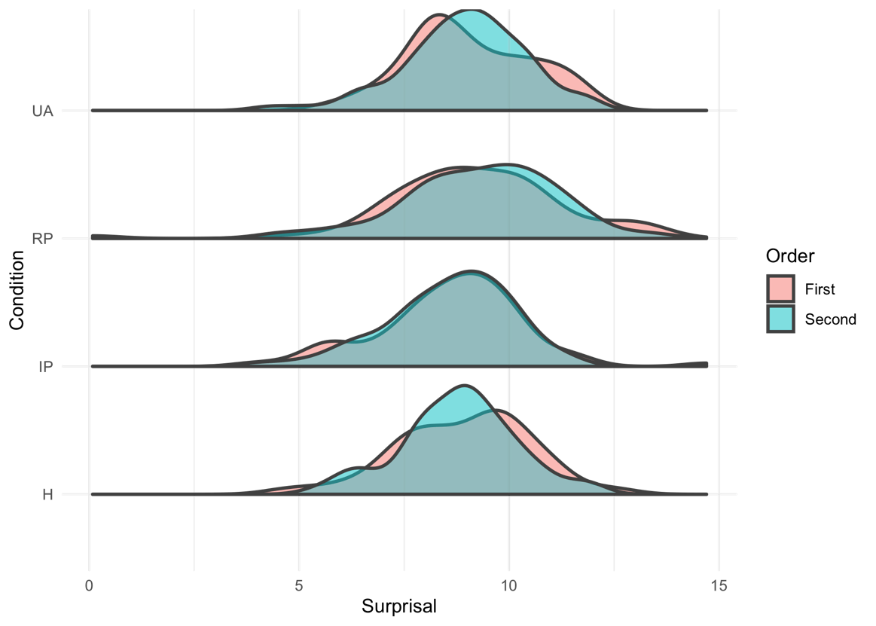


**Supplementary Figure 2.** Distribution of surprisal values for the target word appearing in either the First or Second disambiguating context, and divided by Condition (i.e., ambiguity type). There were no differences in Surprisal across contexts, nor did the effect of Order vary across Condition.
