# Supplementary material for: Offline dominance and zeugmatic similarity normings of variably ambiguous words assessed against a neural language model (BERT)
Source: Behav Res Methods. 2022 Jun 10;55(4):1537–57. doi: 10.3758/s13428-022-01869-6 (PMC10040203; doi:10.3758/s13428-022-01869-6)
Supplement: Supplementary file 2 — (DOCX 263 kb) [file 13428_2022_1869_MOESM2_ESM.docx]

|  | | | | | | | | | | |
| --- | --- | --- | --- | --- | --- | --- | --- | --- | --- | --- |
| **APPENDIX 1. STUDY 1 DOMINANCE NORMING SUMMARY DATA** | | | | | | | | | | |
|  |  |  |  |  |  |  |  |  |  |  |
| Column A: Dominance-normed ambiguous word. Items marked by an asterisk* have been rejected from data analysis for reasons described in the Notes Column K. Column B: Homonym (H) or Polyseme (P) categorization according to Wordsmyth Dictionary criterion, with H(+P) indicating a homonym with at least one meaning having greater than one polysemic sense. Column C: Total number of participant norming responses per item. Column D: Total number of ratings, including 1-2 raters per response for one of top 5 item definitions. Column E: Nth-most frequent definition based on Column C rated responses. Column F: Ambiguous word definition. Column G: Percentage of ratings aligning with Column F definition. Column H: Combined percentage of ratings accounted for by two most frequent definitions. Column I: Dominance score calculated as ([percentage of 1st most frequent minus percentage of 2nd most frequent definition]/percentage of 1st most frequent definition) with 0.00=perfectly balanced and 1.00=completely biased. Column J: Post-dominance norming re-categorization (if different than dictionary criteria) based on top 2 dominance norming definitions. Column K: Any notes or comments relating to item dominance norming. | | | | | | | | | | |
|  | | | | | | | | | | |
| Column A | B | C | D | E | F | G | H | I | J | K |
|  |  |  |  |  |  |  |  |  |  |  |
| Ambiguous  Word  (Asterisk  indicates  Not Rated) | (H)omonym, (P)olyseme,  or (NL)  Not Listed  in Dictionary | Number of Norming Responses | Total Number  of Definition 1 to 5 Response Ratings | Nth-Most Frequent Definition | Definition | Percentage  of Rated Responses Associated  with Definition | Percentage  Sum of 1st  and 2nd  Most  Frequent Definitions | Dominance Score | Post-  Dominance  Norming Ambiguous  Word  Category Based on Responses | Notes |
|  |  |  |  |  |  |  |  |  |  |  |
| **ACT** | P | 94 | 89 | 1st | perform in a dramatic work | 0.66 | 0.92 | 0.61 | H | Norming Def 2 is acronym and not listed in WS dictionary |
|  |  |  |  | 2nd | test | 0.26 |  |  |  |  |
|  |  |  |  | 3rd | performance of some physical or mental process; action | 0.08 |  |  |  |  |
| **ADDITION** | P | 189 | 359 | 1st | a mathematical operation in which the sum | 0.93 | 0.98 | 0.95 |  |  |
|  |  |  |  | 2nd | part added to a building or piece of land | 0.05 |  |  |  |  |
|  |  |  |  | 3rd | more | 0.02 |  |  |  |  |
| **ADMIT** | P | 95 | 187 | 1st | confess or acknowledge | 0.56 | 1.00 | 0.22 |  |  |
|  |  |  |  | 2nd | allow to enter; let in | 0.44 |  |  |  |  |
| **AFFAIR** | P | 96 | 182 | 1st | a sexual relationship between two people who are not married to each other | 0.85 | 1.00 | 0.83 |  |  |
|  |  |  |  | 2nd | an event or happening | 0.15 |  |  |  |  |
| **AIM** | P | 95 | 85 | 1st | to point (a weapon, missile, etc.) | 0.58 | 0.81 | 0.59 |  |  |
|  |  |  |  | 2nd | texting | 0.24 |  |  |  |  |
|  |  |  |  | 3rd | purpose or intention; a desired outcome | 0.19 |  |  |  |  |
| **ALLEY** | H(+P) | 93 | 168 | 1st | a narrow passageway between or behind buildings | 0.96 | 1.00 | 0.96 | P | 2nd WS Homonym def is obscure (marble) |
|  |  |  |  | 2nd | a long, narrow area in which games such as bowling are played | 0.04 |  |  |  |  |
| **ALLIGATOR** | P | 93 | 159 | 1st | a large semiaquatic reptile | 1.00 | 1.00 | 1.00 |  |  |
|  |  |  |  | 2nd | the skin of the alligator | 0.00 |  |  |  |  |
| **AMAZON** | P | 96 | 181 | 1st | online shopping | 0.70 | 1.00 | 0.57 | H | Company name is not listed in WS dictionary |
|  |  |  |  | 2nd | rainforest/jungle | 0.30 |  |  |  |  |
| **AMBER** | P | 96 | 86 | 1st | having the yellow color of amber | 0.36 | 0.69 | 0.10 |  |  |
|  |  |  |  | 2nd | hard translucent fossilized resin | 0.33 |  |  |  |  |
|  |  |  |  | 3rd | girl's name | 0.31 |  |  |  |  |
| **ANSWER** | P | 93 | 176 | 1st | a reply, either spoken or written, as to a question | 0.54 | 1.00 | 0.15 |  |  |
|  |  |  |  | 2nd | a solution, esp. of a mathematical problem | 0.46 |  |  |  |  |
| **APPEAL** | P | 94 | 179 | 1st | the power to attract, please, stimulate, or interest | 0.63 | 1.00 | 0.42 |  |  |
|  |  |  |  | 2nd | judicial review by a superior court of the decision of a lower tribunal | 0.37 |  |  |  |  |
| **APPLE** | P | 96 | 168 | 1st | fruit of the apple tree | 0.88 | 0.95 | 0.92 |  |  |
|  |  |  |  | 2nd | tree | 0.07 |  |  |  |  |
|  |  |  |  | 3rd | computer | 0.05 |  |  |  |  |
| **ARMS** | H(+P) | 96 | 182 | 1st | upper limbs of the human body | 0.80 | 0.99 | 0.75 |  |  |
|  |  |  |  | 2nd | weapons collectively | 0.20 |  |  |  |  |
|  |  |  |  | 3rd | branch | 0.01 |  |  |  |  |
| **ARTICLE** | P | 96 | 191 | 1st | a piece of writing included with others in a newspaper, magazine | 0.87 | 1.00 | 0.86 |  |  |
|  |  |  |  | 2nd | particular item or object, typically one of a specified type | 0.13 |  |  |  |  |
| **ATMOSPHERE** | P | 191 | 355 | 1st | envelope of gases surrounding the earth or another planet | 0.87 | 1.00 | 0.85 |  |  |
|  |  |  |  | 2nd | pervading tone or mood of a place, situation, or work of art | 0.13 |  |  |  |  |
| **BACK** | P | 96 | 73 | 1st | rear surface of the human body | 0.56 | 1.00 | 0.22 |  |  |
|  |  |  |  | 2nd | toward the rear; | 0.44 |  |  |  |  |
| **BAG** | P | 95 | 151 | 1st | a container made of flexible material | 0.81 | 1.00 | 0.76 |  |  |
|  |  |  |  | 2nd | contents of a bag | 0.19 |  |  |  |  |
| **BALANCE** | P | 93 | 162 | 1st | an even distribution of weight | 0.65 | 0.94 | 0.55 |  |  |
|  |  |  |  | 2nd | credit and debit totals of (an account) | 0.29 |  |  |  |  |
|  |  |  |  | 3rd | harmony | 0.06 |  |  |  |  |
| **BALL** | H(+P) | 96 | 186 | 1st | spherical or nearly spherical body or mass | 0.95 | 1.00 | 0.94 |  |  |
|  |  |  |  | 2nd | a social function for dancing, | 0.05 |  |  |  |  |
| **BAMBOO** | P | 190 | 292 | 1st | any tall treelike tropical or semitropical fast-growing grass | 0.88 | 1.00 | 0.86 |  |  |
|  |  |  |  | 2nd | stem of any of these plants, used for building, poles, and furniture | 0.12 |  |  |  |  |
| **BANANA** | P | 94 | 152 | 1st | crescent-shaped yellow fruit | 0.97 | 1.00 | 0.97 |  |  |
|  |  |  |  | 2nd | treelike plants | 0.03 |  |  |  |  |
| **BAND** | H(+P) | 192 | 356 | 1st | a group of musicians | 0.79 | 1.00 | 0.73 |  |  |
|  |  |  |  | 2nd | thin flat strip of some material | 0.21 |  |  |  |  |
| **BANK** | H(+P) | 96 | 185 | 1st | a financial establishment | 0.99 | 1.00 | 0.99 |  |  |
|  |  |  |  | 2nd | land alongside or sloping down to a river or lake | 0.01 |  |  |  |  |
| **BAR** | H(+P) | 96 | 169 | 1st | a counter across which alcoholic drinks or refreshments are served | 0.76 | 1.00 | 0.69 | P | 2nd WS Homonym def is obscure (unit of pressure) |
|  |  |  |  | 2nd | an amount of food or another substance formed into a regular narrow block | 0.24 |  |  |  |  |
| **BARK** | H(+P) | 93 | 179 | 1st | cry of certain animals, especially a dog | 0.58 | 1.00 | 0.26 |  |  |
|  |  |  |  | 2nd | protective outer sheath of the trunk, branches, and twigs of a tree | 0.42 |  |  |  |  |
| **BARREL** | P | 96 | 155 | 1st | cylindrical container bulging out in the middle, traditionally made of wooden staves | 0.72 | 0.94 | 0.69 |  |  |
|  |  |  |  | 2nd | contents | 0.22 |  |  |  |  |
|  |  |  |  | 3rd | a tube forming part of an object such as a gun or a pen | 0.06 |  |  |  |  |
| **BASE** | H(+P) | 95 | 162 | 1st | baseball: one of the four stations that must be reached in turn to score a run | 0.48 | 0.76 | 0.42 | P | 2nd WS Homonym def is obscure (inferior) |
|  |  |  |  | 2nd | the lowest part or edge of something, especially the part on which it rests or is supported | 0.28 |  |  |  |  |
|  |  |  |  | 3rd | main place where a person works or stays | 0.19 |  |  |  |  |
|  |  |  |  | 4th | chemistry | 0.03 |  |  |  |  |
|  |  |  |  | 5th | makeup | 0.02 |  |  |  |  |
| **BASEBALL** | P | 95 | 163 | 1st | a ball game played between two teams of nine | 0.77 | 1.00 | 0.71 |  |  |
|  |  |  |  | 2nd | the hard ball used in the game of baseball | 0.23 |  |  |  |  |
| **BASIL** | P | 92 | 127 | 1st | the leaves of the basil plant used as a culinary herb | 0.87 | 1.00 | 0.86 |  |  |
|  |  |  |  | 2nd | an aromatic annual plant | 0.13 |  |  |  |  |
| **BASKET** | P | 93 | 167 | 1st | a container used to hold or carry things | 0.59 | 0.81 | 0.63 |  |  |
|  |  |  |  | 2nd | a net fixed on a hoop used as the goal | 0.22 |  |  |  |  |
|  |  |  |  | 3rd | contents | 0.19 |  |  |  |  |
| **BASKETBALL** | P | 189 | 328 | 1st | a game played between two teams of five players | 0.75 | 1.00 | 0.67 |  |  |
|  |  |  |  | 2nd | the inflated ball used in the game of basketball | 0.25 |  |  |  |  |
| **BASS** | H(+P) | 93 | 171 | 1st | member of a family of instruments that is the lowest in pitch | 0.77 | 1.00 | 0.69 |  |  |
|  |  |  |  | 2nd | fish | 0.23 |  |  |  |  |
| **BAT** | H(+P) | 95 | 182 | 1st | nocturnal mammal | 0.67 | 1.00 | 0.51 |  |  |
|  |  |  |  | 2nd | used for hitting the ball in games such as baseball, | 0.33 |  |  |  |  |
| **BATTER** | H(+P) | 92 | 167 | 1st | a semiliquid mixture of flour, egg, and milk | 0.71 | 0.95 | 0.66 |  |  |
|  |  |  |  | 2nd | a player who is batting | 0.24 |  |  |  |  |
|  |  |  |  | 3rd | assault | 0.05 |  |  |  |  |
| **BEAM** | P | 95 | 182 | 1st | a ray or shaft of light | 0.53 | 0.92 | 0.28 |  |  |
|  |  |  |  | 2nd | long, sturdy piece of squared timber or metal | 0.38 |  |  |  |  |
|  |  |  |  | 3rd | smile radiantly | 0.08 |  |  |  |  |
| **BEANS** | P | 189 | 237 | 1st | an edible seed, typically kidney-shaped, growing in long pods on certain leguminous plants | 0.91 | 1.00 | 0.90 |  |  |
|  |  |  |  | 2nd | plant that bears beans in pods | 0.09 |  |  |  |  |
| **BEATLES** | P | 93 | 143 | 1st | rock 'n' roll singing group from Liverpool | 0.45 | 0.90 | 0.03 |  |  |
|  |  |  |  | 2nd | music of the Beatles | 0.44 |  |  |  |  |
|  |  |  |  | 3rd | animal | 0.10 |  |  |  |  |
| **BEETHOVEN** | P | 96 | 158 | 1st | music of Beethoven | 0.71 | 0.96 | 0.64 |  |  |
|  |  |  |  | 2nd | German composer | 0.25 |  |  |  |  |
|  |  |  |  | 3rd | dog | 0.04 |  |  |  |  |
| **BEETLE** | H(+P) | 190 | 256 | 1st | insect | 0.62 | 0.83 | 0.66 | ? | Other WS definitions obscure. Volkswagen definition is not listed; Beatles band is not spelled this way |
|  |  |  |  | 2nd | band | 0.21 |  |  |  |  |
|  |  |  |  | 3rd | Beetlejuice/movie | 0.13 |  |  |  |  |
|  |  |  |  | 4th | Volkswagen | 0.04 |  |  |  |  |
| **BEING** | P | 92 | 171 | 1st | a real or imaginary living creature | 0.54 | 1.00 | 0.14 |  |  |
|  |  |  |  | 2nd | existence | 0.46 |  |  |  |  |
| **BID** | P | 94 | 72 | 1st | an offer of a price, especially at an auction | 0.83 | 1.00 | 0.80 |  |  |
|  |  |  |  | 2nd | an attempt to obtain or do something | 0.17 |  |  |  |  |
| **BILL** | H(+P) | 188 | 354 | 1st | a banknote; a piece of paper money | 0.33 | 0.56 | 0.28 |  |  |
|  |  |  |  | 2nd | a boy's name | 0.24 |  |  |  |  |
|  |  |  |  | 3rd | an amount of money owed for goods supplied or services rendered | 0.21 |  |  |  |  |
|  |  |  |  | 4th | having to do with governmental law | 0.21 |  |  |  |  |
|  |  |  |  | 5th | beak | 0.02 |  |  |  |  |
| **BITTERNESS** | P | 93 | 179 | 1st | sharpness of taste; lack of sweetness | 0.63 | 1.00 | 0.40 |  |  |
|  |  |  |  | 2nd | anger and disappointment at being treated unfairly; resentment | 0.37 |  |  |  |  |
| **BLADE** | P | 189 | 317 | 1st | the flat cutting edge of a knife, saw, or other tool or weapon | 0.91 | 0.98 | 0.92 |  |  |
|  |  |  |  | 2nd | a long, narrow leaf of grass | 0.07 |  |  |  |  |
|  |  |  |  | 3rd | skate | 0.02 |  |  |  |  |
| **BLOCK** | P | 96 | 160 | 1st | any of a set of solid cubes used as a child's toy | 0.36 | 0.70 | 0.04 |  |  |
|  |  |  |  | 2nd | put an obstacle in the way of | 0.34 |  |  |  |  |
|  |  |  |  | 3rd | the area bounded by four streets in a town or suburb | 0.30 |  |  |  |  |
| **BLUFF** | H(+P) | 186 | 343 | 1st | an attempt to deceive someone | 0.76 | 1.00 | 0.68 |  |  |
|  |  |  |  | 2nd | a steep cliff, bank | 0.24 |  |  |  |  |
| **BOARD** | P | 96 | 128 | 1st | a surface on which to cut things, play games, or perform other activities | 0.59 | 0.84 | 0.56 |  |  |
|  |  |  |  | 2nd | the piece of equipment on which a person stands in surfing, skateboarding, snowboarding | 0.26 |  |  |  |  |
|  |  |  |  | 3rd | panel of people | 0.09 |  |  |  |  |
|  |  |  |  | 4th | get on something | 0.06 |  |  |  |  |
| **BOIL** | H(+P) | 93 | 177 | 1st | the temperature at which a liquid bubbles and turns to vapor | 0.96 | 1.00 | 0.96 |  |  |
|  |  |  |  | 2nd | an inflamed pus-filled swelling on the skin, typically caused by the infection of a hair follicle | 0.04 |  |  |  |  |
| **BOLT** | H(+P) | 93 | 90 | 1st | flash of lightning | 0.37 | 0.64 | 0.24 | P? | 2nd WS Homonym def is obscure (sift) and dog/Usain definitions are not in dictionary |
|  |  |  |  | 2nd | Disney dog | 0.28 |  |  |  |  |
|  |  |  |  | 3rd | run fast | 0.17 |  |  |  |  |
|  |  |  |  | 4th | Usain | 0.13 |  |  |  |  |
|  |  |  |  | 5th | screw/lock | 0.06 |  |  |  |  |
| **BOND** | P | 96 | 94 | 1st | joined | 0.59 | 0.93 | 0.42 | H | James Bond not in WS dictionary |
|  |  |  |  | 2nd | James | 0.34 |  |  |  |  |
|  |  |  |  | 3rd | monetary insurance | 0.07 |  |  |  |  |
| **BONE** | P | 93 | 36 | 1st | hard, whitish tissue making up the skeleton (in a living body) | 0.69 | 1.00 | 0.56 |  |  |
|  |  |  |  | 2nd | out of a living body | 0.31 |  |  |  |  |
| **BOOK** | P | 93 | 166 | 1st | the story the book conveys | 0.57 | 1.00 | 0.23 |  |  |
|  |  |  |  | 2nd | printed work consisting of pages glued or sewn together along one side and bound in covers | 0.43 |  |  |  |  |
| **BORDER** | P | 96 | 154 | 1st | a line separating two political or geographical areas | 0.78 | 1.00 | 0.72 |  |  |
|  |  |  |  | 2nd | the edge or boundary of something | 0.22 |  |  |  |  |
| **BORE** | H(+P) | 93 | 152 | 1st | make (someone) feel weary and uninterested by tedious talk or dullness | 0.89 | 1.00 | 0.88 |  |  |
|  |  |  |  | 2nd | make (a hole) | 0.11 |  |  |  |  |
| **BOTTLE** | P | 96 | 133 | 1st | a container, typically made of glass or plastic | 0.56 | 1.00 | 0.20 |  |  |
|  |  |  |  | 2nd | the contents of a bottle | 0.44 |  |  |  |  |
| **BOTTOM** | P | 95 | 145 | 1st | the lowest point or part | 0.86 | 1.00 | 0.84 |  |  |
|  |  |  |  | 2nd | the buttocks | 0.14 |  |  |  |  |
| **BOW** | H(+P) | 96 | 178 | 1st | a knot tied with two loops and two loose ends, used especially for tying shoelaces and decorative ribbons | 0.35 | 0.68 | 0.05 |  |  |
|  |  |  |  | 2nd | a weapon for shooting arrows | 0.33 |  |  |  |  |
|  |  |  |  | 3rd | bend the head or upper part of the body as a sign of respect, greeting, or shame | 0.32 |  |  |  |  |
| **BOWLER** | H | 89 | 167 | 1st | a player at tenpin bowling, lawn bowling | 0.82 | 1.00 | 0.78 |  |  |
|  |  |  |  | 2nd | hard felt hat | 0.18 |  |  |  |  |
| **BOXER** | H(+P) | 96 | 175 | 1st | a person who takes part in boxing, especially as a sport | 0.79 | 0.91 | 0.84 | P | 2nd WS Homonym def is obscure (Chinese secret society); dog def listed as polyseme |
|  |  |  |  | 2nd | medium-sized dog | 0.13 |  |  |  |  |
|  |  |  |  | 3rd | underwear | 0.09 |  |  |  |  |
| **BRASS** | P | 186 | 343 | 1st | yellow alloy of copper and zinc | 0.55 | 1.00 | 0.19 |  |  |
|  |  |  |  | 2nd | brass wind instruments (including trumpet, horn, trombone) | 0.45 |  |  |  |  |
| **BREAK** | P | 96 | 180 | 1st | a pause in work or during an activity or event | 0.51 | 1.00 | 0.04 |  |  |
|  |  |  |  | 2nd | an instance of breaking; the point where something is broken | 0.49 |  |  |  |  |
| **BRIDGE** | H(+P) | 92 | 80 | 1st | structure that extends | 0.99 | 1.00 | 0.99 |  |  |
|  |  |  |  | 2nd | card game | 0.01 |  |  |  |  |
| **BRIEFCASE** | P | 96 | 146 | 1st | flat, rectangular container, typically made of leather, for carrying books and papers | 0.73 | 1.00 | 0.64 |  |  |
|  |  |  |  | 2nd | contents of the briefcase | 0.27 |  |  |  |  |
| **BUCKET** | P | 93 | 114 | 1st | contents of the bucket | 0.56 | 1.00 | 0.22 |  |  |
|  |  |  |  | 2nd | cylindrical open container | 0.44 |  |  |  |  |
| **BUD** | H(+P) | 93 | 153 | 1st | growth on a plant that develops into a leaf, flower, or shoot | 0.59 | 0.86 | 0.56 |  |  |
|  |  |  |  | 2nd | beer | 0.26 |  |  |  |  |
|  |  |  |  | 3rd | friend | 0.14 |  |  |  |  |
| **BUG** | P | 93 | 73 | 1st | a small insect | 0.88 | 1.00 | 0.86 |  |  |
|  |  |  |  | 2nd | annoy or bother (someone) | 0.12 |  |  |  |  |
|  |  |  |  | 3rd | a miniature microphone | 0.00 |  |  |  |  |
| **BULB** | P | 94 | 168 | 1st | a light bulb | 0.82 | 1.00 | 0.78 |  |  |
|  |  |  |  | 2nd | of a plant/flower | 0.18 |  |  |  |  |
| **BUREAU** | P | 94 | 157 | 1st | office or department for transacting particular business | 0.98 | 1.00 | 0.98 |  |  |
|  |  |  |  | 2nd | a chest of drawers | 0.02 |  |  |  |  |
| **BUSINESS** | P | 96 | 90 | 1st | a person's regular occupation, profession, or trade | 0.90 | 1.00 | 0.89 |  |  |
|  |  |  |  | 2nd | the practice of making one's living by engaging in commerce | 0.10 |  |  |  |  |
| **BUTT** | H(+P) | 95 | 174 | 1st | rear end | 0.97 | 1.00 | 0.97 |  |  |
|  |  |  |  | 2nd | cigarette | 0.03 |  |  |  |  |
| **CABBAGE** | P | 96 | 76 | 1st | leaves of a head of cabbage, eaten as a vegetable | 0.66 | 1.00 | 0.48 |  |  |
|  |  |  |  | 2nd | cultivated plant | 0.34 |  |  |  |  |
| **CABINET** | P | 93 | 179 | 1st | a cupboard with drawers or shelves | 0.77 | 1.00 | 0.69 |  |  |
|  |  |  |  | 2nd | body of advisers to the president, | 0.23 |  |  |  |  |
| **CAGE** | P | 96 | 156 | 1st | space inside the cage | 0.76 | 0.91 | 0.80 |  |  |
|  |  |  |  | 2nd | a structure of bars or wires | 0.15 |  |  |  |  |
|  |  |  |  | 3rd | Nicholas Cage | 0.09 |  |  |  |  |
| **CALF** | H(+P) | 96 | 184 | 1st | a young cow | 0.60 | 1.00 | 0.33 |  |  |
|  |  |  |  | 2nd | back of a person's leg below the knee | 0.40 |  |  |  |  |
| **CALL** | P | 96 | 152 | 1st | speaking to someone on the phone | 0.87 | 1.00 | 0.85 |  |  |
|  |  |  |  | 2nd | a cry made as a summons or to attract someone's attention | 0.13 |  |  |  |  |
| **CANE** | P | 95 | 184 | 1st | walking stick, | 0.41 | 0.82 | 0.01 |  |  |
|  |  |  |  | 2nd | candy cane | 0.41 |  |  |  |  |
|  |  |  |  | 3rd | stalk, stick (not for walking) | 0.18 |  |  |  |  |
| **CANVAS** | P | 89 | 161 | 1st | cloth for an oil painting | 0.91 | 1.00 | 0.90 |  |  |
|  |  |  |  | 2nd | coarse unbleached cloth | 0.09 |  |  |  |  |
| **CAP** | H(+P) | 95 | 151 | 1st | soft, flat hat | 0.71 | 0.95 | 0.66 |  |  |
|  |  |  |  | 2nd | protective lid or cover for an object such as a bottle | 0.24 |  |  |  |  |
|  |  |  |  | 3rd | limit | 0.05 |  |  |  |  |
| **CAPE** | H | 92 | 156 | 1st | sleeveless cloak | 0.72 | 0.91 | 0.74 |  |  |
|  |  |  |  | 2nd | a headland or promontory | 0.19 |  |  |  |  |
|  |  |  |  | 3rd | evaluation | 0.09 |  |  |  |  |
| **CARD** | H(+P) | 96 | 145 | 1st | a playing card | 0.69 | 0.90 | 0.69 | P | 2nd WS Homonym def is obscure (wool) |
|  |  |  |  | 2nd | greeting card | 0.21 |  |  |  |  |
|  |  |  |  | 3rd | credit card/ID | 0.10 |  |  |  |  |
| **CARDINAL** | P | 96 | 136 | 1st | songbird | 0.70 | 0.91 | 0.69 |  |  |
|  |  |  |  | 2nd | dignitary of the Roman Catholic Church | 0.21 |  |  |  |  |
|  |  |  |  | 3rd | sports | 0.09 |  |  |  |  |
| **CARROTS** | P | 96 | 26 | 1st | eaten as a vegetable | 0.85 | 1.00 | 0.82 |  |  |
|  |  |  |  | 2nd | plant | 0.15 |  |  |  |  |
| **CARTON** | P | 93 | 119 | 1st | contents of a carton | 0.55 | 1.00 | 0.20 |  |  |
|  |  |  |  | 2nd | a light box or container | 0.45 |  |  |  |  |
| **CASE** | H(+P) | 188 | 312 | 1st | a container designed to hold or protect something | 0.47 | 0.78 | 0.35 |  |  |
|  |  |  |  | 2nd | a legal action, especially one to be decided in a court of law | 0.31 |  |  |  |  |
|  |  |  |  | 3rd | an instance of a particular situation | 0.22 |  |  |  |  |
| **CASSEROLE** | P | 93 | 160 | 1st | a kind of stew or side dish that is cooked slowly in an oven | 0.83 | 1.00 | 0.79 |  |  |
|  |  |  |  | 2nd | a large covered dish, typically of earthenware or glass, used for cooking casseroles | 0.18 |  |  |  |  |
| **CAST** | P | 95 | 149 | 1st | the actors taking part in a play, movie, or other production | 0.64 | 1.00 | 0.43 |  |  |
|  |  |  |  | 2nd | bandage stiffened with plaster of Paris, molded to the shape of a limb that is broken | 0.36 |  |  |  |  |
| **CAULDRON** | P | 94 | 120 | 1st | a large metal pot with a lid and handle | 0.74 | 1.00 | 0.65 |  |  |
|  |  |  |  | 2nd | contents of a cauldron | 0.26 |  |  |  |  |
| **CEDAR** | P | 91 | 108 | 1st | tree | 0.70 | 1.00 | 0.58 |  |  |
|  |  |  |  | 2nd | timber or wood from cedar tree | 0.30 |  |  |  |  |
| **CELL** | P | 93 | 177 | 1st | biology | 0.34 | 0.67 | 0.02 |  |  |
|  |  |  |  | 2nd | phone | 0.33 |  |  |  |  |
|  |  |  |  | 3rd | a small room in which a prisoner is locked up | 0.33 |  |  |  |  |
| **CHAIN** | P | 93 | 84 | 1st | connected flexible series of metal links | 0.76 | 1.00 | 0.69 |  |  |
|  |  |  |  | 2nd | successive reaction (e.g., chemistry, email) | 0.24 |  |  |  |  |
| **CHALLENGE** | p | 96 | 180 | 1st | a task or situation that tests someone's abilities | 0.71 | 1.00 | 0.58 |  |  |
|  |  |  |  | 2nd | a call to take part in a contest or competition, especially a duel | 0.29 |  |  |  |  |
| **CHANGE** | P | 93 | 181 | 1st | the act or instance of making or becoming different | 0.76 | 1.00 | 0.69 |  |  |
|  |  |  |  | 2nd | coins | 0.24 |  |  |  |  |
| **CHARACTER** | P | 94 | 78 | 1st | a person in a novel, play, or movie | 0.74 | 1.00 | 0.66 |  |  |
|  |  |  |  | 2nd | the mental and moral qualities distinctive to an individual | 0.26 |  |  |  |  |
| **CHARITY** | P | 189 | 317 | 1st | the voluntary giving of help, typically in the form of money, to those in need | 0.85 | 1.00 | 0.83 |  |  |
|  |  |  |  | 2nd | an organization set up to provide help and raise money for those in need | 0.15 |  |  |  |  |
| **CHARM** | P | 95 | 163 | 1st | appeal, charisma | 0.40 | 0.71 | 0.23 |  |  |
|  |  |  |  | 2nd | a small ornament worn on a necklace or bracelet | 0.31 |  |  |  |  |
|  |  |  |  | 3rd | an object, act, or saying believed to have magic power | 0.29 |  |  |  |  |
| **CHECK** | P | 96 | 78 | 1st | a written order to a bank to pay a stated sum from the drawer's account | 0.73 | 1.00 | 0.63 |  |  |
|  |  |  |  | 2nd | examine (something) in order to determine its accuracy, quality, or condition | 0.27 |  |  |  |  |
| **CHERRY** | P | 93 | 153 | 1st | a small, round stone fruit that is typically bright or dark red | 0.78 | 1.00 | 0.71 |  |  |
|  |  |  |  | 2nd | the tree that bears the cherry | 0.22 |  |  |  |  |
| **CHEST** | P | 189 | 346 | 1st | the front surface of a person's or animal's body | 0.75 | 1.00 | 0.67 |  |  |
|  |  |  |  | 2nd | a large strong box, | 0.25 |  |  |  |  |
| **CHESTNUT** | P | 94 | 173 | 1st | a glossy brown nut that may be roasted and eaten | 0.94 | 1.00 | 0.93 |  |  |
|  |  |  |  | 2nd | deep reddish-brown color | 0.06 |  |  |  |  |
| **CHEVY** | NL | 92 | 154 | 1st | car | 0.90 | 1.00 | 0.88 | P | Car brand name is not listed in WS dictionary |
|  |  |  |  | 2nd | car manufacturer/company | 0.10 |  |  |  |  |
| **CHICKEN** | P | 94 | 150 | 1st | meat from a chicken | 0.78 | 1.00 | 0.72 |  |  |
|  |  |  |  | 2nd | a domestic fowl | 0.22 |  |  |  |  |
| **CHIMNEY** | P | 96 | 168 | 1st | the space inside a chimney | 0.81 | 1.00 | 0.76 |  |  |
|  |  |  |  | 2nd | a vertical channel or pipe that conducts smoke | 0.19 |  |  |  |  |
| **CHINA** | H(+P) | 92 | 171 | 1st | the country | 0.91 | 1.00 | 0.90 |  |  |
|  |  |  |  | 2nd | household tableware | 0.09 |  |  |  |  |
| **CHINESE** | P | 189 | 253 | 1st | language | 0.68 | 1.00 | 0.52 |  |  |
|  |  |  |  | 2nd | food | 0.32 |  |  |  |  |
| **CHIP** | H(+P) | 93 | 152 | 1st | a thin slice of food made crisp by being fried, baked, or dried and typically eaten as a snack | 0.78 | 1.00 | 0.71 | P | 2nd WS Homonym def is obscure (chirp) |
|  |  |  |  | 2nd | short for microchip | 0.22 |  |  |  |  |
| **CHOCOLATE** | P | 95 | 76 | 1st | a food preparation in the form of a paste or solid block made from roasted and ground cacao seeds, typically sweetened | 0.92 | 1.00 | 0.91 |  |  |
|  |  |  |  | 2nd | a deep brown color | 0.08 |  |  |  |  |
| **CHOP** | H(+P) | 96 | 84 | 1st | cut (something) into small pieces | 0.76 | 0.90 | 0.81 |  |  |
|  |  |  |  | 2nd | a downward cutting blow or movement, typically with the hand | 0.14 |  |  |  |  |
|  |  |  |  | 3rd | a thick slice of meat | 0.10 |  |  |  |  |
| **CHURCH** | P | 95 | 147 | 1st | a particular Christian organization, | 0.71 | 1.00 | 0.59 |  |  |
|  |  |  |  | 2nd | a building used for public Christian worship | 0.29 |  |  |  |  |
| **CINEMA** | P | 96 | 79 | 1st | a movie theater | 0.81 | 1.00 | 0.77 |  |  |
|  |  |  |  | 2nd | the production of movies | 0.19 |  |  |  |  |
| **CIRCLE** | P | 96 | 176 | 1st | a round plane figure | 0.88 | 1.00 | 0.86 |  |  |
|  |  |  |  | 2nd | a group of people with shared professions, interests, or acquaintances | 0.13 |  |  |  |  |
| **CIRCUIT** | P | 95 | 171 | 1st | path around which a circulating electric current can flow | 0.86 | 1.00 | 0.84 |  |  |
|  |  |  |  | 2nd | a series of athletic exercises | 0.14 |  |  |  |  |
| **CLASH** | P | 96 | 171 | 1st | a violent confrontation | 0.73 | 1.00 | 0.63 |  |  |
|  |  |  |  | 2nd | a loud jarring sound | 0.27 |  |  |  |  |
| **CLASP** | P | 91 | 82 | 1st | grasp (something) tightly with one's hand | 0.55 | 1.00 | 0.18 |  |  |
|  |  |  |  | 2nd | a device with interlocking parts used for fastening things together | 0.45 |  |  |  |  |
| **CLINK** | P | 93 | 80 | 1st | a sharp ringing sound, | 0.98 | 1.00 | 0.97 |  |  |
|  |  |  |  | 2nd | slang jail | 0.03 |  |  |  |  |
| **CLOG** | P | 95 | 93 | 1st | block or become blocked | 0.62 | 1.00 | 0.40 |  |  |
|  |  |  |  | 2nd | a shoe with a thick wooden sole | 0.38 |  |  |  |  |
| **CLOSE** | P | 96 | 91 | 1st | move or cause to move so as to cover an opening | 0.57 | 1.00 | 0.25 |  |  |
|  |  |  |  | 2nd | nearby | 0.43 |  |  |  |  |
| **CLUB** | P | 96 | 165 | 1st | an association or organization | 0.55 | 0.92 | 0.31 |  |  |
|  |  |  |  | 2nd | nightclub | 0.38 |  |  |  |  |
|  |  |  |  | 3rd | stick | 0.08 |  |  |  |  |
| **COACH** | P | 191 | 329 | 1st | an athletic instructor or trainer | 0.76 | 1.00 | 0.68 |  |  |
|  |  |  |  | 2nd | purse | 0.24 |  |  |  |  |
| **COAT** | P | 96 | 180 | 1st | an outer garment worn outdoors | 0.91 | 1.00 | 0.90 |  |  |
|  |  |  |  | 2nd | a covering of paint | 0.09 |  |  |  |  |
| **COFFEE** | P | 96 | 165 | 1st | a drink | 0.96 | 1.00 | 0.96 |  |  |
|  |  |  |  | 2nd | pale brown color | 0.04 |  |  |  |  |
| **COLD** | P | 96 | 170 | 1st | low or relatively low temperature | 0.80 | 1.00 | 0.75 |  |  |
|  |  |  |  | 2nd | a common viral infection | 0.20 |  |  |  |  |
| **COLON** | H(+P) | 189 | 351 | 1st | part of the large intestine | 0.74 | 1.00 | 0.64 |  |  |
|  |  |  |  | 2nd | punctuation mark | 0.26 |  |  |  |  |
| **COLUMN** | P | 96 | 166 | 1st | vertical division of a page or text | 0.54 | 1.00 | 0.16 |  |  |
|  |  |  |  | 2nd | upright pillar, | 0.46 |  |  |  |  |
| **COMMERCIAL** | P | 93 | 83 | 1st | a television or radio advertisement | 0.83 | 1.00 | 0.80 |  |  |
|  |  |  |  | 2nd | making or intended to make a profit | 0.17 |  |  |  |  |
| **COMPANY** | P | 95 | 180 | 1st | a commercial business | 0.83 | 1.00 | 0.79 |  |  |
|  |  |  |  | 2nd | companionship | 0.17 |  |  |  |  |
| **CONDITION** | P | 95 | 174 | 1st | the state of something, | 0.78 | 1.00 | 0.72 |  |  |
|  |  |  |  | 2nd | apply something to (the skin or hair) to give it a healthy or attractive look or feel | 0.22 |  |  |  |  |
| **COPPER** | P | 96 | 153 | 1st | reddish metallic element | 0.82 | 1.00 | 0.79 |  |  |
|  |  |  |  | 2nd | reddish-brown color | 0.18 |  |  |  |  |
| **CORAL** | P | 186 | 356 | 1st | skeleton of certain of sea animals | 0.74 | 1.00 | 0.64 |  |  |
|  |  |  |  | 2nd | deep-pink to yellowish-pink color | 0.26 |  |  |  |  |
| **CORE** | P | 96 | 118 | 1st | series of muscles, extending far beyond your abs | 0.39 | 0.75 | 0.09 |  |  |
|  |  |  |  | 2nd | central part of certain fleshy fruits, such as the apple or pear | 0.36 |  |  |  |  |
|  |  |  |  | 3rd | central part of the earth | 0.25 |  |  |  |  |
| **CORN** | H(+P) | 95 | 130 | 1st | edible vegetable | 0.58 | 1.00 | 0.29 | P | Homonym (foot) meaning not supplied in norming--only polyseme |
|  |  |  |  | 2nd | plant that yields large grains, or kernels, set in rows on a cob | 0.42 |  |  |  |  |
| **CORRESPONDENT** | P | 92 | 154 | 1st | person who communicates by letter or by letters | 0.63 | 1.00 | 0.41 |  |  |
|  |  |  |  | 2nd | person employed by a newspaper, etc., to report | 0.37 |  |  |  |  |
| **COTTON** | P | 96 | 139 | 1st | soft white downy fiber of cotton plant | 0.66 | 1.00 | 0.49 |  |  |
|  |  |  |  | 2nd | fabric | 0.34 |  |  |  |  |
| **COUNT** | H(+P) | 96 | 86 | 1st | to add up or check | 0.70 | 1.00 | 0.57 |  |  |
|  |  |  |  | 2nd | a nobleman | 0.30 |  |  |  |  |
| **COUNTER** | H(+P) | 93 | 89 | 1st | horizontal surface | 0.70 | 1.00 | 0.56 |  |  |
|  |  |  |  | 2nd | to say or do (something) in retaliation or response | 0.30 |  |  |  |  |
| **COUNTRY** | P | 96 | 172 | 1st | nation, territory distinguished by its people, culture, language, geography, etc. | 0.77 | 0.98 | 0.74 |  |  |
|  |  |  |  | 2nd | short for country music | 0.20 |  |  |  |  |
|  |  |  |  | 3rd | rural | 0.02 |  |  |  |  |
| **COURT** | P | 96 | 176 | 1st | tribunal presided over by a judge, judges, | 0.73 | 1.00 | 0.63 |  |  |
|  |  |  |  | 2nd | a quadrangular area, either open or covered, marked out for ball games such as tennis or basketball | 0.27 |  |  |  |  |
| **COVER** | P | 94 | 156 | 1st | anything that covers, spreads over, protects, or conceals | 0.80 | 1.00 | 0.75 |  |  |
|  |  |  |  | 2nd | blanket/bedspread | 0.20 |  |  |  |  |
| **CRAFT** | P | 96 | 168 | 1st | an activity involving skill in making things by hand | 0.99 | 1.00 | 0.99 |  |  |
|  |  |  |  | 2nd | a boat or ship | 0.01 |  |  |  |  |
| **CRANE** | P | 92 | 162 | 1st | tall, long-legged, long-necked bird, | 0.59 | 1.00 | 0.31 |  |  |
|  |  |  |  | 2nd | a large, tall machine used for moving heavy objects | 0.41 |  |  |  |  |
| **CRICKET** | H(+P) | 96 | 158 | 1st | an insect related to the grasshoppers | 0.73 | 1.00 | 0.64 |  |  |
|  |  |  |  | 2nd | game played on a large grass field with ball, bats, | 0.27 |  |  |  |  |
| **CROCODILE** | P | 190 | 221 | 1st | predatory semiaquatic reptile | 0.90 | 1.00 | 0.89 |  |  |
|  |  |  |  | 2nd | leather made from the skin of crocodile | 0.10 |  |  |  |  |
| **CRUST** | P | 95 | 163 | 1st | baked shell of a pie, tart, etc. | 0.88 | 1.00 | 0.87 |  |  |
|  |  |  |  | 2nd | outer shell of the earth, | 0.12 |  |  |  |  |
| **CUE** | H(+P) | 95 | 174 | 1st | a signal or reminder to do something | 0.77 | 1.00 | 0.70 |  |  |
|  |  |  |  | 2nd | billiards, snooker a long tapered shaft with a leather tip, used to drive the balls | 0.23 |  |  |  |  |
| **CULT** | P | 184 | 309 | 1st | specific system of religious worship | 0.91 | 1.00 | 0.90 |  |  |
|  |  |  |  | 2nd | something regarded as fashionable | 0.09 |  |  |  |  |
| **CUP** | P | 95 | 132 | 1st | contents of a cup container | 0.64 | 1.00 | 0.45 |  |  |
|  |  |  |  | 2nd | small open container | 0.36 |  |  |  |  |
| **CURSE** | P | 95 | 167 | 1st | an appeal to a supernatural power for harm to come to a specific person, group, etc. | 0.62 | 1.00 | 0.38 |  |  |
|  |  |  |  | 2nd | swear, obscene expression | 0.38 |  |  |  |  |
| **CYCLE** | P | 96 | 184 | 1st | short for bicycle, tricycle, motorcycle | 0.55 | 1.00 | 0.18 |  |  |
|  |  |  |  | 2nd | events repeating themselves in a regular sequence | 0.45 |  |  |  |  |
| **DANISH** | P | 93 | 87 | 1st | pastry | 0.63 | 1.00 | 0.42 |  |  |
|  |  |  |  | 2nd | characteristic of Denmark, its people, or their language | 0.37 |  |  |  |  |
| **DATE** | H(+P) | 96 | 169 | 1st | an appointment for a particular time, esp. with a person to whom one is sexually or romantically attached | 0.51 | 0.87 | 0.31 |  |  |
|  |  |  |  | 2nd | specified day of the month | 0.36 |  |  |  |  |
|  |  |  |  | 3rd | fruit of the date palm | 0.13 |  |  |  |  |
| **DEAL** | H(+P) | 94 | 80 | 1st | bargain, transaction, or agreement | 0.74 | 1.00 | 0.64 | P | 2nd WS Homonym def is obscure (planks) |
|  |  |  |  | 2nd | process of distributing the cards | 0.26 |  |  |  |  |
| **DECK** | P | 96 | 157 | 1st | nautical any of various platforms built into a vessel | 0.68 | 0.90 | 0.67 |  |  |
|  |  |  |  | 2nd | pack of playing cards | 0.22 |  |  |  |  |
|  |  |  |  | 3rd | wooden backyard | 0.10 |  |  |  |  |
| **DEED** | P | 93 | 170 | 1st | something that is done or performed; act | 0.56 | 1.00 | 0.21 |  |  |
|  |  |  |  | 2nd | legal document | 0.44 |  |  |  |  |
| **DEGREE** | P | 96 | 180 | 1st | academic award conferred by a university or college | 0.63 | 1.00 | 0.42 |  |  |
|  |  |  |  | 2nd | stage in a scale of relative amount or intensity | 0.37 |  |  |  |  |
| **DESIGN** | P | 96 | 90 | 1st | art of planning and making detailed drawings of something | 0.67 | 1.00 | 0.50 |  |  |
|  |  |  |  | 2nd | finished artistic or decorative creation | 0.33 |  |  |  |  |
| **DEVELOPMENT** | P | 95 | 179 | 1st | act or process of growing | 0.80 | 0.97 | 0.79 |  |  |
|  |  |  |  | 2nd | a fact, event, or happening, esp. one that changes a situation | 0.17 |  |  |  |  |
|  |  |  |  | 3rd | improving by expanding or enlarging or refining | 0.03 |  |  |  |  |
| **DIAMOND** | P | 93 | 177 | 1st | gemstone | 0.92 | 1.00 | 0.91 |  |  |
|  |  |  |  | 2nd | geometry: figure having four sides of equal length forming two acute angles and two obtuse angles; rhombus | 0.08 |  |  |  |  |
| **DIET** | H(+P) | 92 | 160 | 1st | a specific allowance or selection of food, esp. prescribed to control weight | 0.63 | 1.00 | 0.40 | P | 2nd WS Homonym def is obscure (legislative body) |
|  |  |  |  | 2nd | the food and drink that a person or animal regularly consumes | 0.38 |  |  |  |  |
| **DIFFERENCE** | P | 187 | 315 | 1st | a significant change in a situation | 0.69 | 1.00 | 0.54 |  |  |
|  |  |  |  | 2nd | result of the subtraction | 0.31 |  |  |  |  |
| **DIGEST** | P | 93 | 90 | 1st | to subject (food) to a process of digestion | 0.84 | 1.00 | 0.82 |  |  |
|  |  |  |  | 2nd | a magazine, periodical, etc., | 0.16 |  |  |  |  |
| **DIP** | P | 96 | 153 | 1st | thick creamy sauce | 0.69 | 1.00 | 0.54 |  |  |
|  |  |  |  | 2nd | a brief swim in water | 0.31 |  |  |  |  |
| **DIRT** | P | 93 | 178 | 1st | any unclean substance, such as mud, dust, | 0.96 | 1.00 | 0.96 |  |  |
|  |  |  |  | 2nd | gossip; scandalous information | 0.04 |  |  |  |  |
| **DISCHARGE** | P | 93 | 153 | 1st | dismissal or release | 0.42 | 0.75 | 0.25 |  |  |
|  |  |  |  | 2nd | pouring forth of a fluid; emission | 0.32 |  |  |  |  |
|  |  |  |  | 3rd | lose or remove electric charge | 0.25 |  |  |  |  |
| **DISH** | P | 96 | 163 | 1st | container used for holding or serving | 0.83 | 1.00 | 0.80 |  |  |
|  |  |  |  | 2nd | the food that is served or contained in a dish | 0.17 |  |  |  |  |
| **DIVISION** | P | 96 | 163 | 1st | a mathematical operation, | 0.42 | 0.72 | 0.30 |  |  |
|  |  |  |  | 2nd | the act of dividing or state of being divided | 0.29 |  |  |  |  |
|  |  |  |  | 3rd | (in sports) a section, category, or class organized | 0.28 |  |  |  |  |
| **DOLL** | P | 189 | 334 | 1st | model or dummy of a human being, used as a toy | 0.81 | 1.00 | 0.77 |  |  |
|  |  |  |  | 2nd | a pretty girl or woman | 0.19 |  |  |  |  |
| **DOOR** | P | 96 | 176 | 1st | a hinged or sliding panel for closing the entrance | 0.57 | 1.00 | 0.26 |  |  |
|  |  |  |  | 2nd | a doorway or entrance to a room or building | 0.43 |  |  |  |  |
| **DOUGH** | P | 93 | 184 | 1st | thick mixture of flour or meal and water or milk | 0.88 | 1.00 | 0.86 |  |  |
|  |  |  |  | 2nd | a slang word for money | 0.12 |  |  |  |  |
| **DRAFT** | P | 93 | 173 | 1st | a preliminary version of a piece of writing | 0.51 | 0.75 | 0.55 |  |  |
|  |  |  |  | 2nd | selection for compulsory military service (or team) | 0.23 |  |  |  |  |
|  |  |  |  | 3rd | air/breeze | 0.14 |  |  |  |  |
|  |  |  |  | 4th | beer or other drink that is kept in and served from a barrel or tank | 0.12 |  |  |  |  |
| **DRAG** | P | 94 | 151 | 1st | pull (someone or something) along forcefully, roughly, or with difficulty | 0.50 | 0.82 | 0.35 |  |  |
|  |  |  |  | 2nd | women's clothes worn by a man | 0.32 |  |  |  |  |
|  |  |  |  | 3rd | engage in a drag race | 0.12 |  |  |  |  |
|  |  |  |  | 4th | boring/bummer | 0.06 |  |  |  |  |
| **DRAPE** | P | 93 | 71 | 1st | long curtains | 0.65 | 1.00 | 0.46 |  |  |
|  |  |  |  | 2nd | arrange (cloth or clothing) | 0.35 |  |  |  |  |
| **DRILL** | H(+P) | 189 | 355 | 1st | a hand tool, | 0.60 | 1.00 | 0.33 | P | Other WS Homonym defs obscure (furrow, fabric) |
|  |  |  |  | 2nd | routine exercise or activity, in which people practice what they should do in dangerous situation | 0.40 |  |  |  |  |
| **DRINK** | P | 94 | 171 | 1st | liquid suitable for drinking; any beverage | 0.71 | 1.00 | 0.59 |  |  |
|  |  |  |  | 2nd | alcoholic drink | 0.29 |  |  |  |  |
| **DROP** | P | 95 | 172 | 1st | fall vertically | 0.58 | 0.86 | 0.52 |  |  |
|  |  |  |  | 2nd | small quantity of liquid that forms or falls | 0.28 |  |  |  |  |
|  |  |  |  | 3rd | switch of rhythm or bass line | 0.14 |  |  |  |  |
| **DRUMSTICK** | P | 96 | 187 | 1st | stick used for playing a drum | 0.44 | 0.78 | 0.23 |  |  |
|  |  |  |  | 2nd | lower joint of the leg of a cooked fowl | 0.34 |  |  |  |  |
|  |  |  |  | 3rd | ice cream | 0.22 |  |  |  |  |
| **EARTH** | P | 93 | 165 | 1st | third planet from the sun | 0.79 | 1.00 | 0.73 |  |  |
|  |  |  |  | 2nd | land; ground | 0.21 |  |  |  |  |
| **EGGS** | H(+P) | 96 | 173 | 1st | egg of the domestic hen used as food | 0.79 | 1.00 | 0.73 | P | Homonym meaning (encourage) not supplied in norming--only polyseme |
|  |  |  |  | 2nd | the oval reproductive body laid by the females of birds and some other animals, with outer shell or membrane | 0.21 |  |  |  |  |
| **ELBOW** | P | 95 | 179 | 1st | joint between the upper arm and the forearm | 1.00 | 1.00 | 1.00 |  |  |
|  |  |  |  | 2nd | part of a garment that covers the elbow | 0.00 |  |  |  |  |
| **ELVIS** | NL | 93 | 88 | 1st | singer | 0.72 | 1.00 | 0.60 | P | Not listed in WS dictionary |
|  |  |  |  | 2nd | rock n roll music | 0.28 |  |  |  |  |
| **EMERALD** | P | 96 | 110 | 1st | a gem | 0.70 | 1.00 | 0.57 |  |  |
|  |  |  |  | 2nd | green color | 0.30 |  |  |  |  |
| **ENGINEER** | P | 96 | 141 | 1st | person trained in any branch of the profession of engineering | 1.00 | 1.00 | 1.00 |  |  |
|  |  |  |  | 2nd | driver of a railway locomotive | 0.00 |  |  |  |  |
| **EXHAUST** | P | 95 | 91 | 1st | gases ejected from an engine | 0.59 | 1.00 | 0.31 |  |  |
|  |  |  |  | 2nd | drain the energy of; tire out | 0.41 |  |  |  |  |
| **FAIR** | H(+P) | 93 | 69 | 1st | travelling entertainment with sideshows, rides, etc. | 0.55 | 1.00 | 0.18 |  |  |
|  |  |  |  | 2nd | just; impartial | 0.45 |  |  |  |  |
| **FALL** | P | 92 | 88 | 1st | autumn | 0.84 | 1.00 | 0.81 |  |  |
|  |  |  |  | 2nd | descend by the force of gravity from a higher to a lower place | 0.16 |  |  |  |  |
| **FAN** | H(+P) | 93 | 177 | 1st | device for creating a current of air | 0.75 | 1.00 | 0.67 |  |  |
|  |  |  |  | 2nd | an ardent admirer | 0.25 |  |  |  |  |
| **FAT** | P | 92 | 167 | 1st | corpulence, obesity, or plumpness | 0.74 | 1.00 | 0.64 |  |  |
|  |  |  |  | 2nd | vegetable or animal tissue containing fat | 0.26 |  |  |  |  |
| **FAULT** | P | 93 | 147 | 1st | responsibility for a mistake or misdeed | 0.55 | 1.00 | 0.19 |  |  |
|  |  |  |  | 2nd | fracture in the earth's crust | 0.45 |  |  |  |  |
| **FEELING** | P | 95 | 171 | 1st | emotional or moral sensitivity | 0.88 | 1.00 | 0.86 |  |  |
|  |  |  |  | 2nd | the sense of touch | 0.12 |  |  |  |  |
| **FELLOW** | P | 95 | 168 | 1st | associate, friend | 0.78 | 1.00 | 0.72 |  |  |
|  |  |  |  | 2nd | a man or boy | 0.22 |  |  |  |  |
| **FENCING** | P | 95 | 176 | 1st | sport of fighting with swords | 0.86 | 1.00 | 0.84 |  | fence was search term |
|  |  |  |  | 2nd | wire, stakes, etc., used as fences | 0.14 |  |  |  |  |
| **FIELD** | P | 96 | 158 | 1st | a limited or marked off area, usually of mown grass, on which any of various sports are played | 0.58 | 0.88 | 0.47 |  |  |
|  |  |  |  | 2nd | open tract of uncultivated grassland | 0.30 |  |  |  |  |
|  |  |  |  | 3rd | a subject of which one studies or works in | 0.12 |  |  |  |  |
| **FILE** | H(+P) | 190 | 308 | 1st | a folder, box, etc., used to keep documents | 0.72 | 0.92 | 0.71 |  |  |
|  |  |  |  | 2nd | computerized collection of information | 0.20 |  |  |  |  |
|  |  |  |  | 3rd | tool used for shaping | 0.08 |  |  |  |  |
| **FILLING** | P | 95 | 162 | 1st | substance or thing used to fill a space or container, e.g., pastry | 0.67 | 1.00 | 0.50 |  |  |
|  |  |  |  | 2nd | substance filling a cavity of a tooth | 0.33 |  |  |  |  |
| **FILM** | P | 192 | 375 | 1st | movie | 0.98 | 1.00 | 0.98 |  |  |
|  |  |  |  | 2nd | thin flexible strip of cellulose coated with a photographic emulsion, used to make negatives and transparencies | 0.02 |  |  |  |  |
| **FINE** | P | 95 | 84 | 1st | okay | 0.42 | 0.62 | 0.51 |  |  |
|  |  |  |  | 2nd | money exacted as a penalty | 0.20 |  |  |  |  |
|  |  |  |  | 3rd | very good of its kind | 0.19 |  |  |  |  |
|  |  |  |  | 4th | thin/small | 0.19 |  |  |  |  |
| **FINGERS** | P | 192 | 350 | 1st | any of the digits of the hand | 0.94 | 1.00 | 0.94 |  |  |
|  |  |  |  | 2nd | something that resembles a finger in shape | 0.06 |  |  |  |  |
| **FISH** | P | 96 | 131 | 1st | cold-blooded aquatic vertebrates | 0.79 | 1.00 | 0.74 |  |  |
|  |  |  |  | 2nd | flesh of fish used as food | 0.21 |  |  |  |  |
| **FIT** | H(+P) | 93 | 81 | 1st | in good health/shape | 0.96 | 1.00 | 0.96 |  |  |
|  |  |  |  | 2nd | sudden spell of emotion | 0.04 |  |  |  |  |
| **FLING** | P | 92 | 81 | 1st | period or occasion of unrestrained, impulsive, or extravagant behavior | 0.56 | 1.00 | 0.20 |  |  |
|  |  |  |  | 2nd | to throw, esp. with force or abandon | 0.44 |  |  |  |  |
| **FLOOR** | P | 96 | 87 | 1st | lower surface of a room | 0.80 | 1.00 | 0.76 |  |  |
|  |  |  |  | 2nd | a story of a building | 0.20 |  |  |  |  |
| **FLY** | H(+P) | 96 | 80 | 1st | to move through the air | 0.56 | 1.00 | 0.22 |  |  |
|  |  |  |  | 2nd | insect, esp. the housefly | 0.44 |  |  |  |  |
| **FOOT** | P | 96 | 175 | 1st | part of the leg below the ankle joint | 0.88 | 1.00 | 0.86 |  |  |
|  |  |  |  | 2nd | unit of length | 0.12 |  |  |  |  |
| **FOOTBALL** | P | 96 | 183 | 1st | game played with a round or oval ball | 0.94 | 1.00 | 0.94 |  |  |
|  |  |  |  | 2nd | the ball used in any of these games | 0.06 |  |  |  |  |
| **FORD** | NL | 95 | 151 | 1st | car | 0.82 | 0.99 | 0.80 | P | Company name is not listed in WS dictionary |
|  |  |  |  | 2nd | automobile brand/manufacturer/corporation | 0.17 |  |  |  |  |
|  |  |  |  | 3rd | river | 0.01 |  |  |  |  |
| **FORK** | P | 93 | 179 | 1st | metal implement used for lifting food to the mouth | 0.94 | 1.00 | 0.94 |  |  |
|  |  |  |  | 2nd | division of a road/river into two or more branches | 0.06 |  |  |  |  |
| **FORTUNE** | P | 95 | 179 | 1st | a person's destiny | 0.66 | 1.00 | 0.48 |  |  |
|  |  |  |  | 2nd | great amount of wealth | 0.34 |  |  |  |  |
| **FORWARD** | P | 92 | 84 | 1st | directed or moving ahead | 0.96 | 1.00 | 0.96 |  |  |
|  |  |  |  | 2nd | send forward or pass on to an ultimate destination | 0.04 |  |  |  |  |
| **FRENCH** | P | 96 | 86 | 1st | relating to, denoting, or characteristic of France, the French | 0.90 | 1.00 | 0.88 |  |  |
|  |  |  |  | 2nd | language | 0.10 |  |  |  |  |
| **FUNCTION** | P | 92 | 83 | 1st | math relation between two sets | 0.54 | 0.95 | 0.24 |  |  |
|  |  |  |  | 2nd | work properly | 0.41 |  |  |  |  |
|  |  |  |  | 3rd | official or formal social gathering or ceremony | 0.05 |  |  |  |  |
| **FUR** | P | 94 | 163 | 1st | dense coat of fine silky hairs on such mammals | 0.71 | 1.00 | 0.59 |  |  |
|  |  |  |  | 2nd | garment made of fur, such as a coat or stole | 0.29 |  |  |  |  |
| **GAG** | H(+P) | 95 | 77 | 1st | retch or cause to retch | 0.77 | 1.00 | 0.69 |  |  |
|  |  |  |  | 2nd | a joke or humorous story | 0.23 |  |  |  |  |
| **GAME** | P | 93 | 180 | 1st | contest with rules, the result being determined by skill, strength, or chance | 0.56 | 1.00 | 0.22 |  |  |
|  |  |  |  | 2nd | equipment needed for playing certain games | 0.44 |  |  |  |  |
| **GAS** | P | 95 | 172 | 1st | gasoline | 0.87 | 0.95 | 0.91 |  |  |
|  |  |  |  | 2nd | flatulence | 0.08 |  |  |  |  |
|  |  |  |  | 3rd | state of matter | 0.05 |  |  |  |  |
| **GATE** | P | 96 | 134 | 1st | a movable barrier in a fence or wall | 0.64 | 1.00 | 0.44 |  |  |
|  |  |  |  | 2nd | an opening to allow passage into or out of an enclosed place | 0.36 |  |  |  |  |
| **GEAR** | P | 92 | 168 | 1st | a toothed wheel that engages with another toothed wheel or with a rack in order to change the speed or direction of transmitted motion | 0.51 | 1.00 | 0.02 |  |  |
|  |  |  |  | 2nd | equipment and supplies for a particular operation, sport | 0.49 |  |  |  |  |
| **GERMAN** | H(+P) | 93 | 135 | 1st | people/culture | 0.67 | 1.00 | 0.50 | P | 2nd WS Homonym def is obscure (having same parents) |
|  |  |  |  | 2nd | language | 0.33 |  |  |  |  |
| **GLARE** | H(+P) | 93 | 181 | 1st | stare angrily | 0.67 | 1.00 | 0.50 |  |  |
|  |  |  |  | 2nd | dazzling light or brilliance | 0.33 |  |  |  |  |
| **GLASSES** | H(+P) | 189 | 342 | 1st | a pair of lenses for correcting faulty vision | 0.94 | 1.00 | 0.94 |  | plural of glass vs. eyeglasses |
|  |  |  |  | 2nd | drinking glasses | 0.06 |  |  |  |  |
| **GOAL** | P | 96 | 183 | 1st | aim or object towards which an endeavor is directed | 0.55 | 1.00 | 0.17 |  |  |
|  |  |  |  | 2nd | net, basket, etc., into or over which players try to propel the ball, puck, etc., to score | 0.45 |  |  |  |  |
| **GOLD** | P | 93 | 161 | 1st | dense inert bright yellow element/metal | 0.91 | 1.00 | 0.90 |  |  |
|  |  |  |  | 2nd | deep yellow color | 0.09 |  |  |  |  |
| **GRACE** | P | 93 | 144 | 1st | elegance and beauty of movement, form, expression, or proportion | 0.59 | 0.92 | 0.45 |  |  |
|  |  |  |  | 2nd | short prayer | 0.33 |  |  |  |  |
|  |  |  |  | 3rd | name | 0.08 |  |  |  |  |
| **GRAIN** | P | 94 | 173 | 1st | small hard seedlike fruit of a grass, esp. a cereal plant | 0.90 | 1.00 | 0.88 |  |  |
|  |  |  |  | 2nd | small hard particle, e.g., sand | 0.10 |  |  |  |  |
| **GRAVE** | H(+P) | 96 | 80 | 1st | place for the burial of a corpse | 0.86 | 1.00 | 0.84 |  |  |
|  |  |  |  | 2nd | serious and solemn | 0.14 |  |  |  |  |
|  |  |  |  | 3rd | full of or suggesting danger | 0.00 |  |  |  |  |
| **GROWTH** | P | 93 | 176 | 1st | process or act of growing | 0.62 | 1.00 | 0.39 |  |  |
|  |  |  |  | 2nd | something grown or growing | 0.38 |  |  |  |  |
| **GUIDE** | P | 94 | 121 | 1st | a person, animal, or thing that guides | 0.74 | 1.00 | 0.64 |  |  |
|  |  |  |  | 2nd | a book (map) that instructs or explains | 0.26 |  |  |  |  |
| **HAIL** | H(+P) | 94 | 88 | 1st | small pellets of ice falling from cumulonimbus clouds | 0.67 | 0.98 | 0.54 |  |  |
|  |  |  |  | 2nd | greet/respect/honor | 0.31 |  |  |  |  |
|  |  |  |  | 3rd | flag down (cab) | 0.02 |  |  |  |  |
| **HAIR** | P | 94 | 140 | 1st | collective hairs | 0.94 | 1.00 | 0.94 |  |  |
|  |  |  |  | 2nd | single hair | 0.06 |  |  |  |  |
| **HALL** | P | 93 | 140 | 1st | large building or room | 0.66 | 0.84 | 0.73 |  |  |
|  |  |  |  | 2nd | corridor | 0.18 |  |  |  |  |
|  |  |  |  | 3rd | residential building, esp. in a university; hall of residence | 0.16 |  |  |  |  |
| **HAND** | P | 94 | 177 | 1st | part of the body at the end of the arm, consisting of a thumb, four fingers, and a palm | 1.00 | 1.00 | 1.00 |  |  |
|  |  |  |  | 2nd | cards dealt to one or all players | 0.00 |  |  |  |  |
| **HANG** | P | 93 | 135 | 1st | to fasten or be fastened from above, esp. by a cord, chain, etc.; suspend | 0.59 | 0.86 | 0.55 |  |  |
|  |  |  |  | 2nd | suspend or be suspended by the neck until dead | 0.27 |  |  |  |  |
|  |  |  |  | 3rd | hang out | 0.14 |  |  |  |  |
| **HARD** | P | 95 | 177 | 1st | firm or rigid | 0.77 | 1.00 | 0.71 |  |  |
|  |  |  |  | 2nd | difficult | 0.23 |  |  |  |  |
| **HAWAII** | P | 191 | 260 | 1st | island | 0.89 | 1.00 | 0.88 |  |  |
|  |  |  |  | 2nd | US state | 0.11 |  |  |  |  |
| **HAZE** | H(+P) | 90 | 75 | 1st | reduced visibility in the air | 0.44 | 0.77 | 0.24 |  |  |
|  |  |  |  | 2nd | subject (fellow students) to ridicule or abuse | 0.33 |  |  |  |  |
|  |  |  |  | 3rd | confusion/mental state | 0.23 |  |  |  |  |
| **HEART** | P | 191 | 355 | 1st | muscular organ in vertebrates whose contractions propel the blood through the circulatory system | 0.63 | 0.88 | 0.60 |  |  |
|  |  |  |  | 2nd | representation of the heart, having two rounded lobes at the top meeting in a point at the bottom | 0.25 |  |  |  |  |
|  |  |  |  | 3rd | symbol of love/strong feeling | 0.12 |  |  |  |  |
| **HEMINGWAY** | P | 93 | 162 | 1st | novelist/author | 0.64 | 1.00 | 0.44 |  |  |
|  |  |  |  | 2nd | Hemingway's writing: books, novels, literature | 0.36 |  |  |  |  |
| **HIP-HOP** | P | 94 | 154 | 1st | music | 0.64 | 1.00 | 0.43 |  |  |
|  |  |  |  | 2nd | dance | 0.36 |  |  |  |  |
| **HIROSHIMA** | P | 187 | 351 | 1st | site of first atomic bomb to be used in warfare | 0.63 | 1.00 | 0.42 |  |  |
|  |  |  |  | 2nd | city in Japan | 0.37 |  |  |  |  |
| **HOLLYWOOD** | P | 94 | 174 | 1st | the American film industry | 0.68 | 1.00 | 0.53 |  |  |
|  |  |  |  | 2nd | suburb of Los Angeles | 0.32 |  |  |  |  |
| **HONDA** | NL | 93 | 175 | 1st | car | 0.83 | 1.00 | 0.79 | P | Car brand name is not listed in WS dictionary |
|  |  |  |  | 2nd | automobile brand/manufacturer/corporation | 0.17 |  |  |  |  |
| **HONEY** | P | 96 | 186 | 1st | sweet, sticky, yellowish substance that is made by bees | 0.95 | 1.00 | 0.94 |  |  |
|  |  |  |  | 2nd | You call someone honey as a sign of affection | 0.05 |  |  |  |  |
| **HOOD** | P | 96 | 157 | 1st | loose head covering | 0.54 | 0.86 | 0.41 |  |  |
|  |  |  |  | 2nd | short for neighborhood | 0.32 |  |  |  |  |
|  |  |  |  | 3rd | metal cover over the car engine | 0.14 |  |  |  |  |
| **HORN** | P | 94 | 146 | 1st | musical instrument | 0.38 | 0.70 | 0.15 |  |  |
|  |  |  |  | 2nd | device on an automobile for making a warning noise | 0.32 |  |  |  |  |
|  |  |  |  | 3rd | animal bone | 0.30 |  |  |  |  |
| **HORSESHOES** | P | 93 | 161 | 1st | U-shaped plate nailed to underside of horse's hoof | 0.70 | 0.90 | 0.71 |  |  |
|  |  |  |  | 2nd | outdoor game | 0.20 |  |  |  |  |
|  |  |  |  | 3rd | lucky charm | 0.10 |  |  |  |  |
| **HOSPITAL** | P | 94 | 181 | 1st | medical institution | 0.50 | 1.00 | 0.01 |  |  |
|  |  |  |  | 2nd | health facility, building | 0.50 |  |  |  |  |
| **HUSKY** | H(+P) | 95 | 79 | 1st | Eskimo dog | 0.78 | 0.92 | 0.82 |  |  |
|  |  |  |  | 2nd | muscular and heavily built | 0.14 |  |  |  |  |
|  |  |  |  | 3rd | voice | 0.08 |  |  |  |  |
| **IMPRESSION** | P | 93 | 74 | 1st | feeling, belief, notion, opinion | 0.74 | 0.88 | 0.82 |  |  |
|  |  |  |  | 2nd | imitation | 0.14 |  |  |  |  |
|  |  |  |  | 3rd | indentation | 0.12 |  |  |  |  |
| **INTEREST** | P | 95 | 175 | 1st | a sense of concern with and curiosity about someone or something | 0.86 | 1.00 | 0.83 |  |  |
|  |  |  |  | 2nd | a fixed charge for borrowing money | 0.14 |  |  |  |  |
| **INTRODUCTION** | P | 93 | 163 | 1st | a preliminary part, as of a book, speech | 0.46 | 0.87 | 0.12 |  |  |
|  |  |  |  | 2nd | presentation of one person to another or others | 0.40 |  |  |  |  |
|  |  |  |  | 3rd | beginning level | 0.13 |  |  |  |  |
| **IRAQ** | P | 93 | 159 | 1st | Gulf War | 0.67 | 1.00 | 0.51 |  |  |
|  |  |  |  | 2nd | Persian Gulf country | 0.33 |  |  |  |  |
| **IRON** | P | 189 | 347 | 1st | metallic element | 0.68 | 1.00 | 0.52 |  |  |
|  |  |  |  | 2nd | home appliance used to smooth cloth | 0.32 |  |  |  |  |
| **ISSUE** | P | 191 | 324 | 1st | important question that is in dispute and must be settled | 0.69 | 1.00 | 0.54 |  |  |
|  |  |  |  | 2nd | one of a series published periodically | 0.31 |  |  |  |  |
| **IVORY** | P | 92 | 162 | 1st | elephant tusk | 0.73 | 1.00 | 0.64 |  |  |
|  |  |  |  | 2nd | off-white color | 0.27 |  |  |  |  |
| **JADE** | H(+P) | 93 | 137 | 1st | semiprecious gemstone | 0.61 | 1.00 | 0.35 | P | 2nd Homonym def is infrequent (worn out through over-exposure) |
|  |  |  |  | 2nd | green color | 0.39 |  |  |  |  |
| **JAM** | H(+P) | 93 | 172 | 1st | preserve of crushed fruit | 0.77 | 1.00 | 0.71 |  |  |
|  |  |  |  | 2nd | improvise musically | 0.23 |  |  |  |  |
| **JAPANESE** | P | 95 | 162 | 1st | people/culture | 0.48 | 0.83 | 0.27 |  |  |
|  |  |  |  | 2nd | food | 0.35 |  |  |  |  |
|  |  |  |  | 3rd | language | 0.17 |  |  |  |  |
| **JERK** | H(+P) | 93 | 156 | 1st | stupid fatuous person | 0.79 | 0.96 | 0.79 |  |  |
|  |  |  |  | 2nd | meat (especially beef) cut in strips | 0.17 |  |  |  |  |
|  |  |  |  | 3rd | pull/twitch | 0.04 |  |  |  |  |
| **JINGLE** | P | 95 | 183 | 1st | a metallic sound | 0.69 | 1.00 | 0.55 |  |  |
|  |  |  |  | 2nd | catchy and rhythmic verse, song, etc., esp. one used in advertising | 0.31 |  |  |  |  |
| **JOINT** | P | 94 | 170 | 1st | the point of connection between two bones | 0.70 | 0.92 | 0.68 |  |  |
|  |  |  |  | 2nd | marijuana cigarette | 0.22 |  |  |  |  |
|  |  |  |  | 3rd | hangout place | 0.02 |  |  |  |  |
|  |  |  |  | 4th | something joined/shared | 0.05 |  |  |  |  |
| **JOKER** | P | 94 | 182 | 1st | character in Batman | 0.55 | 0.87 | 0.44 |  |  |
|  |  |  |  | 2nd | playing card | 0.31 |  |  |  |  |
|  |  |  |  | 3rd | person who enjoys telling or playing jokes | 0.13 |  |  |  |  |
| **JUG** | P | 95 | 167 | 1st | what's contained in a jug | 0.69 | 0.98 | 0.59 |  |  |
|  |  |  |  | 2nd | a large bottle with a narrow mouth | 0.29 |  |  |  |  |
|  |  |  |  | 3rd | breasts | 0.02 |  |  |  |  |
| **JUNIOR** | P | 96 | 153 | 1st | a third-year undergraduate/high schooler | 0.67 | 1.00 | 0.50 |  |  |
|  |  |  |  | 2nd | the younger of two persons | 0.33 |  |  |  |  |
| **KEG** | P | 89 | 160 | 1st | quantity contained in a keg | 0.61 | 1.00 | 0.35 |  |  |
|  |  |  |  | 2nd | small cask or barrel | 0.39 |  |  |  |  |
| **KITTY** | H(+P) | 92 | 175 | 1st | domestic cat | 1.00 | 1.00 | 1.00 |  |  |
|  |  |  |  | 2nd | combined stakes of the betters | 0.00 |  |  |  |  |
| **KNEES** | P | 96 | 191 | 1st | hinge joint in the human leg | 0.99 | 1.00 | 0.99 |  |  |
|  |  |  |  | 2nd | part of a trouser leg | 0.01 |  |  |  |  |
| **KOREAN** | P | 95 | 169 | 1st | person/culture | 0.73 | 0.93 | 0.73 |  |  |
|  |  |  |  | 2nd | food | 0.20 |  |  |  |  |
|  |  |  |  | 3rd | language | 0.07 |  |  |  |  |
| **LABOR** | P | 189 | 353 | 1st | physical work done for wages | 0.81 | 1.00 | 0.77 |  |  |
|  |  |  |  | 2nd | concluding state of pregnancy | 0.19 |  |  |  |  |
| **LACE** | P | 91 | 152 | 1st | delicate decorative fabric woven in an open web of symmetrical patterns | 0.66 | 1.00 | 0.50 |  |  |
|  |  |  |  | 2nd | a cord that is drawn through eyelets or around hooks in order to draw together two edges (as of a shoe or garment) | 0.34 |  |  |  |  |
| **LAMB** | P | 94 | 181 | 1st | young sheep | 0.75 | 1.00 | 0.67 |  |  |
|  |  |  |  | 2nd | flesh of a young domestic sheep eaten as food | 0.25 |  |  |  |  |
| **LAP** | H(+P) | 95 | 176 | 1st | upper side of the thighs of a seated person | 0.69 | 1.00 | 0.56 |  |  |
|  |  |  |  | 2nd | movement once around a course | 0.31 |  |  |  |  |
| **LAVENDER** | P | 96 | 182 | 1st | aromatic shrubs | 0.63 | 1.00 | 0.40 |  |  |
|  |  |  |  | 2nd | pale purple color | 0.37 |  |  |  |  |
| **LEAD** | H(+P) | 95 | 181 | 1st | an advantage held by a competitor in a race | 0.41 | 0.73 | 0.22 |  |  |
|  |  |  |  | 2nd | the marking substance in a pencil | 0.32 |  |  |  |  |
|  |  |  |  | 3rd | toxic malleable metallic element | 0.27 |  |  |  |  |
| **LEAN** | H(+P) | 187 | 175 | 1st | lacking excess flesh | 0.63 | 1.00 | 0.42 |  |  |
|  |  |  |  | 2nd | incline or bend from a vertical position | 0.37 |  |  |  |  |
| **LEGS** | P | 96 | 93 | 1st | human limb | 0.97 | 1.00 | 0.97 |  |  |
|  |  |  |  | 2nd | supports for a piece of furniture | 0.03 |  |  |  |  |
| **LEMON** | P | 191 | 359 | 1st | yellow oval fruit | 0.99 | 1.00 | 0.99 |  |  |
|  |  |  |  | 2nd | defective auto | 0.01 |  |  |  |  |
| **LETTER** | P | 96 | 184 | 1st | written message addressed to a person | 0.78 | 1.00 | 0.71 |  |  |
|  |  |  |  | 2nd | characters of the alphabet | 0.22 |  |  |  |  |
| **LIBRARY** | P | 93 | 182 | 1st | collection of literary documents or records kept for reference or borrowing | 0.59 | 1.00 | 0.31 |  |  |
|  |  |  |  | 2nd | building that houses a collection of books | 0.41 |  |  |  |  |
| **LIMB** | H(+P) | 190 | 328 | 1st | appendages of an animal used for locomotion or grasping: arm; leg; wing; flipper | 0.93 | 1.00 | 0.92 | P | 2nd WS Homonym def is obscure (edge/border) |
|  |  |  |  | 2nd | branches arising from a tree | 0.07 |  |  |  |  |
| **LIMP** | H(+P) | 95 | 93 | 1st | uneven manner of walking | 0.72 | 1.00 | 0.61 |  |  |
|  |  |  |  | 2nd | without energy or will | 0.28 |  |  |  |  |
| **LINCOLN** | H(+P) | 95 | 163 | 1st | president | 0.90 | 0.95 | 0.95 |  | Car brand name is not listed in WS dictionary |
|  |  |  |  | 2nd | car | 0.05 |  |  |  |  |
|  |  |  |  | 3rd | something named after Lincoln | 0.05 |  |  |  |  |
| **LINE** | H(+P) | 188 | 284 | 1st | mark that is long relative to its width | 0.55 | 0.94 | 0.28 | P | 2nd WS Homonym def (e.g., line the inside of) not in top 2 norming responses |
|  |  |  |  | 2nd | formation of people or things one beside another | 0.39 |  |  |  |  |
|  |  |  |  | 3rd | something in shape of line | 0.06 |  |  |  |  |
| **LIVER** | H(+P) | 94 | 169 | 1st | bodily organ | 0.86 | 1.00 | 0.84 | P | Other homonym defs infrequent (one who lives; comparative of live) |
|  |  |  |  | 2nd | liver of an animal used as meat | 0.14 |  |  |  |  |
| **LOBSTER** | P | 93 | 141 | 1st | edible flesh of a lobster | 0.51 | 1.00 | 0.04 |  |  |
|  |  |  |  | 2nd | marine crustaceans | 0.49 |  |  |  |  |
| **LOCK** | H(+P) | 96 | 188 | 1st | fastener fitted to a door or drawer to keep it firmly closed | 0.98 | 1.00 | 0.98 |  |  |
|  |  |  |  | 2nd | strand or cluster of hair | 0.02 |  |  |  |  |
| **LOG** | H(+P) | 95 | 164 | 1st | segment of the trunk of a tree | 0.73 | 0.91 | 0.76 |  |  |
|  |  |  |  | 2nd | record | 0.18 |  |  |  |  |
|  |  |  |  | 3rd | mathematical, exponent required to produce a given number | 0.09 |  |  |  |  |
| **LORD** | P | 96 | 179 | 1st | god | 0.51 | 1.00 | 0.05 |  |  |
|  |  |  |  | 2nd | nobleman | 0.49 |  |  |  |  |
| **MADONNA** | P | 189 | 346 | 1st | pop star | 0.99 | 1.00 | 0.99 |  |  |
|  |  |  |  | 2nd | Virgin Mary | 0.01 |  |  |  |  |
| **MAGAZINE** | P | 93 | 185 | 1st | a periodic publication | 0.92 | 1.00 | 0.92 |  |  |
|  |  |  |  | 2nd | business firm that publishes magazines | 0.08 |  |  |  |  |
| **MAIL** | H(+P) | 96 | 188 | 1st | letters and packages that are transported by the postal service | 0.99 | 1.00 | 0.99 |  |  |
|  |  |  |  | 2nd | flexible armor | 0.01 |  |  |  |  |
| **MAJOR** | P | 94 | 155 | 1st | principal field of study of a student at a university | 0.70 | 0.91 | 0.71 |  |  |
|  |  |  |  | 2nd | important, serious, significant | 0.21 |  |  |  |  |
|  |  |  |  | 3rd | commissioned military officer | 0.09 |  |  |  |  |
| **MAN** | P | 93 | 172 | 1st | adult male | 0.85 | 1.00 | 0.82 |  |  |
|  |  |  |  | 2nd | human being | 0.15 |  |  |  |  |
| **MAPLE** | P | 93 | 129 | 1st | trees or shrubs | 0.65 | 0.95 | 0.55 |  |  |
|  |  |  |  | 2nd | flavor | 0.29 |  |  |  |  |
|  |  |  |  | 3rd | wood of any of various maple trees | 0.05 |  |  |  |  |
| **MARBLE** | P | 93 | 158 | 1st | metamorphic rock that takes a high polish | 0.58 | 1.00 | 0.28 |  |  |
|  |  |  |  | 2nd | small ball of glass | 0.42 |  |  |  |  |
| **MARCH** | H(+P) | 96 | 186 | 1st | month | 0.67 | 0.98 | 0.52 |  |  |
|  |  |  |  | 2nd | the act of marching | 0.32 |  |  |  |  |
|  |  |  |  | 3rd | protest | 0.02 |  |  |  |  |
| **MASS** | H(+P) | 93 | 162 | 1st | have weight in a gravitational field | 0.77 | 1.00 | 0.69 |  |  |
|  |  |  |  | 2nd | Church celebration of the Eucharist | 0.23 |  |  |  |  |
| **MATCH** | H(+P) | 96 | 184 | 1st | lighter consisting of a thin piece of wood | 0.47 | 0.88 | 0.13 |  |  |
|  |  |  |  | 2nd | person/thing that's equal | 0.41 |  |  |  |  |
|  |  |  |  | 3rd | formal contest in which two or more persons or teams compete | 0.11 |  |  |  |  |
|  |  |  |  | 4th | website | 0.02 |  |  |  |  |
| **MATERIAL** | P | 189 | 371 | 1st | stuff | 0.75 | 1.00 | 0.67 |  |  |
|  |  |  |  | 2nd | cloth, fabric | 0.25 |  |  |  |  |
| **MEDITERRANEAN** | P | 95 | 184 | 1st | sea (region) | 0.67 | 1.00 | 0.50 |  |  |
|  |  |  |  | 2nd | food | 0.33 |  |  |  |  |
| **MENU** | P | 93 | 173 | 1st | list of dishes available at a restaurant | 0.97 | 1.00 | 0.96 |  |  |
|  |  |  |  | 2nd | list of options available to a computer user | 0.03 |  |  |  |  |
| **MEXICAN** | P | 93 | 173 | 1st | native or inhabitant of Mexico | 0.55 | 1.00 | 0.18 |  |  |
|  |  |  |  | 2nd | food | 0.45 |  |  |  |  |
| **MIGHT** | H(+P) | 188 | 173 | 1st | possibility | 0.51 | 1.00 | 0.03 |  |  |
|  |  |  |  | 2nd | power/physical strength | 0.49 |  |  |  |  |
| **MILK** | P | 189 | 359 | 1st | nutritious liquid secreted by mammals | 0.81 | 1.00 | 0.76 |  |  |
|  |  |  |  | 2nd | take milk from female mammals | 0.19 |  |  |  |  |
| **MINE** | H(+P) | 93 | 178 | 1st | excavation in the earth | 0.53 | 1.00 | 0.11 |  |  |
|  |  |  |  | 2nd | showing possession | 0.47 |  |  |  |  |
| **MINT** | H(+P) | 192 | 356 | 1st | leaves of a mint plant used fresh or candied | 0.96 | 1.00 | 0.96 |  |  |
|  |  |  |  | 2nd | plant where money is coined by authority of the government | 0.04 |  |  |  |  |
|  |  |  |  | 3rd | candy | 0.00 |  |  |  |  |
| **MISS** | H(+P) | 96 | 180 | 1st | (fail to perceive or to catch with the senses or the mind | 0.55 | 1.00 | 0.18 |  |  |
|  |  |  |  | 2nd | a young female | 0.45 |  |  |  |  |
| **MISSISSIPPI** | P | 95 | 140 | 1st | state | 0.51 | 1.00 | 0.03 |  |  |
|  |  |  |  | 2nd | river | 0.49 |  |  |  |  |
| **MODEL** | P | 93 | 166 | 1st | fashion model | 0.74 | 0.92 | 0.76 |  |  |
|  |  |  |  | 2nd | type of product, e.g., car model | 0.18 |  |  |  |  |
|  |  |  |  | 3rd | representation | 0.08 |  |  |  |  |
| **MOLD** | H(+P) | 94 | 88 | 1st | a fungus | 0.83 | 1.00 | 0.79 |  |  |
|  |  |  |  | 2nd | shape, influence | 0.17 |  |  |  |  |
| **MOLE** | H(+P) | 96 | 148 | 1st | burrowing mammal | 0.51 | 0.97 | 0.12 |  |  |
|  |  |  |  | 2nd | pigmented spot on the skin | 0.45 |  |  |  |  |
|  |  |  |  | 3rd | chemistry | 0.03 |  |  |  |  |
| **MONITOR** | P | 190 | 172 | 1st | computer screen | 0.65 | 1.00 | 0.45 |  |  |
|  |  |  |  | 2nd | keep tabs on | 0.35 |  |  |  |  |
| **MOUTH** | P | 96 | 189 | 1st | oral cavity, opening | 0.78 | 1.00 | 0.71 |  |  |
|  |  |  |  | 2nd | the externally visible part of the oral cavity on the face and the system of organs surrounding the opening | 0.22 |  |  |  |  |
|  |  |  |  | 3rd | point where a stream issues into a larger body of water | 0.00 |  |  |  |  |
| **MOZART** | P | 94 | 153 | 1st | music | 0.65 | 1.00 | 0.47 |  |  |
|  |  |  |  | 2nd | composer | 0.35 |  |  |  |  |
| **MUSIC** | P | 93 | 88 | 1st | sounds produced by singers or musical instruments | 0.61 | 1.00 | 0.37 |  |  |
|  |  |  |  | 2nd | artistic form of auditory communication | 0.39 |  |  |  |  |
| **MUSTANG** | NL | 94 | 172 | 1st | car | 0.65 | 1.00 | 0.46 | ? | Car name not listed in WS dictionary |
|  |  |  |  | 2nd | horse | 0.35 |  |  |  |  |
| **NAIL** | P | 96 | 182 | 1st | finger/toe | 0.70 | 1.00 | 0.58 |  |  |
|  |  |  |  | 2nd | thin pointed piece of metal that is hammered | 0.30 |  |  |  |  |
| **NAVY** | P | 95 | 179 | 1st | organization of military | 0.76 | 1.00 | 0.68 |  |  |
|  |  |  |  | 2nd | dark shade of blue | 0.24 |  |  |  |  |
| **NECK** | P | 96 | 180 | 1st | connects the head to the rest of the body | 0.96 | 0.99 | 0.96 |  |  |
|  |  |  |  | 2nd | opening in a garment for the neck of the wearer | 0.04 |  |  |  |  |
|  |  |  |  | 3rd | narrow part of something | 0.01 |  |  |  |  |
| **NEWSPAPER** | P | 191 | 327 | 1st | daily or weekly publication | 0.95 | 1.00 | 0.95 |  |  |
|  |  |  |  | 2nd | a business firm that publishes newspapers | 0.05 |  |  |  |  |
| **NIRVANA** | P | 93 | 178 | 1st | Seattle band | 0.54 | 1.00 | 0.16 | ? | Band name is not listed in WS dictionary |
|  |  |  |  | 2nd | enlightenment, bliss | 0.46 |  |  |  |  |
| **NOODLE** | H | 96 | 178 | 1st | strip of pasta | 0.93 | 1.00 | 0.93 |  |  |
|  |  |  |  | 2nd | pool floatie | 0.07 |  |  |  |  |
| **NOSE** | P | 96 | 187 | 1st | prominent part of the face of man or other mammals | 0.76 | 1.00 | 0.68 |  |  |
|  |  |  |  | 2nd | the sense of smell | 0.24 |  |  |  |  |
| **NOTE** | P | 96 | 187 | 1st | brief written record | 0.96 | 1.00 | 0.96 |  |  |
|  |  |  |  | 2nd | musical notation | 0.04 |  |  |  |  |
| **NOVEL** | H | 93 | 90 | 1st | extended fictional work | 0.84 | 1.00 | 0.82 |  |  |
|  |  |  |  | 2nd | pleasantly new or different | 0.16 |  |  |  |  |
| **NUT** | P | 94 | 170 | 1st | hard-shelled seed | 0.92 | 1.00 | 0.92 |  |  |
|  |  |  |  | 2nd | freak/crazy person | 0.08 |  |  |  |  |
| **OAK** | P | 92 | 159 | 1st | deciduous tree | 0.65 | 1.00 | 0.47 |  |  |
|  |  |  |  | 2nd | hard durable wood | 0.35 |  |  |  |  |
| **ODD** | P | 92 | 170 | 1st | peculiar | 0.66 | 1.00 | 0.48 |  |  |
|  |  |  |  | 2nd | not divisible by two | 0.34 |  |  |  |  |
| **OIL** | P | 96 | 162 | 1st | petroleum, dark oil consisting mainly of hydrocarbons | 0.62 | 1.00 | 0.38 |  |  |
|  |  |  |  | 2nd | liquid (edible) fats that are obtained from plants | 0.38 |  |  |  |  |
| **OLIVE** | P | 96 | 171 | 1st | small ovoid fruit | 0.94 | 1.00 | 0.93 |  |  |
|  |  |  |  | 2nd | yellow-green color | 0.06 |  |  |  |  |
| **OPENING** | P | 95 | 174 | 1st | the act of opening something | 0.56 | 1.00 | 0.21 |  |  |
|  |  |  |  | 2nd | ceremony accompanying the start of some enterprise | 0.44 |  |  |  |  |
| **OPERATION** | P | 94 | 156 | 1st | surgery, medical procedure | 0.59 | 0.86 | 0.54 |  |  |
|  |  |  |  | 2nd | military (or business) operation | 0.27 |  |  |  |  |
|  |  |  |  | 3rd | children's board game | 0.14 |  |  |  |  |
| **ORANGE** | P | 95 | 84 | 1st | fruit | 0.68 | 1.00 | 0.53 |  |  |
|  |  |  |  | 2nd | color | 0.32 |  |  |  |  |
| **ORDER** | P | 96 | 174 | 1st | request for something to be made, supplied, or served | 0.52 | 0.80 | 0.44 |  |  |
|  |  |  |  | 2nd | command given by a superior | 0.29 |  |  |  |  |
|  |  |  |  | 3rd | organization | 0.20 |  |  |  |  |
| **ORGAN** | P | 96 | 187 | 1st | biological/functional unit in an animal that is specialized | 0.75 | 1.00 | 0.67 |  |  |
|  |  |  |  | 2nd | musical instrument | 0.25 |  |  |  |  |
| **PALACE** | P | 96 | 162 | 1st | official residence/stately mansion | 0.53 | 1.00 | 0.12 |  |  |
|  |  |  |  | 2nd | governing group of a kingdom | 0.47 |  |  |  |  |
| **PALM** | H(+P) | 93 | 181 | 1st | surface of the hand | 0.54 | 1.00 | 0.15 |  |  |
|  |  |  |  | 2nd | tree | 0.46 |  |  |  |  |
| **PANEL** | P | 95 | 178 | 1st | committee appointed to judge a competition | 0.72 | 1.00 | 0.61 |  |  |
|  |  |  |  | 2nd | sheet that forms a distinct section | 0.28 |  |  |  |  |
| **PAPER** | P | 93 | 182 | 1st | material made of cellulose pulp | 0.82 | 1.00 | 0.78 |  |  |
|  |  |  |  | 2nd | an essay | 0.18 |  |  |  |  |
| **PARK** | P | 191 | 362 | 1st | area of land preserved in its natural state as public property | 0.81 | 1.00 | 0.76 |  |  |
|  |  |  |  | 2nd | place temporarily, e.g., parking space | 0.19 |  |  |  |  |
| **PART** | P | 93 | 174 | 1st | piece | 0.85 | 1.00 | 0.82 |  |  |
|  |  |  |  | 2nd | line of scalp when hair is combed | 0.15 |  |  |  |  |
| **PARTY** | P | 190 | 178 | 1st | people gathered for pleasure | 0.98 | 1.00 | 0.98 |  |  |
|  |  |  |  | 2nd | political party | 0.02 |  |  |  |  |
| **PASS** | P | 96 | 108 | 1st | success in satisfying a test or requirement | 0.64 | 0.85 | 0.67 |  |  |
|  |  |  |  | 2nd | a play that involves getting the ball to a teammate | 0.21 |  |  |  |  |
|  |  |  |  | 3rd | through way | 0.15 |  |  |  |  |
| **PASSAGE** | P | 191 | 363 | 1st | a way through or along which someone or something may pass | 0.51 | 0.83 | 0.37 |  |  |
|  |  |  |  | 2nd | section of text | 0.32 |  |  |  |  |
|  |  |  |  | 3rd | journey usually by ship | 0.17 |  |  |  |  |
| **PAWN** | H(+P) | 92 | 153 | 1st | chess piece | 0.71 | 1.00 | 0.58 |  |  |
|  |  |  |  | 2nd | an article deposited as security | 0.29 |  |  |  |  |
| **PEACH** | P | 96 | 160 | 1st | fruit | 0.85 | 1.00 | 0.82 |  |  |
|  |  |  |  | 2nd | shade of pink | 0.15 |  |  |  |  |
| **PEARL HARBOR** | P | 189 | 354 | 1st | naval base that was attacked by the Japanese | 0.83 | 1.00 | 0.80 |  |  |
|  |  |  |  | 2nd | a harbor on Oahu to the west of Honolulu | 0.17 |  |  |  |  |
| **PEEP** | H(+P) | 94 | 175 | 1st | a secret look | 0.54 | 0.86 | 0.41 |  |  |
|  |  |  |  | 2nd | weak cry of a young bird | 0.32 |  |  |  |  |
|  |  |  |  | 3rd | marshmallow treat | 0.14 |  |  |  |  |
| **PEER** | H(+P) | 192 | 178 | 1st | person who is of equal standing, an equal | 0.86 | 1.00 | 0.84 |  |  |
|  |  |  |  | 2nd | look searchingly | 0.14 |  |  |  |  |
| **PEN** | H(+P) | 96 | 186 | 1st | writing implement | 1.00 | 1.00 | 1.00 |  |  |
|  |  |  |  | 2nd | enclosure for confining livestock | 0.00 |  |  |  |  |
| **PENTAGON** | P | 93 | 184 | 1st | United States military establishment | 0.52 | 1.00 | 0.08 |  |  |
|  |  |  |  | 2nd | five-sided polygon | 0.48 |  |  |  |  |
| **PERCH** | H(+P) | 90 | 150 | 1st | resting place, especially for a bird | 0.96 | 1.00 | 0.96 |  |  |
|  |  |  |  | 2nd | fish | 0.04 |  |  |  |  |
| **PET** | P | 96 | 187 | 1st | domesticated animal | 0.95 | 0.99 | 0.95 | H | 2nd WS Homonym def is obscure (building material); Norming Def 2 is acronym and not listed in WS dictionary |
|  |  |  |  | 2nd | a computerized radiographic technique | 0.05 |  |  |  |  |
|  |  |  |  | 3rd | stroke or caress gently | 0.01 |  |  |  |  |
| **PICASSO** | P | 96 | 149 | 1st | work of the artist Picasso | 0.68 | 1.00 | 0.52 |  |  |
|  |  |  |  | 2nd | influential Spanish artist | 0.32 |  |  |  |  |
| **PICKET** | P | 91 | 74 | 1st | part of a fence | 0.76 | 1.00 | 0.68 |  |  |
|  |  |  |  | 2nd | protest posted by a labor organization | 0.24 |  |  |  |  |
| **PILL** | P | 95 | 160 | 1st | tablet of medicine | 0.90 | 1.00 | 0.89 |  |  |
|  |  |  |  | 2nd | birth control | 0.10 |  |  |  |  |
| **PINE** | H(+P) | 93 | 166 | 1st | coniferous tree | 0.89 | 0.95 | 0.93 |  |  |
|  |  |  |  | 2nd | yellowish timber | 0.06 |  |  |  |  |
|  |  |  |  | 3rd | scent | 0.05 |  |  |  |  |
| **PIPE** | H(+P) | 96 | 168 | 1st | tube made of metal or plastic that is used to carry water or oil or gas | 0.74 | 1.00 | 0.66 | P | 2nd WS Homonym def is obscure (unit of capacity) |
|  |  |  |  | 2nd | tobacco pipe | 0.26 |  |  |  |  |
| **PIT** | H(+P) | 93 | 159 | 1st | sizeable hole | 0.83 | 1.00 | 0.80 |  |  |
|  |  |  |  | 2nd | inner "stone" of some fruits | 0.17 |  |  |  |  |
| **PITCH** | H(+P) | 92 | 172 | 1st | act of throwing a baseball | 0.52 | 0.94 | 0.18 |  |  |
|  |  |  |  | 2nd | property of sound that varies with variation in the frequency | 0.42 |  |  |  |  |
|  |  |  |  | 3rd | sales pitch/promotion | 0.06 |  |  |  |  |
| **PITCHER** | H(+P) | 92 | 178 | 1st | baseball, the person who does the pitching | 0.50 | 1.00 | 0.00 |  |  |
|  |  |  |  | 2nd | vessel with a handle and a spout for pouring | 0.50 |  |  |  |  |
| **PLANE** | H(+P) | 96 | 182 | 1st | aircraft | 0.95 | 1.00 | 0.94 |  | Airplane definition not an entry |
|  |  |  |  | 2nd | mathematics: an unbounded two-dimensional shape | 0.05 |  |  |  |  |
| **PLANT** | P | 189 | 347 | 1st | botany: a living organism | 0.95 | 1.00 | 0.95 |  |  |
|  |  |  |  | 2nd | buildings for carrying on industrial labor | 0.05 |  |  |  |  |
| **PLASTIC** | P | 95 | 173 | 1st | synthetic or semisynthetic materials | 0.99 | 1.00 | 0.99 |  |  |
|  |  |  |  | 2nd | credit card | 0.01 |  |  |  |  |
| **PLATE** | P | 96 | 172 | 1st | dish on which food is served | 0.99 | 1.00 | 0.99 |  |  |
|  |  |  |  | 2nd | baseball base | 0.01 |  |  |  |  |
| **PLAY** | P | 192 | 356 | 1st | participate in games or sport | 0.74 | 0.92 | 0.74 |  |  |
|  |  |  |  | 2nd | dramatic work | 0.19 |  |  |  |  |
|  |  |  |  | 3rd | perform music | 0.08 |  |  |  |  |
| **PLOT** | H(+P) | 95 | 183 | 1st | story that is told in a novel or play or movie etc. | 0.80 | 0.92 | 0.85 |  |  |
|  |  |  |  | 2nd | a graph or visual representation of data | 0.12 |  |  |  |  |
|  |  |  |  | 3rd | a small area of ground | 0.08 |  |  |  |  |
| **PLUG** | P | 187 | 346 | 1st | electrical connection | 0.65 | 1.00 | 0.46 |  |  |
|  |  |  |  | 2nd | blockage consisting of an object designed to fill a hole tightly | 0.35 |  |  |  |  |
| **POACH** | H(+P) | 93 | 170 | 1st | hunt illegally | 0.51 | 1.00 | 0.05 |  |  |
|  |  |  |  | 2nd | cook in a simmering liquid | 0.49 |  |  |  |  |
| **POINT** | P | 91 | 70 | 1st | sharp end | 0.57 | 1.00 | 0.25 |  |  |
|  |  |  |  | 2nd | indicate a place, direction, person, or thing | 0.43 |  |  |  |  |
| **POKER** | H | 189 | 372 | 1st | card game | 0.99 | 1.00 | 0.99 |  |  |
|  |  |  |  | 2nd | metal rod with a handle | 0.01 |  |  |  |  |
| **POOL** | H(+P) | 96 | 175 | 1st | excavation filled with water | 0.92 | 1.00 | 0.91 |  |  |
|  |  |  |  | 2nd | billiards | 0.08 |  |  |  |  |
| **PORT** | H(+P) | 92 | 173 | 1st | seaport or airport | 0.83 | 0.98 | 0.82 |  |  |
|  |  |  |  | 2nd | circuitry that links one computer device with another | 0.15 |  |  |  |  |
|  |  |  |  | 3rd | wine | 0.02 |  |  |  |  |
| **POST** | H(+P) | 96 | 171 | 1st | mail/letters | 0.58 | 0.92 | 0.41 |  |  |
|  |  |  |  | 2nd | display in a public place or for public notice | 0.34 |  |  |  |  |
|  |  |  |  | 3rd | subsequent: after | 0.08 |  |  |  |  |
| **POT** | H(+P) | 96 | 184 | 1st | cooking vessel | 0.61 | 0.89 | 0.55 |  |  |
|  |  |  |  | 2nd | marijuana | 0.28 |  |  |  |  |
|  |  |  |  | 3rd | planter | 0.11 |  |  |  |  |
| **POTATO** | P | 93 | 139 | 1st | food | 0.89 | 1.00 | 0.88 |  |  |
|  |  |  |  | 2nd | garden vegetable | 0.11 |  |  |  |  |
| **POUND** | H(+P) | 93 | 175 | 1st | 16 ounces | 0.54 | 0.76 | 0.59 |  |  |
|  |  |  |  | 2nd | basic unit of money in Great Britain | 0.22 |  |  |  |  |
|  |  |  |  | 3rd | a public enclosure for stray or unlicensed dogs | 0.08 |  |  |  |  |
|  |  |  |  | 4th | hit hard | 0.16 |  |  |  |  |
| **PRESENT** | H(+P) | 188 | 365 | 1st | period of time that is happening now | 0.59 | 0.99 | 0.32 |  |  |
|  |  |  |  | 2nd | gift | 0.40 |  |  |  |  |
|  |  |  |  | 3rd | show | 0.01 |  |  |  |  |
| **PRESS** | H(+P) | 93 | 90 | 1st | print media | 0.67 | 1.00 | 0.50 | P | 2nd WS Homonym def is obscure (military service) |
|  |  |  |  | 2nd | exert pressure or force to or upon | 0.33 |  |  |  |  |
| **PRINTER** | P | 95 | 176 | 1st | machine that prints | 0.98 | 1.00 | 0.98 |  |  |
|  |  |  |  | 2nd | someone whose occupation is printing | 0.02 |  |  |  |  |
| **PRISON** | P | 93 | 147 | 1st | building | 0.52 | 1.00 | 0.07 |  |  |
|  |  |  |  | 2nd | a correctional institution | 0.48 |  |  |  |  |
| **PROGRAM** | P | 190 | 308 | 1st | a sequence of instructions that a computer can interpret and execute | 0.53 | 0.79 | 0.52 |  |  |
|  |  |  |  | 2nd | a planned series of future events, items, or performances | 0.25 |  |  |  |  |
|  |  |  |  | 3rd | a performance/show | 0.21 |  |  |  |  |
| **PUNCH** | H(+P) | 96 | 168 | 1st | deliver a quick blow to | 0.63 | 1.00 | 0.40 |  |  |
|  |  |  |  | 2nd | iced mixed drink | 0.38 |  |  |  |  |
| **PUPIL** | H | 95 | 184 | 1st | center of the iris of the eye | 0.61 | 1.00 | 0.37 |  |  |
|  |  |  |  | 2nd | student | 0.39 |  |  |  |  |
| **PYRAMID** | P | 95 | 170 | 1st | Pyramid of Egypt | 0.74 | 1.00 | 0.64 |  |  |
|  |  |  |  | 2nd | geometrical shape | 0.26 |  |  |  |  |
| **QUACK** | H(+P) | 96 | 178 | 1st | sound of a duck | 0.81 | 1.00 | 0.77 |  |  |
|  |  |  |  | 2nd | an untrained person who pretends to be a physician | 0.19 |  |  |  |  |
| **QUEEN** | P | 93 | 180 | 1st | the wife of a king | 0.84 | 0.97 | 0.84 |  |  |
|  |  |  |  | 2nd | fertile female in a colony of social insects such as bees | 0.13 |  |  |  |  |
|  |  |  |  | 3rd | bed size | 0.03 |  |  |  |  |
| **RABBIT** | P | 191 | 363 | 1st | burrowing animals | 0.98 | 1.00 | 0.98 |  |  |
|  |  |  |  | 2nd | flesh of rabbits for food | 0.02 |  |  |  |  |
| **RACE** | H(+P) | 96 | 189 | 1st | any competition | 0.59 | 1.00 | 0.30 |  |  |
|  |  |  |  | 2nd | people of the same genetic stock | 0.41 |  |  |  |  |
| **RACKET** | H(+P) | 190 | 373 | 1st | a sports implement | 0.70 | 1.00 | 0.58 |  |  |
|  |  |  |  | 2nd | a loud and disturbing noise | 0.30 |  |  |  |  |
| **RADIO** | P | 96 | 183 | 1st | broadcasting electromagnetic waves | 0.88 | 1.00 | 0.86 |  |  |
|  |  |  |  | 2nd | electronic receiver | 0.12 |  |  |  |  |
| **RADIOHEAD** | NL | 177 | 310 | 1st | music | 0.65 | 1.00 | 0.47 |  | Band name is not listed in WS dictionary |
|  |  |  |  | 2nd | band | 0.35 |  |  |  |  |
| **RADISH** | P | 95 | 36 | 1st | edible root | 0.86 | 1.00 | 0.84 |  |  |
|  |  |  |  | 2nd | cultivated plants | 0.14 |  |  |  |  |
| **RAM** | H(+P) | 95 | 176 | 1st | male sheep | 0.60 | 0.78 | 0.71 | P | 2nd WS Homonym def is acronym (computer memory) but was not in top 2 of norming |
|  |  |  |  | 2nd | force/strike | 0.18 |  |  |  |  |
|  |  |  |  | 3rd | car | 0.15 |  |  |  |  |
|  |  |  |  | 4th | computer memory | 0.07 |  |  |  |  |
| **RANGE** | P | 94 | 161 | 1st | place for shooting/firing | 0.37 | 0.66 | 0.19 |  |  |
|  |  |  |  | 2nd | mathematical values | 0.30 |  |  |  |  |
|  |  |  |  | 3rd | Rover (car) | 0.11 |  |  |  |  |
|  |  |  |  | 4th | large area of land | 0.22 |  |  |  |  |
| **RARE** | H(+P) | 96 | 190 | 1st | uncommonness | 0.86 | 1.00 | 0.84 |  |  |
|  |  |  |  | 2nd | cooked a short time | 0.14 |  |  |  |  |
| **RASH** | H(+P) | 96 | 90 | 1st | red eruption of the skin | 0.92 | 1.00 | 0.92 |  |  |
|  |  |  |  | 2nd | foolhardy | 0.08 |  |  |  |  |
| **RATE** | H(+P) | 96 | 168 | 1st | estimate the value of) | 0.41 | 0.79 | 0.07 | P | 2nd WS Homonym def is obscure (scold) |
|  |  |  |  | 2nd | frequency relative to a time unit | 0.38 |  |  |  |  |
|  |  |  |  | 3rd | charge per unit | 0.21 |  |  |  |  |
| **RATTLE** | P | 91 | 176 | 1st | baby's toy | 0.44 | 0.89 | 0.00 |  |  |
|  |  |  |  | 2nd | sections at the end of a rattlesnake's tail | 0.44 |  |  |  |  |
|  |  |  |  | 3rd | make a rattling noise | 0.11 |  |  |  |  |
| **REACTION** | P | 93 | 164 | 1st | response | 0.59 | 1.00 | 0.31 |  |  |
|  |  |  |  | 2nd | chemical reaction | 0.41 |  |  |  |  |
| **RECORD** | P | 96 | 176 | 1st | sound recording | 0.71 | 0.88 | 0.77 |  |  |
|  |  |  |  | 2nd | he best (or worst) performance | 0.16 |  |  |  |  |
|  |  |  |  | 3rd | sum of past actions or a person or organization | 0.13 |  |  |  |  |
| **REDWOOD** | P | 90 | 163 | 1st | huge coniferous California trees | 0.92 | 1.00 | 0.91 |  |  |
|  |  |  |  | 2nd | soft reddish wood | 0.08 |  |  |  |  |
| **REEL** | H(+P) | 92 | 165 | 1st | fishing rod | 0.63 | 0.97 | 0.46 |  |  |
|  |  |  |  | 2nd | a roll of photographic film | 0.34 |  |  |  |  |
|  |  |  |  | 3rd | lose balance | 0.03 |  |  |  |  |
| **RESERVATION** | P | 95 | 169 | 1st | something reserved in advance | 0.71 | 0.98 | 0.62 |  |  |
|  |  |  |  | 2nd | a district that is reserved (Indian reservation) | 0.27 |  |  |  |  |
|  |  |  |  | 3rd | doubt | 0.02 |  |  |  |  |
| **REVIEW** | P | 96 | 179 | 1st | practice intended to polish performance or refresh the memory | 0.60 | 1.00 | 0.33 |  |  |
|  |  |  |  | 2nd | essay or article that gives a critical evaluation | 0.40 |  |  |  |  |
| **RIGHT** | P | 95 | 178 | 1st | on the right side of the body | 0.63 | 0.97 | 0.47 |  |  |
|  |  |  |  | 2nd | correct | 0.34 |  |  |  |  |
|  |  |  |  | 3rd | allowed by law | 0.02 |  |  |  |  |
|  |  |  |  | 4th | conservative | 0.01 |  |  |  |  |
| **RING** | H(+P) | 96 | 176 | 1st | jewelry | 0.80 | 0.96 | 0.79 |  |  |
|  |  |  |  | 2nd | sound of a bell ringing | 0.16 |  |  |  |  |
|  |  |  |  | 3rd | shape | 0.04 |  |  |  |  |
| **ROCK** | H(+P) | 96 | 148 | 1st | mineral | 0.64 | 0.96 | 0.49 |  |  |
|  |  |  |  | 2nd | popular music | 0.32 |  |  |  |  |
|  |  |  |  | 3rd | Dwayne Johnson | 0.04 |  |  |  |  |
| **ROD** | P | 93 | 170 | 1st | a long thin implement made of metal or wood | 0.96 | 1.00 | 0.96 |  |  |
|  |  |  |  | 2nd | visual receptor | 0.04 |  |  |  |  |
| **ROLL** | P | 92 | 84 | 1st | anything rolled up in cylindrical form | 0.44 | 0.85 | 0.08 |  |  |
|  |  |  |  | 2nd | move by turning over or rotating | 0.40 |  |  |  |  |
|  |  |  |  | 3rd | rock n roll | 0.15 |  |  |  |  |
| **ROOM** | P | 95 | 178 | 1st | an area within a building enclosed by walls and floor and ceiling | 0.90 | 1.00 | 0.89 |  |  |
|  |  |  |  | 2nd | space for movement | 0.10 |  |  |  |  |
| **ROW** | H(+P) | 95 | 82 | 1st | propel with oars | 0.63 | 1.00 | 0.42 |  |  |
|  |  |  |  | 2nd | a linear array of numbers, letters, or symbols | 0.37 |  |  |  |  |
| **RUBY** | P | 189 | 316 | 1st | precious gem | 0.57 | 1.00 | 0.24 |  |  |
|  |  |  |  | 2nd | deep red | 0.43 |  |  |  |  |
| **RULER** | P | 95 | 185 | 1st | measuring stick | 0.77 | 1.00 | 0.70 |  |  |
|  |  |  |  | 2nd | a person who rules or commands | 0.23 |  |  |  |  |
| **SALMON** | P | 96 | 192 | 1st | game fishes | 0.77 | 1.00 | 0.70 |  |  |
|  |  |  |  | 2nd | pale pinkish orange color | 0.23 |  |  |  |  |
| **SASH** | H(+P) | 92 | 172 | 1st | band of material | 1.00 | 1.00 | 1.00 |  |  |
|  |  |  |  | 2nd | window | 0.00 |  |  |  |  |
| **SAW** | H(+P) | 96 | 188 | 1st | tool for cutting | 0.45 | 0.79 | 0.25 |  |  |
|  |  |  |  | 2nd | past tense of see | 0.34 |  |  |  |  |
|  |  |  |  | 3rd | horror movie | 0.21 |  |  |  |  |
| **SCALE** | H(+P) | 93 | 177 | 1st | weighing machine | 0.75 | 0.89 | 0.82 |  |  |
|  |  |  |  | 2nd | from the surface (of the skin) | 0.14 |  |  |  |  |
|  |  |  |  | 3rd | range/size | 0.11 |  |  |  |  |
| **SCENE** | P | 191 | 364 | 1st | subdivision of a film or play | 0.58 | 1.00 | 0.27 |  |  |
|  |  |  |  | 2nd | place where some action occurs | 0.42 |  |  |  |  |
| **SCHOOL** | H(+P) | 94 | 163 | 1st | building where people receive education | 0.57 | 1.00 | 0.25 | P | 2nd WS Homonym def (e.g., school of fish) is infrequent |
|  |  |  |  | 2nd | an educational institution | 0.43 |  |  |  |  |
| **SCOOP** | P | 96 | 181 | 1st | quantity a scoop will hold | 0.63 | 0.80 | 0.73 |  |  |
|  |  |  |  | 2nd | a news report | 0.17 |  |  |  |  |
|  |  |  |  | 3rd | a large ladle | 0.12 |  |  |  |  |
|  |  |  |  | 4th | shovel | 0.08 |  |  |  |  |
| **SCOUT** | H(+P) | 93 | 81 | 1st | Boy Scout or Girl Scout | 0.69 | 0.86 | 0.75 |  |  |
|  |  |  |  | 2nd | explore, often with the goal of finding something or somebody | 0.17 |  |  |  |  |
|  |  |  |  | 3rd | person employed to keep watch for some anticipated event | 0.14 |  |  |  |  |
| **SCREEN** | P | 96 | 179 | 1st | electronic display | 0.70 | 0.88 | 0.74 |  |  |
|  |  |  |  | 2nd | protective covering | 0.18 |  |  |  |  |
|  |  |  |  | 3rd | to test | 0.12 |  |  |  |  |
| **SEAL** | H(+P) | 96 | 173 | 1st | marine mammals | 0.62 | 0.82 | 0.67 |  |  |
|  |  |  |  | 2nd | Navy seal | 0.20 |  |  |  |  |
|  |  |  |  | 3rd | document/letter fastener | 0.18 |  |  |  |  |
| **SEASON** | P | 189 | 183 | 1st | time of year | 0.91 | 1.00 | 0.90 |  |  |
|  |  |  |  | 2nd | lend flavor to | 0.09 |  |  |  |  |
| **SECOND** | H(+P) | 96 | 189 | 1st | following first | 0.61 | 0.99 | 0.37 |  |  |
|  |  |  |  | 2nd | 1/60 of a minute | 0.38 |  |  |  |  |
|  |  |  |  | 3rd | agree with | 0.01 |  |  |  |  |
| **SENSE** | P | 96 | 183 | 1st | sensation/senses | 0.87 | 1.00 | 0.85 |  |  |
|  |  |  |  | 2nd | sound practical judgment | 0.13 |  |  |  |  |
| **SENTENCE** | P | 93 | 178 | 1st | a string of words | 0.89 | 1.00 | 0.87 |  |  |
|  |  |  |  | 2nd | time a prisoner is imprisoned | 0.11 |  |  |  |  |
| **SHAKESPEARE** | P | 93 | 148 | 1st | writing/poems/plays | 0.72 | 1.00 | 0.62 |  |  |
|  |  |  |  | 2nd | writer | 0.28 |  |  |  |  |
| **SHED** | H(+P) | 96 | 89 | 1st | outbuilding | 0.52 | 0.93 | 0.20 |  |  |
|  |  |  |  | 2nd | get rid of | 0.42 |  |  |  |  |
|  |  |  |  | 3rd | spill in drops | 0.07 |  |  |  |  |
| **SHEET** | H(+P) | 93 | 169 | 1st | bed linen | 0.52 | 1.00 | 0.08 | P | 2nd WS Homonym def is obscure (sail) |
|  |  |  |  | 2nd | piece of paper | 0.48 |  |  |  |  |
| **SHEETS** | H(+P) | 94 | 179 | 1st | bed linen | 0.63 | 1.00 | 0.42 | P | 2nd WS Homonym def is obscure (sail) |
|  |  |  |  | 2nd | piece of paper | 0.37 |  |  |  |  |
| **SHOWER** | H(+P) | 190 | 309 | 1st | plumbing fixture | 0.93 | 0.99 | 0.93 | P | 2nd Homonym def is different pronunciation and infrequent (one that shows) |
|  |  |  |  | 2nd | rain/precipitation | 0.06 |  |  |  |  |
|  |  |  |  | 3rd | baby/bridal | 0.01 |  |  |  |  |
| **SIGN** | P | 93 | 173 | 1st | public display of a message | 0.45 | 0.73 | 0.35 |  |  |
|  |  |  |  | 2nd | gesture that is part of a sign language | 0.29 |  |  |  |  |
|  |  |  |  | 3rd | mark with one's signature | 0.27 |  |  |  |  |
| **SILK** | P | 95 | 142 | 1st | fabric made from fine threads | 0.89 | 1.00 | 0.88 |  |  |
|  |  |  |  | 2nd | animal fibers produced by silkworms | 0.11 |  |  |  |  |
| **SINK** | P | 93 | 154 | 1st | plumbing fixture | 0.71 | 1.00 | 0.60 |  |  |
|  |  |  |  | 2nd | fall or descend | 0.29 |  |  |  |  |
| **SLATE** | P | 82 | 115 | 1st | a writing tablet | 0.57 | 0.90 | 0.44 |  |  |
|  |  |  |  | 2nd | thin layers of rock | 0.32 |  |  |  |  |
|  |  |  |  | 3rd | color | 0.10 |  |  |  |  |
| **SLIDE** | P | 95 | 89 | 1st | playground equipment | 0.79 | 0.96 | 0.79 |  |  |
|  |  |  |  | 2nd | skid/move sideways | 0.17 |  |  |  |  |
|  |  |  |  | 3rd | presentation panel | 0.04 |  |  |  |  |
| **SMOKE** | P | 93 | 87 | 1st | inhale and exhale smoke from cigarettes | 0.53 | 1.00 | 0.11 |  |  |
|  |  |  |  | 2nd | a cloud of fine particles suspended in a gas | 0.47 |  |  |  |  |
| **SNORT** | P | 93 | 83 | 1st | inhale through the nose | 0.57 | 0.92 | 0.38 |  |  |
|  |  |  |  | 2nd | a disrespectful laugh | 0.35 |  |  |  |  |
|  |  |  |  | 3rd | make a snorting sound by exhaling hard | 0.08 |  |  |  |  |
| **SODA** | P | 96 | 188 | 1st | pop/coke | 0.99 | 1.00 | 0.99 |  |  |
|  |  |  |  | 2nd | sodium carbonate | 0.01 |  |  |  |  |
| **SOLE** | H(+P) | 187 | 335 | 1st | foot/footwear | 0.73 | 0.97 | 0.66 |  |  |
|  |  |  |  | 2nd | alone/only | 0.24 |  |  |  |  |
|  |  |  |  | 3rd | fish | 0.03 |  |  |  |  |
| **SOLUTION** | P | 94 | 179 | 1st | solving a problem | 0.68 | 1.00 | 0.53 |  |  |
|  |  |  |  | 2nd | liquid mixture | 0.32 |  |  |  |  |
| **SPACE** | P | 92 | 170 | 1st | outside the Earth's atmosphere | 0.78 | 1.00 | 0.71 |  |  |
|  |  |  |  | 2nd | an empty area | 0.22 |  |  |  |  |
| **SPADE** | H(+P) | 95 | 165 | 1st | a playing card | 0.75 | 1.00 | 0.66 |  |  |
|  |  |  |  | 2nd | hand shovel | 0.25 |  |  |  |  |
| **SPANISH** | P | 92 | 142 | 1st | language | 0.61 | 1.00 | 0.37 |  |  |
|  |  |  |  | 2nd | people (culture) | 0.39 |  |  |  |  |
| **SPEAKER** | P | 191 | 328 | 1st | talker | 0.55 | 1.00 | 0.19 |  |  |
|  |  |  |  | 2nd | acoustic transducer | 0.45 |  |  |  |  |
| **SPEECH** | P | 189 | 339 | 1st | the act of delivering a formal spoken communication to an audience | 0.58 | 1.00 | 0.29 |  |  |
|  |  |  |  | 2nd | language/communication by word of mouth | 0.42 |  |  |  |  |
| **SPELL** | H(+P) | 95 | 176 | 1st | recite the letters of | 0.60 | 1.00 | 0.32 |  |  |
|  |  |  |  | 2nd | enchantment | 0.40 |  |  |  |  |
| **SPIT** | H(+P) | 96 | 184 | 1st | eject saliva | 0.98 | 1.00 | 0.98 |  |  |
|  |  |  |  | 2nd | skewer for holding meat | 0.02 |  |  |  |  |
| **SPOKE** | H(+P) | 96 | 93 | 1st | express in speech | 0.90 | 1.00 | 0.89 |  |  |
|  |  |  |  | 2nd | wheel | 0.10 |  |  |  |  |
| **SPONGE** | P | 96 | 78 | 1st | wipe with a sponge, so as to clean | 0.68 | 0.95 | 0.60 |  |  |
|  |  |  |  | 2nd | Sponge Bob | 0.27 |  |  |  |  |
|  |  |  |  | 3rd | animal | 0.05 |  |  |  |  |
| **SPOT** | P | 96 | 176 | 1st | speckle, small contrasting part of something | 0.68 | 0.85 | 0.74 |  |  |
|  |  |  |  | 2nd | blemish made by dirt | 0.18 |  |  |  |  |
|  |  |  |  | 3rd | location | 0.15 |  |  |  |  |
| **SPREAD** | P | 93 | 176 | 1st | tasty mixture to be spread on bread or crackers | 0.81 | 1.00 | 0.76 |  |  |
|  |  |  |  | 2nd | extending over a wide expanse of space | 0.19 |  |  |  |  |
| **SPRING** | P | 93 | 173 | 1st | season of growth | 0.93 | 1.00 | 0.93 |  |  |
|  |  |  |  | 2nd | metal elastic device | 0.07 |  |  |  |  |
| **SQUARE** | P | 95 | 166 | 1st | geometry | 0.87 | 1.00 | 0.86 |  |  |
|  |  |  |  | 2nd | an open area at the meeting of two or more streets | 0.13 |  |  |  |  |
| **SQUASH** | H(+P) | 95 | 187 | 1st | squash plant | 0.67 | 0.89 | 0.68 |  |  |
|  |  |  |  | 2nd | compress with violence | 0.21 |  |  |  |  |
|  |  |  |  | 3rd | game played with rackets | 0.11 |  |  |  |  |
| **STABLE** | H(+P) | 93 | 91 | 1st | horse barn | 0.56 | 1.00 | 0.22 |  |  |
|  |  |  |  | 2nd | firm and dependable | 0.44 |  |  |  |  |
| **STAFF** | H(+P) | 96 | 182 | 1st | personnel | 0.95 | 1.00 | 0.94 | P | 2nd WS Homonym def is obscure (building material); personnel def is polyseme |
|  |  |  |  | 2nd | strong rod or stick | 0.05 |  |  |  |  |
| **STALK** | H(+P) | 187 | 176 | 1st | follow stealthily | 0.69 | 1.00 | 0.55 |  |  |
|  |  |  |  | 2nd | plants | 0.31 |  |  |  |  |
| **STALL** | H(+P) | 95 | 90 | 1st | area set off by walls | 0.63 | 0.97 | 0.47 |  |  |
|  |  |  |  | 2nd | procrastinate | 0.33 |  |  |  |  |
|  |  |  |  | 3rd | car come to a stop | 0.03 |  |  |  |  |
| **STAR** | P | 189 | 359 | 1st | celestial body | 0.82 | 0.97 | 0.82 |  |  |
|  |  |  |  | 2nd | someone who is dazzlingly skilled | 0.15 |  |  |  |  |
|  |  |  |  | 3rd | shape | 0.03 |  |  |  |  |
| **STARBUCKS** | NL | 95 | 177 | 1st | coffee | 0.73 | 1.00 | 0.63 | P | Not listed in WS dictionary |
|  |  |  |  | 2nd | café/company | 0.27 |  |  |  |  |
| **STARCH** | P | 93 | 172 | 1st | food | 0.90 | 1.00 | 0.89 |  |  |
|  |  |  |  | 2nd | laundry | 0.10 |  |  |  |  |
| **STATE** | P | 95 | 183 | 1st | province in the US | 0.86 | 1.00 | 0.84 |  |  |
|  |  |  |  | 2nd | way something is with respect to its main attribute | 0.14 |  |  |  |  |
| **STEER** | H(+P) | 92 | 89 | 1st | maneuver | 0.80 | 1.00 | 0.75 |  |  |
|  |  |  |  | 2nd | bull | 0.20 |  |  |  |  |
| **STEP** | P | 96 | 164 | 1st | raising the foot and setting it down | 0.63 | 1.00 | 0.42 |  |  |
|  |  |  |  | 2nd | stairway | 0.37 |  |  |  |  |
| **STOCK** | P | 93 | 162 | 1st | ownership interest in a company | 0.46 | 0.77 | 0.32 |  |  |
|  |  |  |  | 2nd | inventory | 0.31 |  |  |  |  |
|  |  |  |  | 3rd | broth | 0.22 |  |  |  |  |
|  |  |  |  | 4th | animals | 0.01 |  |  |  |  |
| **STORE** | P | 96 | 93 | 1st | retail shop | 0.80 | 1.00 | 0.75 |  |  |
|  |  |  |  | 2nd | keep or lay aside for future use | 0.20 |  |  |  |  |
| **STORY** | H(+P) | 93 | 183 | 1st | narrative | 0.96 | 1.00 | 0.96 |  |  |
|  |  |  |  | 2nd | vertical level | 0.04 |  |  |  |  |
| **STRAW** | P | 94 | 163 | 1st | drinking | 0.64 | 1.00 | 0.43 |  |  |
|  |  |  |  | 2nd | plant fiber | 0.36 |  |  |  |  |
| **STRIKE** | P | 187 | 333 | 1st | attack | 0.30 | 0.59 | 0.05 |  |  |
|  |  |  |  | 2nd | baseball | 0.29 |  |  |  |  |
|  |  |  |  | 3rd | work stoppage | 0.25 |  |  |  |  |
|  |  |  |  | 4th | bowling | 0.16 |  |  |  |  |
| **STRINGS** | P | 95 | 179 | 1st | cord, thread | 0.57 | 1.00 | 0.25 |  |  |
|  |  |  |  | 2nd | musical instrument | 0.43 |  |  |  |  |
| **STRIP** | H(+P) | 191 | 180 | 1st | undress | 0.65 | 1.00 | 0.46 |  |  |
|  |  |  |  | 2nd | long narrow piece of something | 0.35 |  |  |  |  |
| **STROKE** | P | 95 | 175 | 1st | rupture or occlusion of a blood vessel | 0.75 | 0.88 | 0.83 |  |  |
|  |  |  |  | 2nd | move hand with gentle pressure | 0.13 |  |  |  |  |
|  |  |  |  | 3rd | swimming | 0.12 |  |  |  |  |
| **SUBJECT** | P | 94 | 164 | 1st | branch of knowledge, field of study | 0.66 | 0.95 | 0.58 |  |  |
|  |  |  |  | 2nd | topic of a conversation | 0.28 |  |  |  |  |
|  |  |  |  | 3rd | experiment participant | 0.05 |  |  |  |  |
| **SUBWAY** | P | 96 | 189 | 1st | restaurant | 0.60 | 1.00 | 0.33 | H | Restaurant name not listed in WS dictionary |
|  |  |  |  | 2nd | metro/underground train | 0.40 |  |  |  |  |
| **SUIT** | P | 94 | 172 | 1st | set of garments | 0.95 | 1.00 | 0.95 |  |  |
|  |  |  |  | 2nd | playing cards | 0.05 |  |  |  |  |
| **SUN** | P | 96 | 185 | 1st | Earth's star | 0.65 | 1.00 | 0.46 |  |  |
|  |  |  |  | 2nd | rays of the sun | 0.35 |  |  |  |  |
| **SWALLOW** | H(+P) | 189 | 359 | 1st | act of swallowing | 0.77 | 1.00 | 0.69 |  |  |
|  |  |  |  | 2nd | bird | 0.23 |  |  |  |  |
| **TAB** | P | 93 | 167 | 1st | (computerized) menu tab | 0.42 | 0.75 | 0.20 |  |  |
|  |  |  |  | 2nd | bill, check | 0.34 |  |  |  |  |
|  |  |  |  | 3rd | key on a keyboard | 0.25 |  |  |  |  |
| **TABLE** | P | 96 | 87 | 1st | piece of furniture | 0.99 | 1.00 | 0.99 |  |  |
|  |  |  |  | 2nd | array of data | 0.01 |  |  |  |  |
| **TAG** | H(+P) | 96 | 176 | 1st | game in which one child chases the others | 0.71 | 1.00 | 0.59 |  |  |
|  |  |  |  | 2nd | label written or printed | 0.29 |  |  |  |  |
| **TANGO** | P | 96 | 136 | 1st | ballroom dance | 0.97 | 1.00 | 0.97 |  |  |
|  |  |  |  | 2nd | music | 0.03 |  |  |  |  |
| **TAP** | P | 96 | 78 | 1st | strike lightly | 0.37 | 0.71 | 0.10 |  |  |
|  |  |  |  | 2nd | tap dancing | 0.33 |  |  |  |  |
|  |  |  |  | 3rd | faucet | 0.29 |  |  |  |  |
| **TAPE** | P | 96 | 180 | 1st | piece of something for binding/fastening (adhesive) | 0.69 | 0.91 | 0.69 |  |  |
|  |  |  |  | 2nd | recording | 0.22 |  |  |  |  |
|  |  |  |  | 3rd | measure | 0.09 |  |  |  |  |
| **TARGET** | P | 96 | 176 | 1st | retail store | 0.63 | 1.00 | 0.41 |  |  |
|  |  |  |  | 2nd | reference point to shoot at | 0.37 |  |  |  |  |
| **TART** | H(+P) | 96 | 73 | 1st | sourish | 0.53 | 1.00 | 0.13 |  |  |
|  |  |  |  | 2nd | pastry | 0.47 |  |  |  |  |
| **TEAR** | H(+P) | 96 | 189 | 1st | teardrop | 0.69 | 1.00 | 0.56 |  |  |
|  |  |  |  | 2nd | split | 0.31 |  |  |  |  |
| **TEASPOON** | P | 96 | 171 | 1st | what a teaspoon will hold | 0.54 | 1.00 | 0.16 |  |  |
|  |  |  |  | 2nd | a small spoon | 0.46 |  |  |  |  |
| **TEETH** | P | 93 | 181 | 1st | bonelike structures used for biting and chewing | 1.00 | 1.00 | 1.00 |  |  |
|  |  |  |  | 2nd | projections on a gear | 0.00 |  |  |  |  |
| **TELEVISION** | P | 93 | 166 | 1st | broadcasting visual images | 0.78 | 1.00 | 0.71 |  |  |
|  |  |  |  | 2nd | electronic device | 0.22 |  |  |  |  |
| **TEMPLE** | H(+P) | 95 | 186 | 1st | place of worship | 0.83 | 1.00 | 0.79 |  |  |
|  |  |  |  | 2nd | side of the forehead | 0.17 |  |  |  |  |
| **TERM** | P | 95 | 186 | 1st | a limited period of time | 0.76 | 1.00 | 0.68 |  |  |
|  |  |  |  | 2nd | a word or expression used for some particular thing | 0.24 |  |  |  |  |
| **TERMINAL** | P | 96 | 91 | 1st | station where transport vehicles load or unload passengers or goods | 0.60 | 0.98 | 0.38 |  |  |
|  |  |  |  | 2nd | causing or ending in or approaching death | 0.37 |  |  |  |  |
|  |  |  |  | 3rd | computer | 0.02 |  |  |  |  |
| **THEATER** | P | 96 | 190 | 1st | the art of writing and producing plays | 0.87 | 1.00 | 0.85 |  |  |
|  |  |  |  | 2nd | a building where theatrical performances or motion-picture shows can be presented | 0.13 |  |  |  |  |
| **THREADS** | P | 92 | 175 | 1st | a fine cord of twisted fibers (of cotton or silk or wool or nylon etc.) used in sewing and weaving | 0.69 | 0.93 | 0.65 |  |  |
|  |  |  |  | 2nd | informal terms for clothing | 0.24 |  |  |  |  |
|  |  |  |  | 3rd | the connections that link the various parts of an event or argument together | 0.07 |  |  |  |  |
| **TICK** | H(+P) | 190 | 341 | 1st | metallic tapping sound | 0.68 | 1.00 | 0.52 |  |  |
|  |  |  |  | 2nd | arachnids | 0.32 |  |  |  |  |
| **TIE** | P | 96 | 91 | 1st | necktie | 0.70 | 0.92 | 0.69 |  |  |
|  |  |  |  | 2nd | bind | 0.22 |  |  |  |  |
|  |  |  |  | 3rd | equal | 0.08 |  |  |  |  |
| **TIP** | H(+P) | 96 | 156 | 1st | gratuity | 0.79 | 0.92 | 0.84 |  |  |
|  |  |  |  | 2nd | edge/point | 0.13 |  |  |  |  |
|  |  |  |  | 3rd | confidential information | 0.08 |  |  |  |  |
| **TIRE** | H(+P) | 191 | 184 | 1st | wheel | 0.88 | 1.00 | 0.86 |  |  |
|  |  |  |  | 2nd | fatigue | 0.13 |  |  |  |  |
| **TITLE** | P | 191 | 351 | 1st | name of a work of art or literary composition etc. | 0.73 | 0.98 | 0.66 |  |  |
|  |  |  |  | 2nd | an identifying appellation signifying status or function | 0.25 |  |  |  |  |
|  |  |  |  | 3rd | ownership | 0.02 |  |  |  |  |
| **TOAST** | H(+P) | 96 | 183 | 1st | slices of bread | 0.91 | 1.00 | 0.90 |  |  |
|  |  |  |  | 2nd | a drink in honor of or to the health of a person or event | 0.09 |  |  |  |  |
| **TOES** | P | 96 | 185 | 1st | digits of the foot | 1.00 | 1.00 | 1.00 |  |  |
|  |  |  |  | 2nd | the part of footwear that provides a covering for the toes | 0.00 |  |  |  |  |
| **TONGUE** | P | 96 | 189 | 1st | tissue in the oral cavity | 0.97 | 1.00 | 0.97 |  |  |
|  |  |  |  | 2nd | language | 0.03 |  |  |  |  |
| **TOYOTA** | NL | 94 | 173 | 1st | car | 0.83 | 1.00 | 0.79 | P | Car brand name is not listed in WS dictionary |
|  |  |  |  | 2nd | company | 0.17 |  |  |  |  |
| **TRACE** | H(+P) | 95 | 86 | 1st | make a mark or lines on a surface | 0.64 | 1.00 | 0.44 | P | 2nd Homonym def is obscure (rope/chain) |
|  |  |  |  | 2nd | a just detectable amount | 0.36 |  |  |  |  |
| **TRACK** | P | 93 | 84 | 1st | a course over which races are run | 0.82 | 0.92 | 0.88 |  |  |
|  |  |  |  | 2nd | a groove on a phonograph recording | 0.10 |  |  |  |  |
|  |  |  |  | 3rd | go after with the intent to catch | 0.08 |  |  |  |  |
| **TRIAL** | P | 96 | 179 | 1st | the determination of a person's innocence or guilt by due process of law | 0.64 | 1.00 | 0.43 |  |  |
|  |  |  |  | 2nd | the act of testing something | 0.36 |  |  |  |  |
| **TRIANGLE** | P | 96 | 180 | 1st | a three-sided polygon | 0.77 | 1.00 | 0.70 |  |  |
|  |  |  |  | 2nd | something approximating the shape of a triangle | 0.23 |  |  |  |  |
| **TRIP** | P | 95 | 185 | 1st | a journey | 0.66 | 1.00 | 0.50 |  |  |
|  |  |  |  | 2nd | an accidental misstep threatening (or causing) a fall | 0.34 |  |  |  |  |
| **TROUT** | P | 96 | 116 | 1st | fishes of cool fresh waters | 0.91 | 1.00 | 0.90 |  |  |
|  |  |  |  | 2nd | flesh of any of several primarily freshwater game and food fishes | 0.09 |  |  |  |  |
| **TRUNK** | P | 95 | 176 | 1st | compartment in an automobile | 0.43 | 0.65 | 0.48 |  |  |
|  |  |  |  | 2nd | main stem of a tree | 0.22 |  |  |  |  |
|  |  |  |  | 3rd | elephant | 0.17 |  |  |  |  |
|  |  |  |  | 4th | luggage | 0.18 |  |  |  |  |
| **TUB** | P | 95 | 182 | 1st | bathtub | 0.77 | 0.95 | 0.78 |  |  |
|  |  |  |  | 2nd | what a tub will hold | 0.17 |  |  |  |  |
|  |  |  |  | 3rd | vat | 0.05 |  |  |  |  |
| **TUNA** | H(+P) | 94 | 141 | 1st | (food) served as steaks | 0.56 | 1.00 | 0.20 | P | 2nd WS Homonym def is obscure (cactus) |
|  |  |  |  | 2nd | warm-water fish | 0.44 |  |  |  |  |
| **TURKEY** | H(+P) | 96 | 174 | 1st | fowl usually roasted | 0.78 | 0.96 | 0.77 |  |  |
|  |  |  |  | 2nd | bird with fan-shaped tail | 0.18 |  |  |  |  |
|  |  |  |  | 3rd | country | 0.04 |  |  |  |  |
| **TURN** | P | 95 | 91 | 1st | change orientation or direction | 0.96 | 1.00 | 0.95 |  |  |
|  |  |  |  | 2nd | game | 0.04 |  |  |  |  |
| **TURQUOISE** | P | 96 | 129 | 1st | color | 0.78 | 1.00 | 0.72 |  |  |
|  |  |  |  | 2nd | mineral | 0.22 |  |  |  |  |
| **TYPE** | P | 188 | 344 | 1st | printed characters | 0.53 | 1.00 | 0.13 |  |  |
|  |  |  |  | 2nd | subdivision of a particular kind of thing | 0.47 |  |  |  |  |
| **UNIFORM** | P | 92 | 85 | 1st | clothing of distinctive design | 0.78 | 1.00 | 0.71 |  |  |
|  |  |  |  | 2nd | always the same | 0.22 |  |  |  |  |
| **UNIVERSITY** | P | 189 | 217 | 1st | large and diverse institution of higher learning | 0.71 | 1.00 | 0.58 |  |  |
|  |  |  |  | 2nd | establishment where a seat of higher learning is housed | 0.29 |  |  |  |  |
| **URCHIN** | P | 95 | 184 | 1st | (sea urchin) shallow-water echinoderms | 0.92 | 1.00 | 0.92 |  |  |
|  |  |  |  | 2nd | poor and often mischievous city child | 0.08 |  |  |  |  |
| **VATICAN** | P | 92 | 170 | 1st | the authority of the Pope | 0.51 | 1.00 | 0.05 |  |  |
|  |  |  |  | 2nd | residence of the Catholic Pope | 0.49 |  |  |  |  |
| **VEGAS** | NL | 96 | 191 | 1st | entertainment and gambling and general excess | 0.77 | 1.00 | 0.71 | P | City name is not listed in WS dictionary |
|  |  |  |  | 2nd | city in Nevada | 0.23 |  |  |  |  |
| **VESSEL** | P | 95 | 158 | 1st | craft designed for water transportation | 0.62 | 0.84 | 0.65 |  |  |
|  |  |  |  | 2nd | a tube in which a body fluid circulates | 0.22 |  |  |  |  |
|  |  |  |  | 3rd | container | 0.13 |  |  |  |  |
|  |  |  |  | 4th | religion | 0.04 |  |  |  |  |
| **VET** | H(+P) | 96 | 178 | 1st | veterinarian | 0.67 | 1.00 | 0.50 |  |  |
|  |  |  |  | 2nd | veteran | 0.33 |  |  |  |  |
| **VIETNAM** | P | 96 | 175 | 1st | war | 0.65 | 0.91 | 0.60 |  |  |
|  |  |  |  | 2nd | country | 0.26 |  |  |  |  |
|  |  |  |  | 3rd | food | 0.09 |  |  |  |  |
| **VIOLET** | P | 93 | 86 | 1st | color | 0.73 | 1.00 | 0.63 |  |  |
|  |  |  |  | 2nd | flowers | 0.27 |  |  |  |  |
| **VOLKSWAGEN** | NL | 189 | 360 | 1st | car | 0.80 | 1.00 | 0.75 | P | Car brand name is not listed in WS dictionary |
|  |  |  |  | 2nd | brand/company | 0.20 |  |  |  |  |
| **VOLLEYBALL** | P | 96 | 171 | 1st | game | 0.95 | 1.00 | 0.95 |  |  |
|  |  |  |  | 2nd | an inflated ball | 0.05 |  |  |  |  |
| **VOLUME** | P | 94 | 172 | 1st | loudness | 0.72 | 0.97 | 0.65 |  |  |
|  |  |  |  | 2nd | amount of 3-dimensional space | 0.25 |  |  |  |  |
|  |  |  |  | 3rd | book | 0.01 |  |  |  |  |
|  |  |  |  | 4th | hair | 0.02 |  |  |  |  |
| **WALL STREET** | P | 96 | 166 | 1st | The securities industry of the United States | 0.89 | 1.00 | 0.87 |  |  |
|  |  |  |  | 2nd | a street in lower Manhattan | 0.11 |  |  |  |  |
| **WALNUT** | P | 96 | 147 | 1st | edible nut | 0.81 | 1.00 | 0.76 |  |  |
|  |  |  |  | 2nd | tree | 0.19 |  |  |  |  |
| **WALTZ** | P | 94 | 155 | 1st | a ballroom dance | 0.90 | 1.00 | 0.89 |  |  |
|  |  |  |  | 2nd | music | 0.10 |  |  |  |  |
| **WATCH** | P | 96 | 188 | 1st | small portable timepiece | 0.61 | 1.00 | 0.37 |  |  |
|  |  |  |  | 2nd | look attentively | 0.39 |  |  |  |  |
| **WAVE** | P | 95 | 173 | 1st | ridges across a body of water | 0.83 | 1.00 | 0.79 |  |  |
|  |  |  |  | 2nd | act of signaling by a movement of the hand | 0.17 |  |  |  |  |
| **WEED** | H(+P) | 192 | 301 | 1st | marijuana | 0.67 | 1.00 | 0.52 | P | 2nd WS Homonym def is obscure (mourning apparel) |
|  |  |  |  | 2nd | any plant that crowds out cultivated plants | 0.33 |  |  |  |  |
| **WELL** | H(+P) | 96 | 170 | 1st | a deep hole or shaft dug or drilled to obtain a liquid | 0.59 | 1.00 | 0.30 |  |  |
|  |  |  |  | 2nd | in good health | 0.41 |  |  |  |  |
| **WHEELS** | P | 95 | 44 | 1st | rotating discs on a vehicle | 0.80 | 1.00 | 0.74 |  |  |
|  |  |  |  | 2nd | a wheeled vehicle | 0.20 |  |  |  |  |
| **WHISTLE** | P | 96 | 178 | 1st | device that produces a loud shrill sound | 0.61 | 1.00 | 0.37 |  |  |
|  |  |  |  | 2nd | the sound | 0.39 |  |  |  |  |
| **WHITE HOUSE** | P | 95 | 180 | 1st | chief executive department of the US government | 0.82 | 1.00 | 0.78 |  |  |
|  |  |  |  | 2nd | the government building | 0.18 |  |  |  |  |
| **WILL** | H(+P) | 96 | 139 | 1st | a fixed and persistent intent or purpose | 0.47 | 0.94 | 0.03 |  |  |
|  |  |  |  | 2nd | a legal document | 0.46 |  |  |  |  |
|  |  |  |  | 3rd | future tense | 0.06 |  |  |  |  |
| **WIMBLEDON** | P | 82 | 142 | 1st | annual international tennis championships | 0.72 | 1.00 | 0.61 |  |  |
|  |  |  |  | 2nd | a suburb of London | 0.28 |  |  |  |  |
| **WIND** | H(+P) | 191 | 169 | 1st | air moving | 0.99 | 1.00 | 0.99 |  |  |
|  |  |  |  | 2nd | weave, meander | 0.01 |  |  |  |  |
| **WINDOW** | P | 96 | 161 | 1st | a framework of wood or metal hat contains a glass | 0.63 | 1.00 | 0.41 |  |  |
|  |  |  |  | 2nd | opening in a wall or screen that admits light and air | 0.37 |  |  |  |  |
| **WONDER** | P | 96 | 144 | 1st | curiosity | 0.52 | 1.00 | 0.08 |  |  |
|  |  |  |  | 2nd | marvel, amazement | 0.48 |  |  |  |  |
| **WOOD** | P | 93 | 132 | 1st | trees | 0.67 | 1.00 | 0.50 |  |  |
|  |  |  |  | 2nd | cut and prepared for use as a building material | 0.33 |  |  |  |  |
| **WOOL** | P | 95 | 167 | 1st | a fabric | 0.65 | 1.00 | 0.45 |  |  |
|  |  |  |  | 2nd | outer coat of especially sheep | 0.35 |  |  |  |  |
| **YARD** | H(+P) | 96 | 178 | 1st | the enclosed land around a house | 0.67 | 1.00 | 0.52 |  |  |
|  |  |  |  | 2nd | unit of length equal to 3 feet | 0.33 |  |  |  |  |
| **YARN** | P | 96 | 181 | 1st | twisted fibers | 0.97 | 1.00 | 0.97 |  |  |
|  |  |  |  | 2nd | narration | 0.03 |  |  |  |  |
| **ZIP** | H(+P) | 95 | 168 | 1st | a fastener for locking together two toothed edges by means of a sliding tab | 0.54 | 1.00 | 0.13 |  |  |
|  |  |  |  | 2nd | code | 0.46 |  |  |  |  |
|  |  |  |  |  |  |  |  |  |  |  |
| ***CHANEL** | P | 48 |  |  |  |  |  |  |  | Some Ss confused for CHANNEL |
| ***CHEEK** | P | 95 |  |  |  |  |  |  |  | All responses aligned with single meaning |
| ***CONFLICT** | P | 189 |  |  |  |  |  |  |  | All responses aligned with single meaning |
| ***CUBE** | P | 96 |  |  |  |  |  |  |  | All responses aligned with single meaning |
| ***DEPARTMENT** | P | 189 |  |  |  |  |  |  |  | All responses aligned with single meaning |
| ***DUCT** | P | 92 |  |  |  |  |  |  |  | Response meanings indistinguishable |
| ***EMBASSY** | P | 95 |  |  |  |  |  |  |  | Response meanings indistinguishable |
| ***FEATHERS** | P | 96 |  |  |  |  |  |  |  | All responses aligned with single meaning |
| ***FLIGHT** | H(+P) | 96 |  |  |  |  |  |  |  | Response meanings indistinguishable |
| ***GOLF** | P | 94 |  |  |  |  |  |  |  | Response meanings indistinguishable |
| ***ROSES** | H(+P) | 96 |  |  |  |  |  |  |  | Experimenter error: should have been ROSE |
