# Supplementary material for: Offline dominance and zeugmatic similarity normings of variably ambiguous words assessed against a neural language model (BERT)
Source: Behav Res Methods. 2022 Jun 10;55(4):1537–57. doi: 10.3758/s13428-022-01869-6 (PMC10040203; doi:10.3758/s13428-022-01869-6)
Supplement: Supplementary file 3 — (DOCX 82.4 kb) [file 13428_2022_1869_MOESM3_ESM.docx]

| **APPENDIX 2. STUDY 2 SIMILARITY NORMING SUMMARY DATA** | | | | | | | |
| --- | --- | --- | --- | --- | --- | --- | --- |
| Similarity norming stimuli and ratings averaged over 88 respondents. COLUMN A. CW refers to Critical Word. COLUMN B: Dominance Score refers to dominance score calculated as ([Most Dominant minus Second Most Dominant]/Most Dominant); 0=perfectly balanced and 1=completely biased. Dominance scores marked by an asterisk* indicate values exceeding 0.79 for which alternative dominance scores from Armstrong, Tokowicz, & Plaut (2012) were referenced to meet our norming selection criterion. COLUMN C: Alternative Dominance Score from Armstrong et al. (2012) eDom refers to item dominance scores taken from Armstrong et al. (2012). COLUMN D: Similarity Norming Category refers to the ambiguous word classification used for this norming (H=homonym, IP=irregular polyseme, RP=regular polyseme, UA=unambiguous). COLUMN E: Zeugmatic Similarity Norming Sentence refers to Zeugmatic test sentence using most dominant definitions from dominance norming, when available. COLUMN F: Similarity Mean refers to average similarity score across participants on a scale of 1-7, with 1 representing the least meaning/sense similarity and 7 the most. COLUMN G: Similarity STDEV refers to similarity rating standard deviation. COLUMN H: Similarity SEM refers to similarity rating standard error of the mean. Grayed items indicate an experimenter error in constructing the norming sentence with those items excluded from analysis. | | | | | | | |
| COLUMN A | B | C | D | E | F | G | H |
|  |  |  |  |  |  |  |  |
| CW | Dominance Score | Alternative Dominance Score from Armstrong et al. (2012) eDom | Similarity Norming Category | Zeugmatic Similarity Norming Sentence | Similarity Mean | Similarity STDEV | Similarity SEM |
|  |  |  |  |  |  |  |  |
| ACROBATS | NA |  | UA | At the circus we watched the **ACROBATS** and at the gym we met **them.** | 6.24 | 1.41 | 0.15 |
| ADMIT | 0.22 |  | IP | The truth was **ADMITTED** and the law students were **admitted,** too. | 2.42 | 1.39 | 0.15 |
| AMAZON | 0.57 |  | H | Adventurers trekked the **AMAZON** and online retailers sold products **there.** | 1.48 | 1.02 | 0.11 |
| ANKLE | NA |  | UA | Tom got a tattoo on his **ANKLE** and the soccer player twisted **his.** | 6.57 | 1.01 | 0.11 |
| ANSWER | 0.15 |  | IP | She never got an **ANSWER** for her question or **one** for the math problem. | 5.99 | 1.25 | 0.13 |
| APPEAL | 0.42 |  | IP | Her personal style has an **APPEAL** and the defendant was granted **one.** | 1.91 | 1.45 | 0.15 |
| ARMS | 0.75 |  | H | The weary soldiers lay down their **ARMS** and my brothers got tattoos on **theirs.** | 2.42 | 1.87 | 0.20 |
| ASPIRIN | NA |  | UA | The doctor prescribed **ASPIRIN** and I always keep **some** handy. | 6.70 | 0.61 | 0.06 |
| AUNT | NA |  | UA | I stayed with my **AUNT** and received a gift from **her.** | 6.66 | 0.96 | 0.10 |
| AUTUMN | NA |  | UA | The kids returned to school in **AUTUMN** and there were several storms **then.** | 6.00 | 1.42 | 0.15 |
| BACK | 0.22 |  | RP | Extra boxes are stored in the **BACK** and I got a tattoo on **mine.** | 2.35 | 1.24 | 0.13 |
| BAG | 0.76 |  | RP | My brother carried a **BAG** of Cheetos and I ate the **whole** **thing.** | 6.22 | 1.35 | 0.14 |
| BALANCE | 0.55 |  | IP | With the weights there was a **BALANCE** and also **one** with checking account. | 2.10 | 1.21 | 0.13 |
| BAND | 0.73 |  | H | The teenagers formed a **BAND** and the married man wore **one.** | 1.58 | 1.04 | 0.11 |
| BAR | 0.69 |  | IP | A man was seated at the **BAR** and between classes I ate **one.** | 1.69 | 1.21 | 0.13 |
| BARK | 0.26 |  | H | The trees had a rough **BARK** and the poodle had a loud **one.** | 1.31 | 0.82 | 0.09 |
| BASE | 0.42 |  | IP | The **BASE** of the tree was rotted and the outfielder tagged **one.** | 1.77 | 1.03 | 0.11 |
| BASEMENT | NA |  | UA | Our laundry is in the **BASEMENT** and there is flooding **there.** | 6.66 | 0.76 | 0.08 |
| BASKETBALL | 0.67 |  | RP | My brother dribbled the **BASKETBALL** and I watched **some** on TV. | 5.03 | 1.50 | 0.16 |
| BASS | 0.69 |  | H | The fisherman caught the **BASS** and the musician played **one.** | 1.34 | 0.79 | 0.08 |
| BAT | 0.51 |  | H | The player swung a **BAT** and the vampire was bit by **one.** | 1.60 | 1.36 | 0.15 |
| BATTER | 0.66 |  | H | The catcher tagged the **BATTER** and the baker mixed **some.** | 1.34 | 0.93 | 0.10 |
| BEACH | NA |  | UA | I walked on the **BEACH** and tourists flocked **there.** | 6.52 | 0.82 | 0.09 |
| BEAM | 0.28 |  | IP | The roof was supported by a **BEAM** and **one** shone through my window. | 2.22 | 1.48 | 0.16 |
| BEATLES | 0.03 |  | RP | My grandmother met the **BEATLES** and I listened to **them.** | 6.44 | 1.09 | 0.12 |
| BEETHOVEN | 0.64 |  | RP | The classical pianist performed **BEETHOVEN** and owned some music annotated by **him.** | 6.22 | 1.08 | 0.11 |
| BEGGARS | NA |  | UA | The streets were filled with **BEGGARS** and we gave coins to some of **them.** | 6.67 | 0.99 | 0.11 |
| BILL | 0.28 |  | H | There are two **BILLS** in my class and **some** in my wallet. | 1.63 | 1.24 | 0.13 |
| BITTERNESS | 0.40 |  | IP | The prisoner had some **BITTERNESS** and the coffee had **some,** too. | 3.28 | 1.46 | 0.16 |
| BLUFF | 0.68 |  | H | The poker player called the **BLUFF** and the weapons were positioned on **one.** | 1.58 | 0.85 | 0.09 |
| BOND | 0.42 |  | H | Super Glue creates a **BOND** and Sean Connery played **him** in the movies. | 1.41 | 0.87 | 0.09 |
| BOOK | 0.23 |  | RP | She was completely absorbed by the **BOOK** and flipped **its** pages. | 6.69 | 0.91 | 0.10 |
| BOTTLE | 0.20 |  | RP | The man drank a **BOTTLE** and his friend smashed **one.** | 6.19 | 0.99 | 0.11 |
| BOW | 0.05 |  | H | The archer shot the **BOW** and the kindergartner practiced tying **one.** | 1.69 | 1.10 | 0.12 |
| BOWLER | 0.78 |  | H | My uncle was a **BOWLER** and the English gentleman wore **one.** | 1.63 | 1.18 | 0.13 |
| BRACELET | NA |  | UA | She wore a **BRACELET** and received **one** as a gift. | 6.52 | 1.01 | 0.11 |
| BRASS | 0.19 |  | IP | The marching band was mostly **BRASS** and the light fixtures were **brass,** too. | 3.23 | 1.73 | 0.18 |
| BREAK | 0.04 |  | IP | Employers recommend workers get a **BREAK** and X-rays showed I had **one.** | 1.72 | 0.97 | 0.10 |
| BRIEFCASE | 0.64 |  | RP | He carried his **BRIEFCASE** and she organized **hers.** | 6.38 | 1.10 | 0.12 |
| BRUSH | Not normed | 0.51 | H | The hikers got lost in the **BRUSH** and I detangled my hair with **one.** | 1.65 | 1.15 | 0.12 |
| BUCK | Not normed | 0.54 | H | The customer paid a **BUCK** and the hunter shot **one.** | 1.31 | 0.70 | 0.07 |
| BUCKET | 0.22 |  | RP | The maid poured a **BUCKET** and the kids carried **one.** | 5.59 | 1.51 | 0.16 |
| BUD | 0.56 |  | H | The flower sprouted a **BUD** and the drunk dude drank **one.** | 1.26 | 0.63 | 0.07 |
| CABBAGE | 0.48 |  | RP | The gardener grew **CABBAGE** and the children ate **some.** | 6.47 | 1.17 | 0.13 |
| CABINET | 0.69 |  | IP | The President met with his **CABINET** and we put cups in **ours.** | 1.53 | 1.06 | 0.11 |
| CALF | 0.33 |  | H | The goalie kicked my **CALF** and the cow birthed **one.** | 1.32 | 0.70 | 0.08 |
| CANOE | NA |  | UA | We drifted in a **CANOE** and the museum displayed **one.** | 5.61 | 1.71 | 0.18 |
| CARDINAL | 0.69 |  | IP | The Pope appointed a **CARDINAL** and we spotted **one** in a nest. | 1.68 | 1.27 | 0.14 |
| CARTON | 0.20 |  | RP | The guy smoked a whole **CARTON** and warning labels covered **it.** | 5.63 | 1.85 | 0.20 |
| CASE | 0.35 |  | H | The lawyer appealed a **CASE** and the wine connoisseur bought **one.** | 1.50 | 0.92 | 0.10 |
| CAST | 0.43 |  | IP | The audience applauded members of the **CAST** and the doctor removed **mine.** | 1.38 | 0.91 | 0.10 |
| CAULDRON | 0.65 |  | RP | We emptied the **CAULDRON** and others lifted **one.** | 5.44 | 1.81 | 0.19 |
| CEDAR | 0.58 |  | RP | Hikers tramped through the **CEDAR** and carpenters use **it** for closets. | 4.51 | 1.81 | 0.19 |
| CHANGE | 0.69 |  | IP | We adapted to the **CHANGE** and the store clerk counted **it.** | 1.63 | 1.23 | 0.13 |
| CHARACTER | 0.66 |  | IP | Impatience is a flaw in her **CHARACTER** and little dialogue was spoken by **mine.** | 3.32 | 1.79 | 0.19 |
| CHARM | 0.23 |  | IP | My Southern grandma is known for her **CHARM** and Sienna's bracelet had **one.** | 1.85 | 1.09 | 0.12 |
| CHECK | 0.63 |  | IP | The mechanic performed a **CHECK** and the renter wrote **one.** | 1.45 | 0.97 | 0.10 |
| CHICKEN | 0.72 |  | RP | The **CHICKEN** clucked and **it** smelled delicious. | 5.17 | 1.60 | 0.17 |
| CHIMNEY | 0.76 |  | RP | Santa slid down our neighbor's **CHIMNEY** and owls perched on **ours.** | 6.43 | 1.00 | 0.11 |
| CHINESE | 0.52 |  | RP | The restaurant's cuisine was **CHINESE** and **it** was also my first language. | 4.60 | 1.55 | 0.17 |
| CHURCH | 0.59 |  | RP | A decree was announced by the **CHURCH** and a bell hung in **another.** | 5.20 | 1.72 | 0.18 |
| CIDER | NA |  | UA | The waitress brought some **CIDER** and we drank **some.** | 6.76 | 0.69 | 0.07 |
| CIGARETTES | NA |  | UA | My mom hates the smell of **CIGARETTES** and my father quit smoking **them.** | 6.85 | 0.44 | 0.05 |
| CIGARS | NA |  | UA | He bought **CIGARS** in Cuba and they smoked **some.** | 6.75 | 0.73 | 0.08 |
| CLOG | 0.40 |  | IP | The woman wore **CLOGS** and the plumber unstopped **them.** | 1.48 | 1.07 | 0.11 |
| COCKROACHES | NA |  | UA | We spotted **COCKROACHES** at night and the exterminator killed **some.** | 6.77 | 0.64 | 0.07 |
| COFFIN | NA |  | UA | They chose a pine **COFFIN** and the pall bearers carried **one.** | 5.38 | 1.98 | 0.21 |
| COLON | 0.64 |  | H | The cancer spread to the **COLON** and the grammar editor added **one.** | 1.28 | 0.69 | 0.07 |
| COLUMN | 0.16 |  | IP | The porch was supported by a **COLUMN** and the numbers were listed in **one.** | 2.42 | 1.30 | 0.14 |
| CORAL | 0.64 |  | RP | The clothing racks were full of **CORAL** and the Australian reef had **a lot**, too. | 2.49 | 1.60 | 0.17 |
| CORE | 0.09 |  | IP | They drilled through Earth to its **CORE** and chucked the **one** from the apple. | 3.45 | 1.77 | 0.19 |
| CORN | 0.29 |  | H | The foot doctor examined my **CORN** and the farmer picked **some.** |  |  |  |
| CORPSE | NA |  | UA | The search party found a **CORPSE** and the medical examiner uncovered **one.** | 6.42 | 1.16 | 0.12 |
| COTTAGE | NA |  | UA | The honeymooners rented the **COTTAGE** and we walked near **there.** | 6.13 | 1.25 | 0.13 |
| COTTON | 0.49 |  | RP | The fashion designer stitched the **COTTON** and the migrant workers picked **it.** | 5.63 | 1.45 | 0.15 |
| COUNT | 0.57 |  | H | The Englishman bowed to the **COUNT** and the election board tallied **it.** | 1.76 | 1.22 | 0.13 |
| COUNTER | 0.56 |  | H | The realtor responded to the **COUNTER** and the clerk leaned on **one.** | 1.88 | 1.38 | 0.15 |
| COURT | 0.63 |  | IP | The attorneys argued the case in the **COURT** and the tennis player volleyed on **one.** | 2.02 | 1.18 | 0.13 |
| CRAB | Not normed | 0.52 | H | My grumpy sister was a **CRAB** and the fisherman netted **one.** | 2.35 | 1.33 | 0.14 |
| CRANE | 0.31 |  | IP | At the wetlands we spotted a **CRANE** and the cement blocks were lifted by **one.** | 1.66 | 1.19 | 0.13 |
| CREAM | Not normed | Not available | RP | We painted the room **CREAM** and I drank my coffee with **some.** | 2.51 | 1.43 | 0.15 |
| CRICKET | 0.64 |  | H | The Brits played **CRICKET** and the bug collector caught **one.** | 1.44 | 0.87 | 0.09 |
| CUE | 0.70 |  | H | The pool player grabbed a **CUE** and the stage actor waited for **hers.** | 1.66 | 1.00 | 0.11 |
| CUP | 0.45 |  | RP | The customer drank a **CUP** and the artist painted **one.** | 4.81 | 1.80 | 0.19 |
| CURSE | 0.38 |  | IP | The boy shouted a **CURSE** and the wizard put **one** on us. | 3.47 | 1.79 | 0.19 |
| CYCLE | 0.18 |  | IP | Water evaporation is part of a **CYCLE** and the biker hopped onto **one.** | 1.85 | 1.08 | 0.11 |
| DAD | NA |  | UA | The car was purchased by my **DAD** and I introduced my friends to **him.** | 6.61 | 1.00 | 0.11 |
| DANDRUFF | NA |  | UA | My curls hide my **DANDRUFF** and the shampoo helps with **it.** | 6.44 | 1.19 | 0.13 |
| DANISH | 0.42 |  | IP | My relatives are **DANISH** and for breakfast I ate **one.** | 1.94 | 1.04 | 0.11 |
| DAUGHTER | NA |  | UA | The college accepted my **DAUGHTER** and my sister cradled **hers.** | 5.81 | 1.60 | 0.17 |
| DAYLIGHT | NA |  | UA | The model looked different in the **DAYLIGHT** and vampires avoid **it.** | 5.93 | 1.59 | 0.17 |
| DECK | 0.67 |  | IP | Cards were dealt from the **DECK** and the sailor worked below **one.** | 1.60 | 1.01 | 0.11 |
| DEED | 0.21 |  | IP | The boy scout performed a **DEED** and the property owner received **his.** | 2.00 | 1.17 | 0.13 |
| DEGREE | 0.42 |  | IP | The temperature rose a **DEGREE** and the biology major pursued **one.** | 1.47 | 0.91 | 0.10 |
| DENTIST | NA |  | UA | He was studying to be a **DENTIST** and I made an appointment with **one.** | 6.24 | 1.34 | 0.14 |
| DEW | NA |  | UA | The cobweb glistened in the **DEW** and my shoes got wet from **it.** | 6.26 | 1.19 | 0.13 |
| DIET | 0.40 |  | IP | She lost weight on her **DIET** and eliminated gluten from **it.** | 5.80 | 1.44 | 0.15 |
| DIFFERENCE | 0.54 |  | IP | Subtracting two numbers gave her the **DIFFERENCE** and there was a notable **one** in her face after surgery. | 3.22 | 1.67 | 0.18 |
| DIP | 0.54 |  | IP | At the river they took a **DIP** and at the potluck they sampled **one.** | 1.86 | 1.19 | 0.13 |
| DOOR | 0.26 |  | RP | The student walked through the **DOOR** and someone knocked on **it.** | 6.50 | 1.17 | 0.13 |
| DOZEN | NA |  | UA | The baker selected a **DOZEN** and the car collector purchased a **dozen.** | 5.32 | 1.79 | 0.19 |
| DRAFT | 0.55 |  | IP | The English major finished the **DRAFT** and the football player entered **one.** | 1.64 | 1.03 | 0.11 |
| DRILL | 0.33 |  | IP | The soldiers took part in a **DRILL** and the carpenter used **one** to tighten screws. | 1.74 | 1.34 | 0.14 |
| DRINK | 0.59 |  | IP | The kids ran to the fountain for a **DRINK** and the man at the bar ordered **one.** | 5.06 | 1.61 | 0.17 |
| DROP | 0.52 |  | IP | My cell phone survived several **DROPS** and the rainstorm began with a **few.** | 2.17 | 1.44 | 0.15 |
| DRUMSTICK | 0.23 |  | IP | The percussionist played with **DRUMSTICKS** and the Thanksgiving guest ate **some.** | 1.73 | 1.12 | 0.12 |
| DUCHESS | NA |  | UA | Dignitaries greeted the **DUCHESS** and photos were taken of **her.** | 6.61 | 0.93 | 0.10 |
| DUSK | NA |  | UA | The surveyors quit working at **DUSK** and the light was beautiful **then.** | 6.35 | 1.17 | 0.12 |
| DYE | NA |  | UA | At Easter they bought some **DYE** and the hair colorist also used **some.** | 5.73 | 1.13 | 0.12 |
| EARTHWORMS | NA |  | UA | The fisherman grabbed some **EARTHWORMS** and the gardens benefited from **them.** | 6.17 | 1.30 | 0.14 |
| EASEL | NA |  | UA | The painter set up an **EASEL** and the poster rested on **one.** | 5.88 | 1.65 | 0.18 |
| EGGS | 0.73 |  | RP | For breakfast I ate several **EGGS** and the turtle laid **some.** | 5.13 | 1.46 | 0.16 |
| ELVES | NA |  | UA | Santa was assisted by the **ELVES** and wizards are respected by **them.** | 5.20 | 1.70 | 0.18 |
| ELVIS | 0.60 |  | RP | I am related to **ELVIS** and the radio station plays **him** quite a bit. | 6.11 | 1.16 | 0.12 |
| EMERALD | 0.57 |  | RP | The paint color was **EMERALD** and the ring was set with **one.** | 3.91 | 1.40 | 0.15 |
| FAN | 0.67 |  | H | The store was cooled by **FANS** and the rockstar was pestered by **them.** | 1.82 | 1.41 | 0.15 |
| FAT | 0.64 |  | IP | The professional athlete had no **FAT** and the Greek yogurt didn't have **any** either. | 4.53 | 1.60 | 0.17 |
| FAULT | 0.19 |  | IP | The court determined the **FAULT** and the earthquake centered on **one.** | 1.72 | 1.12 | 0.12 |
| FIREPLACE | NA |  | UA | The dog rested by a **FIREPLACE** and the kids roasted marshmallows in **one.** | 6.56 | 0.87 | 0.09 |
| FISH | 0.74 |  | RP | They snorkeled with some **FISH** and ate **some** for dinner. | 6.19 | 1.07 | 0.11 |
| FLASK | Not normed | Not available | RP | The skier guzzled a **FLASK** and my brother dropped **his.** | 5.10 | 1.79 | 0.19 |
| FLEAS | NA |  | UA | My dog has **FLEAS** and we spotted **some.** | 6.49 | 1.06 | 0.11 |
| FOREHEAD | NA |  | UA | The nurse felt my **FOREHEAD** and my mom kissed **it.** | 6.52 | 1.23 | 0.13 |
| FORTUNE | 0.48 |  | IP | The millionaire made his **FORTUNE** and the oracle told me **mine.** | 2.58 | 1.79 | 0.19 |
| FROWN | NA |  | UA | The circus clown had a **FROWN** and the unhappy man had **one,** too. | 6.57 | 0.98 | 0.11 |
| FUNCTION | 0.24 |  | IP | The decorative drawings had no **FUNCTION** but the algebra problem was calculated using **one.** | 2.24 | 1.45 | 0.15 |
| FUR | 0.59 |  | IP | The pet groomer combed the **FUR** and the runway model wore **one.** | 4.59 | 1.53 | 0.16 |
| FURNACE | NA |  | UA | He was near the **FURNACE** and the service technician inspected **it.** | 5.98 | 1.57 | 0.17 |
| GAG | 0.69 |  | H | The prank was a **GAG** and the kidnapper's handkerchief was **one,** too. | 2.05 | 1.45 | 0.15 |
| GALLONS | NA |  | UA | The attendant pumped a few **GALLONS** and the athletes drank **a few.** | 4.49 | 1.86 | 0.20 |
| GATE | 0.44 |  | RP | My dad repainted the **GATE** and my ball rolled through **it.** | 6.23 | 1.39 | 0.15 |
| GEAR | 0.02 |  | IP | The driver shifted the **GEAR** and the backpacker unpacked **his.** | 1.91 | 1.37 | 0.15 |
| GERMAN | 0.50 |  | RP | My ancestors are **GERMAN** and **that** is what they mostly speak. | 5.31 | 1.41 | 0.15 |
| GLACIER | NA |  | UA | The satellite monitored the **GLACIER** and the hikers scrambled across **it.** | 5.61 | 1.69 | 0.18 |
| GLARE | 0.50 |  | H | The oncoming headlights caused a **GLARE** and my brother shot me **one.** | 2.24 | 1.31 | 0.14 |
| GOAL | 0.17 |  | IP | The football player scored a **GOAL** and the student with straight A's met **hers.** | 3.30 | 1.61 | 0.17 |
| GRACE | 0.45 |  | IP | The Catholic family began meals with **GRACE** and the ballerina danced with **it.** | 1.75 | 1.06 | 0.11 |
| GRANDFATHER | NA |  | UA | The advertisement featured a **GRANDFATHER** and Roger is **one** now. | 6.11 | 1.47 | 0.16 |
| GRANDMOTHER | NA |  | UA | The locket belonged to her **GRANDMOTHER** and the girl dearly loved **her.** | 6.80 | 0.68 | 0.07 |
| GUIDE | 0.64 |  | IP | We thumbed the pages of our **GUIDE** and the tour was led by **one.** | 3.28 | 1.58 | 0.17 |
| HAMSTER | NA |  | UA | The kids fed one **HAMSTER** and everyone searched for **the other.** | 6.11 | 1.36 | 0.14 |
| HATCHET | NA |  | UA | The woodsman used the **HATCHET** and our neighbor borrowed **it.** | 6.45 | 0.95 | 0.10 |
| HAY | NA |  | UA | The farmer pitched the **HAY** and the horses slept on **it.** | 6.40 | 1.22 | 0.13 |
| HEART | 0.60 |  | RP | There was a medical problem with his **HEART** and for Valentine's we decorated **one.** | 3.56 | 1.53 | 0.16 |
| HELMET | NA |  | UA | For safety I grabbed a **HELMET** and the other biker wore **one.** | 6.66 | 0.86 | 0.09 |
| HEMINGWAY | 0.44 |  | RP | Our class read **HEMINGWAY** and we met **his** grandchildren. | 5.69 | 1.52 | 0.16 |
| HEXAGON | NA |  | UA | Six sides formed a **HEXAGON** and the student traced **one.** | 6.39 | 1.10 | 0.12 |
| HIGHWAY | NA |  | UA | The semi truck exited the **HIGHWAY** and trees were planted along **it.** | 6.32 | 1.31 | 0.14 |
| HIP-HOP | 0.43 |  | RP | The young dance students prefer **HIP-HOP** and musicians like to perform **it.** | 6.27 | 1.09 | 0.12 |
| HIROSHIMA | 0.42 |  | RP | Tokyo is larger than **HIROSHIMA** and the explosion was larger than **it,** too. | 5.25 | 1.66 | 0.18 |
| HOE | NA |  | UA | She grabbed a **HOE** and chopped weeds with **it.** | 6.43 | 1.28 | 0.14 |
| HOLLYWOOD | 0.53 |  | RP | The vice cop hated **HOLLYWOOD** and the foreign director hated **it,** too. | 5.86 | 1.42 | 0.15 |
| HOSPITAL | 0.01 |  | RP | There was a call from the **HOSPITAL** and we drove to **it.** | 6.48 | 0.95 | 0.10 |
| HOSTAGE | NA |  | UA | They took a **HOSTAGE** and wanted ransom for **him.** | 6.55 | 1.13 | 0.12 |
| ICICLE | NA |  | UA | I bumped into the **ICICLE** and water dripped from **it.** | 6.41 | 1.21 | 0.13 |
| IGLOO | NA |  | UA | Neighbor kids built an **IGLOO** and Eskimos lived in **one.** | 6.27 | 0.93 | 0.10 |
| INFANT | NA |  | UA | Their child was an **INFANT** and the other couple adopted **one.** | 6.44 | 1.21 | 0.13 |
| INTRODUCTION | 0.12 |  | IP | The book began with an **INTRODUCTION** but the couple met without **one.** | 4.09 | 1.73 | 0.18 |
| IRAQ | 0.51 |  | RP | The politicians discussed **IRAQ** and the travelers walked **its** border. | 6.03 | 1.12 | 0.12 |
| IRON | 0.52 |  | IP | The prospector mined the **IRON** and the maid unplugged **it.** | 1.74 | 1.06 | 0.11 |
| ISSUE | 0.54 |  | IP | That bookstore carried several **ISSUES** and the mental patients had lots of **them.** | 1.91 | 1.52 | 0.16 |
| IVORY | 0.64 |  | RP | The room was painted **IVORY** and the knife was carved from **it.** | 3.02 | 1.51 | 0.16 |
| IVY | NA |  | UA | The buildings were covered in **IVY** and tall elms grew in **it.** | 5.41 | 1.70 | 0.18 |
| JADE | 0.35 |  | RP | The color of the room was **JADE** and her jewelry was set with **some,** too. | 4.03 | 1.58 | 0.17 |
| JANITOR | NA |  | UA | Trash was emptied by the **JANITOR** and the school fired **him.** | 6.65 | 0.82 | 0.09 |
| JAPANESE | 0.27 |  | RP | They dined out on **JAPANESE** and his best friend is **Japanese.** | 4.82 | 1.56 | 0.17 |
| JEEP | NA |  | UA | Soldiers piled into the **JEEP** and the teenager crashed **one.** | 6.19 | 1.23 | 0.13 |
| JINGLE | 0.55 |  | IP | On Christmas Eve we heard a **JINGLE** and the **one** on the commercial was catchy. | 5.33 | 1.69 | 0.18 |
| JUG | 0.59 |  | RP | He poured a **JUG** and she lifted **one.** | 5.50 | 1.63 | 0.17 |
| JUGGLER | NA |  | UA | The talent show began with a **JUGGLER** and for the party they hired **one.** | 6.34 | 1.27 | 0.14 |
| KEG | 0.35 |  | RP | The frat boys drank a **KEG** and the other guys lifted **one.** | 5.65 | 1.55 | 0.16 |
| KITTEN | NA |  | UA | The young children gently stroked the **KITTEN** and we heard **its** meow. | 6.69 | 0.82 | 0.09 |
| KOREAN | 0.73 |  | RP | Her favorite cuisine was **KOREAN** and most of her friends were **Korean.** | 5.17 | 1.46 | 0.16 |
| LACE | 0.50 |  | IP | Her gown was trimmed with **LACE** and I reached for my shoe to tie **mine.** | 2.28 | 1.24 | 0.13 |
| LAMB | 0.67 |  | RP | The farmer fed the **LAMB** and the diners ate **some.** | 6.00 | 1.21 | 0.13 |
| LAP | 0.56 |  | H | The children sat on our **LAPS** and the runners ran several of **them.** | 1.42 | 1.05 | 0.11 |
| LAVENDER | 0.40 |  | RP | She painted the room **LAVENDER** and they enjoyed **its** scent. | 3.63 | 1.58 | 0.17 |
| LEAD | 0.22 |  | H | The fast runner took the **LEAD** and the **one** in the pencil broke. | 1.31 | 0.75 | 0.08 |
| LEAN | 0.42 |  | H | The dieter ate only **LEAN** and the engineers corrected the **one** in the structure. | 1.31 | 0.81 | 0.09 |
| LEATHER | NA |  | UA | Her pants were made from **LEATHER** and we couldn't distinguish **it** from vinyl. | 5.59 | 1.82 | 0.19 |
| LEMONADE | NA |  | UA | The kids were selling some **LEMONADE** and we sat and drank **some.** | 6.72 | 0.80 | 0.09 |
| LIBRARY | 0.31 |  | RP | Their uncle read the entire **LIBRARY** and the structural engineer toured **it.** | 5.02 | 1.74 | 0.19 |
| LILAC | Not normed | Not available | RP | The bedroom was **LILAC** and the flower gardens smelled strongly of **it.** | 3.93 | 1.62 | 0.17 |
| LIMBO | Not normed | 0.67 | H | Indecision left the businessman in **LIMBO** and the kids on skates danced **one.** | 1.94 | 1.34 | 0.14 |
| LOBSTER | 0.04 |  | RP | The fisherman caught a **LOBSTER** and for dinner we ate **one.** | 6.49 | 1.12 | 0.12 |
| LORD | 0.05 |  | IP | The countess curtsied to the **LORD** and the church goers prayed to **theirs.** | 3.19 | 1.94 | 0.21 |
| LUNGS | NA |  | UA | A deep breath inflated his **LUNGS** and doctors found cancer in **them.** | 6.51 | 0.95 | 0.10 |
| MAPLE | 0.55 |  | RP | We love the flavor of **MAPLE** and the contractor appreciates **its** hardness. | 2.90 | 1.74 | 0.19 |
| MARBLE | 0.28 |  | RP | The stone cutter selected the **MARBLE** and the kitten played with **one.** | 2.25 | 1.38 | 0.15 |
| MARCH | 0.52 |  | H | His birthday was in **MARCH** and the protestors attended **one.** | 1.38 | 0.82 | 0.09 |
| MASS | 0.69 |  | H | Priests performed the **MASS** and scientists quantified **it.** | 1.51 | 1.09 | 0.12 |
| MATCH | 0.13 |  | H | The smoker lit a **MATCH** and the soccer team played **one.** | 1.42 | 0.99 | 0.11 |
| MATTRESS | NA |  | UA | He lay down on the **MATTRESS** and kids jumped on **it.** | 6.55 | 1.18 | 0.13 |
| MEADOW | NA |  | UA | The landscape painting depicted a **MEADOW** and we picked flowers in **one.** | 6.48 | 1.03 | 0.11 |
| MEASLES | NA |  | UA | I have the symptoms of **MEASLES** but there is little chance of **them.** | 6.00 | 1.63 | 0.17 |
| MEDITERRANEAN | 0.50 |  | RP | The diners opted for **MEDITERRANEAN** and we vacationed **there.** | 4.15 | 1.58 | 0.17 |
| MERMAID | NA |  | UA | The story was about a **MERMAID** and the girl swam like **one.** | 6.41 | 0.92 | 0.10 |
| METER | Not normed | 0.36 | H | The distance was a **METER** and we put some coins in **one.** | 1.70 | 1.41 | 0.15 |
| MEXICAN | 0.18 |  | RP | Her family is **MEXICAN** and the food is **Mexican,** too. | 5.55 | 1.19 | 0.13 |
| MIDNIGHT | NA |  | UA | The boys snuck out at **MIDNIGHT** and it was pitch black **then.** | 6.43 | 1.12 | 0.12 |
| MINE | 0.11 |  | H | The book was **MINE** and the coal worker dug in **one.** | 1.22 | 0.60 | 0.06 |
| MINT | *0.96 | 0.52 | H | Most coins are produced at the **MINT** and tea is flavored with **it.** | 1.47 | 1.12 | 0.12 |
| MISS | 0.18 |  | H | Every basketball shot was a **MISS** and the young lady was **one.** | 1.41 | 0.93 | 0.10 |
| MISSISSIPPI | 0.03 |  | RP | My cousins live in **MISSISSIPPI** and I once canoed **it.** | 4.03 | 1.69 | 0.18 |
| MOLD | 0.79 |  | H | Mildew spray removed the **MOLD** and concrete was poured into **one.** | 1.69 | 1.28 | 0.14 |
| MOLE | 0.12 |  | H | The dermatologist discovered a **MOLE** and the gardener trapped **one.** | 1.45 | 0.91 | 0.10 |
| MONITOR | 0.45 |  | IP | The graphics were displayed on a **MONITOR** and the students were supervised by **one.** | 2.05 | 1.33 | 0.14 |
| MONK | NA |  | UA | The prayer service was led by a **MONK** and I led the quiet life of **one.** | 5.75 | 1.42 | 0.15 |
| MORGUE | NA |  | UA | The doctor mostly worked in the **MORGUE** and bodies were examined **there.** | 6.60 | 1.10 | 0.12 |
| MOSQUITOS | NA |  | UA | The nets kept away the **MOSQUITOS** and I killed several of **them.** | 6.73 | 0.66 | 0.07 |
| MOSS | NA |  | UA | The stones were covered in **MOSS** and tiny mushrooms grew out of **it.** | 6.14 | 1.36 | 0.14 |
| MOTHS | NA |  | UA | In the closet were several **MOTHS** and the outdoor light attracted **them.** | 6.35 | 1.37 | 0.15 |
| MUSIC | 0.37 |  | IP | She loved to listen to **MUSIC** and **that** was her major. | 5.74 | 1.24 | 0.13 |
| MUSTANG | 0.46 |  | H | The auto dealer sold a **MUSTANG** and the horse rancher tamed **one.** | 2.01 | 1.20 | 0.13 |
| MYTH | NA |  | UA | The story was just a **MYTH** and Zeus is described in **one.** | 6.10 | 1.27 | 0.14 |
| NAIL | 0.58 |  | IP | She chipped a **NAIL** and he hammered **one.** | 1.72 | 1.16 | 0.12 |
| NAVY | 0.68 |  | RP | My brother joined the **NAVY** and my car was **that** color. | 1.57 | 1.03 | 0.11 |
| NECKLACE | NA |  | UA | My grandma left me a **NECKLACE** and my earrings matched **it.** | 6.49 | 1.27 | 0.14 |
| NET | Not normed | 0.57 | H | The accountant calculated the **NET** and the tightrope walker worked without **one.** | 1.47 | 0.87 | 0.09 |
| NIGHTGOWN | NA |  | UA | My aunt was still in her **NIGHTGOWN** and was freezing in **it.** | 6.40 | 1.26 | 0.13 |
| NIRVANA | 0.16 |  | H | The yogi reached a state of **NIRVANA** and the guitarist was a fan of **them.** | 2.03 | 1.19 | 0.13 |
| NOMADS | NA |  | UA | The oasis was settled by **NOMADS** and the college students wandered like **them.** | 5.43 | 1.51 | 0.16 |
| NOON | NA |  | UA | The campus bells chimed at **NOON** and we met for lunch **then.** | 6.53 | 1.07 | 0.11 |
| OAK | 0.47 |  | RP | He cut some branches from the **OAK** and the woodworker sanded **some.** | 5.78 | 1.38 | 0.15 |
| OAR | NA |  | UA | On his wall hung an **OAR** and the rower paddled with **one.** | 5.80 | 1.69 | 0.18 |
| OCEAN | NA |  | UA | We could smell the **OCEAN** and our room overlooks **it.** | 6.53 | 1.02 | 0.11 |
| ODD | 0.48 |  | IP | All the numbers were **ODD** and the scientists were **odd**, too. | 1.98 | 1.28 | 0.14 |
| OIL | 0.38 |  | IP | The engine needed some **OIL** and the salad needed **some**, too. | 2.92 | 1.52 | 0.16 |
| OPENING | 0.21 |  | IP | Visitors entered through an **OPENING** and the films' stars attended **one.** | 2.77 | 1.49 | 0.16 |
| OPERATION | 0.54 |  | IP | The Coast Guard initiated the **OPERATION** and the surgeons performed **one.** | 3.56 | 1.60 | 0.17 |
| ORANGE | 0.53 |  | RP | Her jacket was **ORANGE** and she peeled herself **one.** | 2.70 | 1.58 | 0.17 |
| ORDER | 0.44 |  | IP | The waitress wrote down the **ORDER** and the army sergeant gave **one.** | 3.56 | 1.65 | 0.18 |
| OWL | NA |  | UA | We heard the hoot of an **OWL** and a mouse was killed by **it.** | 6.61 | 1.01 | 0.11 |
| PALACE | 0.12 |  | RP | A statement was issued by the **PALACE** and roads were closed near **it.** | 5.60 | 1.61 | 0.17 |
| PALM | 0.15 |  | H | The landscape crew trimmed the **PALMS** and the fortune teller read **ours.** | 1.52 | 1.23 | 0.13 |
| PANEL | 0.61 |  | IP | Indicator lights were on the **PANEL** and several experts in the field were on **one,** too. | 2.43 | 1.42 | 0.15 |
| PASSAGE | 0.37 |  | IP | Each student read aloud a **PASSAGE** and the tourists walked along **one.** | 1.60 | 0.93 | 0.10 |
| PENTAGON | 0.08 |  | RP | The vice admiral visited the **PENTAGON** and the geometry student drew **one.** | 2.69 | 1.57 | 0.17 |
| PICASSO | 0.52 |  | RP | The museum collected **PICASSO** and I once met **his** granddaughter. | 5.50 | 1.42 | 0.15 |
| PIGEONS | NA |  | UA | Messages were carried by the **PIGEONS** and a homeless man fed **them.** | 6.31 | 1.27 | 0.14 |
| PIPE | 0.66 |  | IP | The smoker pulled out a **PIPE** and the plumber replaced **one.** | 2.34 | 1.33 | 0.14 |
| PIT | *0.80 | 0.61 | H | He gnawed the plum to the **PIT** and threw some charcoal in **one.** | 1.67 | 1.12 | 0.12 |
| PITCH | 0.18 |  | H | The batter swung at the **PITCH** and the musicians tried matching **it.** | 1.49 | 0.99 | 0.11 |
| PITCHER | 0.00 |  | H | Strikes were thrown by the **PITCHER** and water poured from **it.** | 1.44 | 0.96 | 0.10 |
| POACH | 0.05 |  | H | The rhinoceros was **POACHED** by hunters and the eggs by the **chef.** |  |  |  |
| POOL | *0.91 | 0.40 | H | The billiards expert played **POOL** and the children swam in **one.** | 1.51 | 0.99 | 0.11 |
| PORT | *0.82 | 0.36 | H | The wine connoisseur drank the **PORT** and the cruise ship entered **one.** | 1.59 | 1.05 | 0.11 |
| POST | 0.41 |  | H | On Facebook she liked the **POST** and the mailman gathered letters from **it.** | 2.08 | 1.30 | 0.14 |
| POT | 0.55 |  | H | She smoked a little **POT** and warmed the sauce in **one.** | 1.32 | 0.78 | 0.08 |
| POUND | 0.59 |  | H | The Englishman paid five **POUNDS** and my puppy weighed **that.** | 1.97 | 1.47 | 0.16 |
| PRESENT | 0.32 |  | H | He tried living in the **PRESENT** and she wrapped **it.** | 1.43 | 0.78 | 0.08 |
| PRISON | 0.07 |  | RP | The uniform was issued by the **PRISON** and a criminal tunneled through **it.** | 5.44 | 1.71 | 0.18 |
| PUNCH | 0.40 |  | H | The partygoer sipped the **PUNCH** and the boxer threw **one.** | 1.53 | 1.11 | 0.12 |
| PUPIL | 0.37 |  | H | The teacher questioned the **PUPIL** and the optometrist dilated **one.** | 1.52 | 1.09 | 0.12 |
| PYRAMID | 0.64 |  | RP | Tourists rode camels to a **PYRAMID** and sophomores calculated **one's** volume. | 3.82 | 1.82 | 0.19 |
| QUACK | 0.77 |  | H | The duck let out a **QUACK** and the fake doctor was **one.** | 1.57 | 0.94 | 0.10 |
| RACE | 0.30 |  | H | The woman's **RACE** was Caucasian and the runner ran **one.** | 1.40 | 1.03 | 0.11 |
| RACKET | 0.58 |  | H | The tennis player tried a **RACKET** and the loud neighbors were making **one.** | 1.63 | 0.89 | 0.09 |
| RARE | *0.84 | 0.45 | H | His grilled steak was **RARE** and the antique coin was **rare,** too. | 1.60 | 1.10 | 0.12 |
| REACTION | 0.31 |  | IP | The photographer captured the child's **REACTION** and the chemist monitored **one,** too. | 2.90 | 1.41 | 0.15 |
| RECIPE | Not normed | Not available | RP | The housewife cooked the **RECIPE** and the kids tasted **it.** | 5.86 | 1.42 | 0.15 |
| RESERVATION | 0.62 |  | IP | The luxury hotel confirmed the **RESERVATION** and the Navajo tribe lived on **one.** | 1.94 | 1.29 | 0.14 |
| REVIEW | 0.33 |  | IP | The critic wrote the **REVIEW** and before finals the undergrads attended **one.** | 2.99 | 1.47 | 0.16 |
| RING | 0.79 |  | H | The bell let out a **RING** and her fiancée bought **one.** | 1.72 | 1.20 | 0.13 |
| ROCK | 0.49 |  | H | The concert goers listened to **ROCK** and the landscaper designed with **it.** | 1.58 | 1.00 | 0.11 |
| RUBY | 0.24 |  | RP | Her hair was **RUBY** and there was **one** in her engagement ring. | 3.28 | 1.52 | 0.16 |
| RULER | 0.70 |  | IP | The country is governed by a **RULER** and the line measured with **one.** | 1.44 | 0.92 | 0.10 |
| SANDALS | NA |  | UA | The tourist bought **SANDALS** and she wore **them.** | 6.85 | 0.70 | 0.08 |
| SCENE | 0.27 |  | IP | The Broadway actors rehearsed the **SCENE** and the firemen raced to **one.** | 3.25 | 1.56 | 0.17 |
| SEAL | 0.67 |  | H | The Navy deployed the **SEALS** and at the beach **one** flicked its tail. | 1.50 | 0.84 | 0.09 |
| SECOND | 0.37 |  | H | The first battery was replaced by a **SECOND** and the runner was beat by **one.** | 2.18 | 1.66 | 0.18 |
| SHAKESPEARE | 0.62 |  | RP | Her favorite poetry was **SHAKESPEARE** and **he** was a distant relative. | 5.56 | 1.49 | 0.16 |
| SHEET | 0.08 |  | IP | The student wrote something on the **SHEET** and the maid laundered **one.** | 2.11 | 1.22 | 0.13 |
| SILVER | Not normed | Not available | RP | Her dress was **SILVER** and the coin was made of **it.** | 3.59 | 1.51 | 0.16 |
| SOLUTION | 0.53 |  | IP | The chemists mixed up a **SOLUTION** and the teachers arrived at **one.** | 2.15 | 1.22 | 0.13 |
| SPADE | 0.66 |  | H | The gardener used a **SPADE** and the poker player drew **one.** | 2.15 | 1.52 | 0.16 |
| SPANISH | 0.37 |  | RP | My grandmother is **SPANISH** and **it** is her native language. | 5.22 | 1.55 | 0.17 |
| SPEAKER | 0.19 |  | IP | The stereo system was missing a **SPEAKER** and the conference organizers scheduled **one.** | 2.38 | 1.35 | 0.14 |
| STALL | 0.47 |  | H | The group's delay tactic was a **STALL** and the horse was kept in **one.** | 1.74 | 1.47 | 0.16 |
| STAPLE | Not normed | 0.59 | H | The papers were fastened by a **STAPLE** and for college students ramen is **one.** | 1.53 | 0.90 | 0.10 |
| STARBUCKS | 0.63 |  | RP | The woman drank **STARBUCKS** and worked **there,** too. | 5.89 | 1.25 | 0.13 |
| STEP | 0.42 |  | IP | The baby took several **STEPS** and the grandmother slowly climbed **some.** | 3.91 | 1.85 | 0.20 |
| STRAW | 0.43 |  | IP | The patient sucked water through the **STRAW** and the horse nestled in **some.** | 1.59 | 0.88 | 0.09 |
| STRINGS | 0.25 |  | IP | The orchestra featured some **STRINGS** and the puppeteers pulled **several.** | 3.32 | 1.62 | 0.17 |
| STUD | Not normed | 0.52 | H | He hammered the nail into a **STUD** and my muscular boyfriend was **one.** | 1.74 | 1.28 | 0.14 |
| SUBWAY | 0.33 |  | H | We ordered sandwiches from **SUBWAY** and the commuters rode on **one.** | 1.43 | 0.97 | 0.10 |
| SUN | 0.46 |  | IP | I calculated the distance to the **SUN** and felt **it** on my arm. | 5.38 | 1.64 | 0.17 |
| SWALLOW | 0.69 |  | H | In the nest there was a **SWALLOW** and I opened the medicine bottle and took **one.** | 1.31 | 0.88 | 0.09 |
| TAG | 0.59 |  | H | The children played **TAG** and the new shirt still had **one.** | 1.56 | 1.04 | 0.11 |
| TAN | Not normed | Not available | RP | The paint she picked was **TAN** and in the summer she always gets **one.** | 3.63 | 1.56 | 0.17 |
| TARGET | 0.41 |  | IP | Her arrow hit the **TARGET** and my mom shops at **one.** | 1.76 | 1.01 | 0.11 |
| TART | 0.13 |  | H | She preferred sweet lemonade to **TART** and the pastry chef baked **one.** | 3.20 | 1.83 | 0.19 |
| TEAR | 0.56 |  | H | The girl shed a **TEAR** and the faded jeans had **one.** | 1.48 | 0.97 | 0.10 |
| TEASPOON | 0.16 |  | RP | She ate a **TEASPOON** and polished **one.** | 3.53 | 1.63 | 0.17 |
| TELEVISION | 0.71 |  | RP | I set the little cactus on the **TELEVISION** and then watched **some.** | 5.78 | 1.38 | 0.15 |
| TEMPLE | 0.79 |  | H | For holy holidays she visits her **TEMPLE** and for headaches she massages **it.** | 1.49 | 1.03 | 0.11 |
| TICK | 0.52 |  | H | The clock emitted a **TICK** and the hiker was bit by **one.** | 1.39 | 0.79 | 0.08 |
| TIP | *0.84 | 0.26 | H | He cut his finger on the **TIP** and the restaurant bill didn't include **one.** | 1.28 | 0.50 | 0.05 |
| TRIP | 0.50 |  | IP | The adventurous travelers took a **TRIP** and my sprained ankle was caused by **one.** | 1.86 | 1.31 | 0.14 |
| TULIPS | NA |  | UA | She grows all kinds of **TULIPS** and arranged a bouquet of **them.** | 6.50 | 1.16 | 0.12 |
| TUNA | 0.18 |  | RP | The fisherman caught some **TUNA** and we ate **some** for lunch. | 6.40 | 0.88 | 0.09 |
| TUNNEL | Not normed | Not available | RP | We drove through the **TUNNEL** and engineers built **it** with steel. | 6.42 | 1.00 | 0.11 |
| TURQUOISE | 0.72 |  | RP | The red didn't coordinate with **TURQUOISE** but the jewelry was inlaid with **it.** | 4.35 | 1.68 | 0.18 |
| UNIVERSITY | 0.58 |  | RP | The policy was implemented by the **UNIVERSITY** and ivy covered **it.** | 4.24 | 2.08 | 0.22 |
| VASE | Not normed | Not available | RP | The florist arranged the **VASE** and a sculptor carved **it.** | 5.52 | 1.57 | 0.17 |
| VATICAN | 0.05 |  | RP | Representatives were sent by the **VATICAN** and murals covered **it.** | 4.83 | 1.85 | 0.20 |
| VAULT | Not normed | 0.60 | H | The American gymnast medaled in **VAULT** and the banker put money in **one.** | 1.49 | 1.05 | 0.11 |
| VESSEL | 0.65 |  | IP | His stroke occurred from a ruptured **VESSEL** and the ship's captain steered **one.** | 1.60 | 1.15 | 0.12 |
| VET | 0.50 |  | H | Our cat was treated by a **VET** and the military agency was run by **one.** | 1.69 | 1.15 | 0.12 |
| VIETNAM | 0.60 |  | RP | My uncle protested **VIETNAM** and my sister vacationed **there.** | 5.14 | 1.76 | 0.19 |
| VIOLET | 0.63 |  | RP | The florist chose **VIOLETS** and the paint store carried a range of **them.** | 3.85 | 1.68 | 0.18 |
| VOLUME | 0.65 |  | IP | We asked the DJ to increase the **VOLUME** and for the geometric figure we calculated **it.** | 1.61 | 0.98 | 0.10 |
| WATCH | 0.37 |  | IP | The guard kept a **WATCH** and the businessman wore **one.** | 2.32 | 1.59 | 0.17 |
| WEED | 0.52 |  | IP | They used a bong to smoke the **WEED** and the gardener stooped to pull **one.** | 2.34 | 1.28 | 0.14 |
| WHISTLE | 0.37 |  | RP | The tea kettle let out a **WHISTLE** and the assault victim carried **one.** | 3.32 | 1.65 | 0.18 |
| WIG | NA |  | UA | In the portrait Washington wore a **WIG** and for Halloween I will wear **one.** | 6.28 | 1.46 | 0.16 |
| WILL | 0.03 |  | H | The attorney read a **WILL** and the exhausted hiker had **none.** | 2.00 | 1.29 | 0.14 |
| WINDOW | 0.41 |  | RP | The janitor wiped down the **WINDOW** and a breeze blew through **it.** | 6.42 | 1.12 | 0.12 |
| WOOL | 0.45 |  | RP | The ranchers sheared the **WOOL** and the laundry service dry cleaned **it.** | 4.79 | 1.71 | 0.18 |
| YARD | 0.52 |  | H | The quarterback ran for a **YARD** and the rental house had **one.** | 1.99 | 1.37 | 0.15 |
